# Viral genetics and transmission dynamics in the second wave of mpox outbreak in Portugal and forecasting public health scenarios

**DOI:** 10.1080/22221751.2024.2412635

**Published:** 2024-10-03

**Authors:** Rita Cordeiro, Constantino P. Caetano, Daniel Sobral, Rita Ferreira, Luís Coelho, Ana Pelerito, Isabel Lopes de Carvalho, Sónia Namorado, Dinis B. Loyens, Ricardo Mexia, Cândida Fernandes, José Miguel Neves, Ana Luísa João, Miguel Rocha, Luís Miguel Duque, Inês Correia, Teresa Baptista, Cláudia Brazão, Diogo Sousa, Paulo Filipe, Miguel Alpalhão, Fernando Maltez, Diana Póvoas, Raquel Pinto, João Caria, Rita Patrocínio de Jesus, Patrícia Pacheco, Francesca Peruzzu, Josefina Méndez, Luís Ferreira, Kamal Mansinho, João Vaz Alves, Joana Vasconcelos, João Domingos, Sara Casanova, Frederico Duarte, Maria João Gonçalves, Mafalda Brito Salvador, Mafalda Andresen Guimarães, Sueila Martins, Marvin Silva Oliveira, Daniela Santos, Luís Vieira, Maria Sofia Núncio, Vítor Borges, João Paulo Gomes

**Affiliations:** a Emergency Response and Biopreparedness Unit, Department of Infectious Diseases, National Institute of Health Doutor Ricardo Jorge (INSA), Lisbon, Portugal; b Institute of Environmental Health, Faculty of Medicine, University of Lisbon, Lisbon, Portugal; c Department of Epidemiology, National Institute of Health Doutor Ricardo Jorge (INSA), Lisbon, Portugal; d Genomics and Bioinformatics Unit, Department of Infectious Diseases, National Institute of Health Doutor Ricardo Jorge (INSA), Lisbon, Portugal; e Serviço de Dermatovenereologia, Consulta de DST, Unidade Local de Saúde de São José, Lisbon, Portugal; f GAT - Grupo de Ativistas em Tratamentos, GAT-CheckpointLX, Lisbon, Portugal; g Serviço de Infeccologia, Hospital Garcia de Orta, Almada, Portugal; h GAT - Grupo de Ativistas em Tratamentos, GAT-Intendente, Lisbon, Portugal; i Dermatology Department, Unidade Local de Saúde de Santa Maria, Lisbon, Portugal; j Dermatology Research Unit (PFilipe Lab), Instituto de Medicina Molecular João Lobo Antunes, University of Lisbon, Lisbon, Portugal; k Dermatology University Clinic, Faculty of Medicine, University of Lisbon, Lisbon, Portugal; l Serviço de Doenças Infeciosas, Hospital de Curry Cabral, Unidade Local de Saúde de São José, Lisbon, Portugal; m Instituto Gulbenkian de Ciência, Oeiras, Portugal; n Serviço de Infeciologia, Hospital Professor Doutor Fernando Fonseca, Unidade Local de Saúde Amadora/Sintra, Amadora, Portugal; o Serviço de Doenças Infecciosas, Centro Hospitalar Universitário de Santo António, Porto, Portugal; p Serviço de Doenças Infecciosas e Medicina Tropical, Hospital de Egas Moniz, Unidade Local de Saúde de Lisboa Ocidental, Lisbon, Portugal; q Serviço de Doenças Infeciosas, Hospital Pedro Hispano, Unidade Local de Saúde de Matosinhos, Matosinhos, Portugal; r Unidade de Doenças Sexualmente Transmissíveis, Unidade de Cuidados de Saúde Personalizados da Lapa, Unidade Local de Saúde de São José, Lisbon, Portugal; s PPCIRA, Unidade de Tratamento de Imunodeficiência, Hospital de Cascais, Lisboa, Portugal; t UL-PPCIRA, Unidade Local de Saúde Trás-os-Montes e Alto Douro, Vila Real, Portugal; u Serviço de Patologia Clínica, Unidade Local de Saúde do Tâmega e Sousa, Penafiel, Portugal; v Technology and Innovation Unit, Department of Human Genetics, National Institute of Health Doutor Ricardo Jorge (INSA), Lisbon, Portugal; w Veterinary and Animal Research Centre (CECAV), Faculty of Veterinary Medicine, Lusófona University, Lisbon, Portugal

**Keywords:** *Monkeypox virus*, second wave, MSM, high sexual activity, vaccination

## Abstract

In 2023, a second wave of the global mpox epidemic, which is mainly affecting men who have sex with men (MSM), was observed in some countries. Herein, we benefited from a large viral sequence sampling (76/121; 63%) and vast epidemiological data to characterise the re-emergence and circulation of the *Monkeypox virus* (MPXV) in Portugal during 2023. We also modelled transmission and forecasted public health scenarios through a compartmental susceptible-exposed-infectious-recovered (SEIR) model. Our results suggest that the 2023 mpox wave in Portugal resulted from limited introduction(s) of MPXV belonging to C.1.1 sublineage, hypothetically from Asia, followed by sustained viral transmission and potential exportation to other countries. We estimated that the contribution of the MSM high sexual activity group to mpox transmission was 120 (95% CrI: 30–3553) times higher than that of the low sexual activity group. However, among the high sexual activity group, vaccinated individuals likely contributed approximately eight times less [0.123 (95% CrI: 0.068–0.208)] than the unvaccinated ones. Vaccination was also linked to potential reduced disease severity, with a Mpox Severity Score of 6.0 in the vaccinated group compared to 7.0 in unvaccinated individuals. Scenario analysis indicated that transmission is highly sensitive to sexual behaviour, projecting that a slight increase in the MSM sub-population with high sexual activity can trigger new mpox waves. This study strongly supports that continued vaccination, targeted awareness among risk groups and routine genomic epidemiology is needed to anticipate and respond to novel MPXV threats (e.g. global dissemination of clade I viruses).

## Introduction

*Monkeypox virus* (MPXV) belongs to the *Poxviridae* family and *Orthopoxvirus* genus, including the smallpox virus. Historically, mpox has been a rare and endemic zoonotic disease in West and Central African countries. MPXV is known to be spread through close contact with lesions, body fluids and respiratory droplets of infected humans or animals [1,2]. However, since the beginning of May 2022, thousands of cases of mpox have been reported in several countries where the disease is not endemic, affecting primarily men who have sex with men (MSM). As of September 24, 2024, more than 106 thousand confirmed cases, including 234 deaths, have been reported by the World Health Organization (WHO) in 123 countries [3]. This global epidemic is associated with MPXV from clade IIb (sub-clade hMPXV1, lineage B.1), according to the new proposal for MPXV classification [4,5]. It is estimated that sustained hMPXV1 human-to-human transmission has been occurring since 2016, which represents a paradigm shift in MPXV ecology, evolution and epidemiology [6,7]. Clade IIb viruses are generally associated with milder disease and lower mortality rates [8], as observed during the ongoing global B.1 outbreak, in contrast with MPXV from clade I, which has active circulation mainly in the Democratic Republic of Congo [8–13]. On 14 August 2024, triggered by the considerable increase in the number of MPXV clade Ib infections in Democratic Republic of the Congo, accompanied by the geographical spread of the virus to neighbouring countries, the WHO declared mpox as a Public Health Emergency of International Concern [3]. Meanwhile, MPXV clade Ib cases outside Africa, namely in Sweden and Thailand, were reported in individuals with a history of travel to regions in Africa where the virus is actively circulating [3].

In general, most outbreaks of MPXV clade IIb have occurred during the summer, possibly due to higher mobility and participation in cultural and social activities during the holiday [14,15]. In particular, events or venues involving sexual contact have played a key role in the massive and global dissemination of MPXV [12,16–20]. The integration of genomic and epidemiological data, fuelled by transmission modelling, have further shown that individuals that engage in high-risk behaviours, e.g. anonymous sex with multiple partners and under-ascertainment of cases, may have contributed to initiating local outbreaks as well as a faster initial epidemic growth worldwide [12,16–20]. These studies also suggested that infection-induced immunity, herd immunity and sexual behaviour changes and awareness might have played important roles in the control of initial outbreaks of mpox [20–22]. In early July 2022, a Live Modified Vaccinia Virus Ankara (commercialised JYNNEOS® vaccine), a third-generation vaccine against smallpox, was approved in Europe for pre-exposure and post-exposure mpox prophylaxis. This vaccine has proven effectiveness in reducing the risk of mpox disease [23,24], especially in high-risk individuals, even though changes in behaviour within MSM populations (e.g. reduction in the number of sexual partners) have been identified as the main contributor to control dissemination during the first wave [20,21,25]. For instance, a study on mpox transmission during the early phases of the North American epidemic showed that cases declined before more than 10% of high-risk individuals in the USA had vaccine-induced immunity [19].

Portugal reported the first laboratory confirmed mpox case on May 17, 2022 [7,26], being one of the most proportionally affected countries during the first epidemic wave (a total of 951 cases, as of January 2023) [16]. Vast genetic and epidemiological data, coupled with modelling approaches, allowed monitoring of the emergence, genetic diversity and spread of the virus, and estimated that a future epidemic wave could likely emerge as only a small proportion of the MSM population had been infected [16]. In June 2023, the beginning of a second epidemic wave was observed in Portugal, leading to a total of 229 confirmed cases until March 2024. Since the beginning of vaccine availability in Portugal (July 16, 2022), 9142 people have been vaccinated, as of May 31, 2024 [27]. The vaccination strategy foresees the use of the vaccine in groups at increased risk of human infection by the MPXV, with most inoculations (14954/16253; 92%) occurring in a pre-exposure context, as of May 31, 2024 [27,28].

In the present study, we took advantage of a large sequence sampling and vast epidemiological data to analyse the genetic variability of MPXV circulating in 2023. We employed a mathematical model to describe the transmission dynamics in this second epidemic wave. Our goal was to gain insights into the contribution of epidemiologically relevant groups, namely vaccinated/unvaccinated and low/high sexual activity, towards transmission while forecasting public health scenarios for future epidemic waves.

## Methods

### Study population

The National Institute of Health Doutor Ricardo Jorge (INSA) is the national reference laboratory. It is the Portuguese laboratory designated by the General Directorate of Health (through technical orientation no. 004/2022 of 31 May 2022) to process the samples for identification and characterization of MPXV [29].

All samples collected from patients with suspected mpox disease based on clinical observation in healthcare facilities nationwide, were sent to the Emergency Response and Biopreparedness Unit at INSA for MPXV screening. A confirmed mpox case was defined as a patient with clinically suspected mpox and PCR-confirmed infection, in a recommended specimen [30]. From June 14, 2023 (sample collection date of the first case confirmed of the second outbreak) until September 19, 2023, 543 samples from 299 probable cases were processed. Of these cases, 126 were confirmed mpox cases. The present study includes 121 confirmed cases in which laboratory and epidemiological investigations information was available.

### DNA extraction and MPXV laboratory diagnosis

According to the manufacturer’s recommendations, DNA extraction from clinical samples was performed using the ANDiS Viral RNA Auto Extraction & Purification Kit in an ANDiS 350 Automated Nucleic Acid Extraction System (3DMed). Real-time PCR diagnosis was based on an in-house Monkeypox virus-specific identification (B7R gene) protocol as previously described [31]. Positive, negative and internal (RNAse P gene) controls were included in all runs. All assays were performed on CFX Opus Real-Time PCR System (Bio-Rad). Results were given as cycle threshold (Ct) values, allowing estimation of viral load: MPXV detection was categorised as negative (Ct ≥ 40), weakly positive (35 ≤ Ct < 40), or positive (Ct < 35).

### Genomic sequencing and phylogenetic

DNA extracted from MPXV-positive samples was processed and sequenced as described previously [16], using an amplicon-based scheme [32]. Consensus sequences were obtained by analysing the sequencing reads in INSaFLU (https://insaflu.insa.pt/) [33], as previously described [16], but with an extra step of primer filtering using iVar [34] after genome alignment, as currently implemented in INSaFLU (v2.0) [35]. A consensus was considered for further analysis if more than 90% of the genome was covered at more than 10x of coverage. MPXV sublineage classification followed the international nomenclature proposed in https://github.com/mpxv-lineages [5]. A list of all the consensus sequences generated, and their accession identifiers, is available in Additional file 1. The acknowledgement table for the GIDsequences downloaded from GISAID that were used in the present study is provided in Additional file 2.

A global IQ-TREE [36] phylogenetic tree including all Portuguese consensus sequences from the 2022 outbreak, the sequences reported in this study, and publicly available international sequences of the B.1.3/C.1/C.1.1 sublineages with collection date within 2023 (as of 2023-12-14), was built using the Nextstrain [37] hMPXV-1 module available in INSaFLU (https://github.com/INSaFLU/nextstrain_builds/tree/main/mpx) [35]. Briefly, consensus sequences were aligned by nextalign against reference MPXV-M5312_HM12_Rivers (https://www.ncbi.nlm.nih.gov/nuccore/NC_063383), masking several regions of the genome, including the first 800 and last 6422 base pairs, as well as several repetitive regions of variable length, followed by phylogenetic reconstruction using IQTREE and ancestral state reconstruction and temporal inference using TreeTime [38]. Lastly, hMPXV1 lineages are assigned using markers specified here (https://github.com/INSaFLU/nextstrain_builds/blob/main/mpx/config/clades.tsv). The Nextstrain trees can be interactively explored in https://auspice.us/ using the JSON file and metadata provided as Additional file 3.

### Collection of epidemiological data and determination of Mpox Severity Score (Mpox-SS)

Each contributing healthcare facility was provided with a completed structured case report spreadsheet (CRS), with reporting variables adapted from other studies to capture data from medical records uniformly [11,16,39,40]. This CRS focused on demographic characteristics, potential exposures, vaccination, clinical findings, diagnosis and markers of disease severity. As described elsewhere, the Mpox Severity Score (Mpox-SS) was calculated according to the Mpox Severity Score System [39]. A Wilcoxon rank-sum test was used to evaluate differences between Mpox-SS of vaccinated (preventive vaccination related to this outbreak) and unvaccinated patients (cases of unknown vaccination status and other contexts of vaccination were not considered).

### Modelling transmission dynamics

We developed a compartmental susceptible-exposed-infectious-recovered (SEIR) model for the transmission of mpox in the MSM population. Full methodological details are presented in the Additional file 4. MSM individuals were characterised concerning two discrete heterogeneous states: vaccination and sexual activity. Each of these states has two categories: vaccinated or unvaccinated, and low or high sexual activity, respectively [21]. Individuals were considered to have high sexual activity if they reported more than 30 non-steady partners in the last 12 months. We also accounted for whether sexual contacts involved steady and non-steady partners. Sexual activity and steady-partner data applied in the model were based on information extracted from the European Men Who Have Sex With Men Internet Survey (EMIS) [41] conducted in 2017. Weekly absolute incidence and the proportion of high sexual activity and vaccinated cases were used to calibrate the model.

The reproduction number (Rt) was calculated for the duration of the 2023 wave (Additional file 4). Moreover, cumulative elasticities were computed, measuring the proportional contribution of each group toward transmission [22]. We also developed three possible scenarios for the weekly number of mpox cases after week 20, 2024 (2024-05-18):

- Scenario 1: five latent mpox imports with high sexual activity are introduced in the population;
- Scenario 2: five latent mpox imports with high sexual activity +1% of the unvaccinated and low sexual activity MSM population change to unvaccinated with high sexual activity;
- Scenario 3: five latent mpox imports with high sexual activity +2% of the unvaccinated and low sexual activity MSM population change to unvaccinated with high sexual activity.

The period considered for the projections was May 22, 2024, to October 9, 2024. The model was calibrated using Bayesian techniques. Additional file 4 provides a full description of the data used and the calibration procedure.

### Ethics and regulatory approval

This research complies with all relevant ethical regulations. The planning, development and reporting of this study was in accordance with the Declaration of Helsinki, as revised in 2013. All samples were processed exclusively for laboratory diagnosis of MPXV, as requested by the clinicians (co-authors of the present study), and for viral genetic characterization at the National Institute of Health Doutor Ricardo Jorge (INSA), as designated by the General Directorate of Health through the technical orientation no. 004/2022 of 31 May 2022. For the purpose of the present study, all data was processed in a pseudonymized form, with the clinicians participating in the study being the ones with exclusive access to patient records to extract the epidemiological data. This study was approved by the Ethical Committee for Health of INSA (Approval N° 149/2024).

## Results

### Demographic, epidemiological and clinical data

A sample case series (*n* = 121) was studied to describe epidemiological and clinical features of mpox cases from this second outbreak, between June 14, 2023 and September 19, 2023. Patients were aged between 20 and 55 years old (median age 32 years), being mostly in the age brackets of 30–39 years (48/121; 39.7%) and 20–29 years (46/121; 38.0%). Most individuals were male (119/121; 98.3%) and self-identified as MSM (118/120; 98.3%). There were only two cases (2/120; 1.7%) in females, both from the 20–29 age group (Table 1).

**Table 1.** Demographics, sexual history, vaccination status and clinical characteristics of the cases.

	Case counts and proportion among the total number of infected individuals (*n* = 121)	Case counts and proportions among the infected individuals with information available for each variable
**Age**	median 32 (range 20–55) years	
**Sex assigned at birth**		
Male	119/121 (98.3%)	
Female	2/121 (1.7%)	
**Sexual self-identification**		
MSM	118/121 (97.5%)	118/120 (98.3%)
Other	2/121 (1.7%)	2/120 (1.7%)
Unknown	1/121 (0.8%)	
**Sexual partners in the past 30 days**		
Two or more male partners	82/121 (67.8%)	82/114 (71.9%)
One male partner	18/121 (14.9%)	18/114 (15.8%)
Two or more male and female partners	11/121 (9.1%)	11/114 (9.6%)
No partner	3/121 (2.5%)	3/114 (2.6%)
Do not know / Do not answer	7/121 (5.8%)	
**Type of sex in the past 30 days**		
Oral and anal	98/121 (81.0%)	98/110 (89.1%)
Anal only	9/121 (7.4%)	9/110 (8.2%)
Oral only	3/121 (2.5%)	3/110 (2.7%)
Unknown	11/121 (9.1%)	
**Condomless sex in the past 30 days**		
Yes	105/121 (86.8%)	105/113 (92.9%)
No	8/121 (6.6%)	8/113 (7.1%)
Unknown	8/121 (6.6%)	
**Sexualized drug use**		
Yes	17/121 (14.0%)	17/71 (23.9%)
No	54/121 (44.6%)	54/71 (76.1%)
Unknown	50/121 (41.3%)	
**The most likely context of exposure**		
Household	36/121 (29.8%)	36/81 (44.4%)
Sexual contact in a nightclub/bar/private event/sauna or similar	36/121 (29.8%)	36/81 (44.4%)
Large events with sexual contacts	5/121 (4.1%)	5/81 (6.2%)
Small events without sexual contacts	3/121 (2.5%)	3/81 (3.7%)
Large events without sexual contacts	1/121 (0.8%)	1/81 (1.2%)
Unknown	40/121 (33.1%)	
**The most likely context of exposure was sauna**		
Yes	21/121 (17.4%)	21/83 (25.3%)
No	62/121 (51.2%)	62/83 (74.7%)
Unknown	38/121 (31.4%)	
**Most likely route of transmission**		
Sexual	106/121 (87.6%)	106/119 (89.1%)
Person-to-person (excludes mother-child, associated with health and sexual care)	13/121 (10.7%)	13/119 (10.9%)
Unknown	2/121 (1.7%)	
**Travel history**		
Yes	3/121 (2.5%)	3/94 (3.2%)
No	91/121 (75.2%)	91/94 (96.8%)
Unknown	27/121 (22.3%)	
**Sexual relations in PT with foreign tourists**		
Yes	8/121 (6.6%)	8/46 (17.4%)
No	38/121 (31.4%)	38/46 (82.6%)
Unknown	75/121 (62.0%)	
**Sexual intercourse with an anonymous sex partner or multiple sex partners**		
Yes	91/121 (75.2%)	91/110 (82.7%)
No	19/121 (15.7%)	19/110 (17.3%)
Unknown	11/121 (9.1%)	
**HIV status**		
Positive	52/121 (43.0%)	52/120 (43.3%)
Negative	68/121 (56.2%)	68/120 (56.7%)
Unknown	1/121 (0.8%)	
**Last CD4 Count of people living with HIV at the time of mpox diagnosis**		
High (≥500 cells/mm^3^)	26/52 (50.0%)	26/36 (72.2%)
Low (<500 cells/mm^3^)	10/52 (19.2%)	10/52 (27.8%)
Unknown	16/52 (30.8%)	
**PrEP use in HIV negative patients**		
Yes	24/68 (35.3%)	24/68 (35.3%)
No	43/68 (63.2%)	43/68 (64.2%)
Unknown	1/68 (1.5%)	
**Number of STI (excluding HIV) present at time of mpox diagnosis**		
0	52/121 (43.0%)	52/88 (59.1%)
1	28/121 (23.1%)	28/88 (31.8%)
≥2	8/121 (6.6%)	8/88 (9.1%)
Unknown	33/121 (27.3%)	
**Smallpox vaccination**		
Yes	17/121 (14.0%)	17/111 (15.3%)
No	94/121 (77.7%)	94/111 (84.7%)
Unknown	10/121 (8.3%)	
**Smallpox vaccination context**		
Preventive (related to the current outbreak)	15/17 (88.2%)	15/17 (88.2%)
Preventive (childhood)	1/17 (5.9%)	1/17 (5.9%)
Post-exposure	1/17 (5.9%)	1/17 (5.9%)
Number mpox lesions*	median 6 (range 1–50) lesions	
0	3/121 (2.5%)	3/110 (2.7%)
<5	37/121 (30.6%)	37/110 (33.6%)
5–9	21/121 (17.4%)	21/110 (19.1%)
10–14	30/121 (24.8%)	30/110 (24.8%)
15–19	7/121 (5.8%)	7/110 (6.4%)
≥20	12/121 (9.9%)	12/110 (10.9%)
Unknown	11/121 (9.1%)	
**Duration of rash**	median 13 (range 1–31) days	
Known	104/121 (86.0%)	
Unknown	17/121 (14.0%)	
Lesions with a diameter ≥2cm*		
Yes	17/121 (14.0%)	17/93 (18.3%)
No	76/121 (62.8%)	76/93 (81.7%)
Unknown	28/121 (14.0%)	
Lesion location*		
Genital mucosal lesion	67/121 (55.4%)	67/107 (62.6%)
Anal mucosal lesion	50/121 (41.3%)	50/100 (50.0%)
Groin/Buttocks/Perianal lesion	51/121 (42.1%)	51/99 (51.5%)
Oropharyngeal lesion	18/121 (14.9%)	18/91 (19.8%)
Ocular lesion	1/121 (0.8%)	1/91 (1.1%)
Head/Neck lesion	41/121 (33.9%)	41/102 (40.2%)
Chest/Abdomen lesion	42/121 (34.7%)	42/91 (46.2%)
Back lesion	39/121 (32.2%)	39/94 (41.5%)
Left arm lesion	19/121 (15.7%)	19/89 (21.3%)
Left foot lesion	10/121 (8.3%)	10/86 (11.6%)
Left leg lesion	16/121 (13.2%)	16/86 (18.6%)
Left hand lesion	19/121 (15.7%)	19/89 (21.3%)
Right arm lesion	24/121 (19.8%)	24/90 (26.7%)
Right foot lesion	9/121 (7.9%)	9/86 (10.5%)
Right leg lesion	20/121 (16.5%)	20/86 (23.3%)
Right hand lesion	17/121 (14.0%)	17/87 (19.5%)
**Exanthem**		
Yes	47/121 (38.8%)	47/118 (39.8%)
No	71/121 (58.7%)	71/118 (60.2%)
Unknown	3/121 (2.5%)	
**Fever**		
Yes	75/121 (62.0%)	75/117 (64.1%)
No	42/121 (34.7%)	42/117 (35.9%)
Unknown	4/121 (3.3%)	
**Fever intensity**		
High (≥38°)	27/79 (34.2%)	27/71 (38.0%)
Low (<38°)	44/79 (55.7%)	44/71 (62.0%)
Unknown	8/79 (10.1%)	
**Duration of fever**	median 3 (range 1–31) days	
Treatment for bacterial superinfection*		
Yes	18/121 (14.9%)	18/117 (15.4%)
No	99/121 (81.8%)	99/117 (84.6%)
Unknown	4/121 (3.3%)	
Level of care*		
Outpatient	112/121 (92.6%)	112/118 (94.9%)
Hospitalization (due to mpox) but without ICU	6/121 (5.0%)	6/118 (5.1%)
Unknown	3/121 (2.5%)	
Pain, analgesia requirement*		
Outpatient without analgesic prescription	55/121 (45.5%)	55/92 (59.8%)
Outpatient with analgesic prescription	31/121 (25.6%)	31/92 (33.7%)
Hospitalization with intravenous analgesics	6/121 (5.0%)	6/92 (6.5%)
Unknown	29/121 (24.0%)	
**The patient received antiviral treatment (Tecovirimat)**		
Yes	5/121 (4.1%)	5/110 (4.5%)
No	105/121 (86.8%)	105/110 (95.5%)
Unknown	11/121 (9.1%)	

Parameters used for Mpox-SS calculations are marked with an asterisk (*).

Among the cases with available epidemiological data (whose number varied depending on the metadata variable; Table 1 and Additional file 1), sexual contact was the most frequently reported transmission route (106/119; 89.1%). Most cases reported having sex with multiple male partners (82/114; 71.9%), oral and anal sex (98/110; 89.1%), and condomless sex (105/113; 92.9%) in the past 30 days. Some cases reported use of drugs in sexual practices (17/71; 23.9%). Sixty-five cases reported exposure 21 days prior to the onset of symptoms. Regarding the most likely context of exposure, most patients reported household (36/81; 44.4%) and sexual contact in a nightclub/bar/private event/sauna or similar (36/81; 44.4%). A few patients reported exposure in large events, with (5/81; 6.2%) or without (1/81; 1.2%) sexual contacts, and small events without sexual contacts (3/81; 3.7%). Most patients reported sexual intercourse with an anonymous sex partner or multiple sex partners (91/110; 82.7%).

Human immunodeficiency virus (HIV) infection status was reported as positive in 52 (52/120; 43.3%) of the patients. The last CD4 count was reported for 36 out of the 52 patients living with HIV, 26 (26/36; 72.2%) of which had high counts (≥500 cells/mm^3^). Of the 68 (68/120; 56.7%) patients who were HIV negative, 24 (24/68; 35.3%) were known to be on HIV pre-exposure prophylaxis (PrEP). Other concomitant sexually transmitted infections (STI) were also frequently reported, with 28 (28/88; 31.8%) cases documenting at least one STI (excluding HIV) at the time of mpox diagnosis.

Concerning smallpox vaccination, only 17 (17/111; 15.3%) patients were vaccinated. The vaccination context was 88.2% (15/17) preventive and related to the current outbreak, 5.9% (1/17) preventive in childhood (back to 1976) and 5.9% (1/17) post-exposure. The last two situations were cases in which only one dose of the vaccine was received. Noteworthy, one case of mpox reinfection was confirmed by viral sequencing (see below) in this outbreak after one year of the first infection (June, 2022 and August, 2023).

Most cases were outpatients (112/118; 94.9%), with the analgesic requirement being reported for 31 out of 92 (31/92; 33.7%) cases with available information. Hospitalization due to mpox was reported for six (6/92; 6.5%) patients, all receiving intravenous analgesics but without resorting to intensive care units (ICU). Two of the hospitalised patients received mpox-specific antiviral therapy (Tecovirimat).

Of the 107 (89.3%) cases with data available, the median number of lesions was six (range 1–50), with a considerable proportion of cases (47/107; 43.9%) reporting at least 10 lesions. The median duration of the rash was 13 days (range 1–31). The most commonly involved area was the genital mucosa (67/107; 62.6%), followed by groin/buttocks/perianal (51/99; 51.5%) and anal mucosal (50/100; 50.0%) areas.

Forty-seven (47/118; 39.8%) cases developed exanthema, and 75 (75/117; 64.1%) had a fever. The fever intensity was low (<38°C) for 44 (44/71; 62.0%) patients and high (≥38°) for 27 (27/71; 38.0%) patients. The median duration of fever was three days (range 1–31).

**Figure 1.**
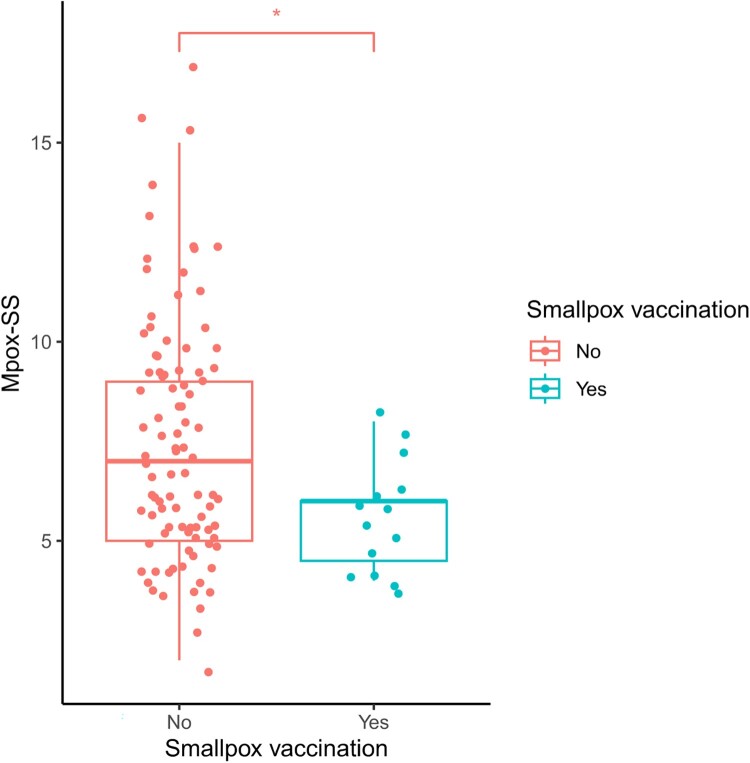
Vaccinated individuals present lower MPox Severity Score (Mpox-SS) values than unvaccinated individuals. Mpox-SS evaluation between vaccinated and unvaccinated patients using the Wilcoxon rank-sum test. Results are presented as box and whisker plots, with median and interquartile ranges represented in boxes and ranges presented as whiskers. The asterisk (*) indicates that the *p*-value of the statistical test is less than the significance level of 0.05.

Based on the calculation of a Mpox-SS, as described elsewhere [39], disease severity tended to be lower (*p* = 0.02) in patients who had taken preventive vaccination (median = 6.0, IQR = 4.5–6.0, *n* = 15) than in unvaccinated patients (median = 7.0, IQR = 5.0–9.0, *n* = 94) (Figure 1). Of note, all vaccinated cases presented Mpox-SS less than or equal to eight, while a considerable proportion of non-vaccinated cases (42/94; 44.7%) presented Mpox-SS between 8 and 17.

### Viral phylogenomic data

We could obtain consensus sequences for 76 samples subjected to viral genome sequencing (Additional file 1). As expected, samples for which we could not obtain a consensus tended (*p* < 0.001) to have higher CT values (median CT = 22, IQR = 19–25, *n* = 21) than samples for which we could obtain consensus (median CT = 18, IQR = 17–21, *n* = 76). The CT values among the studied cases (*n* = 121) ranged from 11–36, with a median of 20.

**Figure 2.**
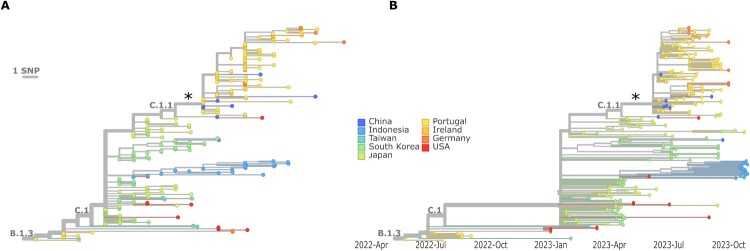
The second mpox wave in Portugal was driven by MPXV from lineage C.1.1. A. Nextstrain phylogenetic tree (branch lengths as a measure of nucleotide divergence) enrolling MPXV sequences collected in Portugal during the study period (June 14, 2023, and September 19, 2023), sequences from the 2022 outbreak in Portugal of the B.1.3 lineage, as well as publicly available international sequences from the B.1.3, C.1 and C.1.1 lineages collected within 2023 (as of December 2023). B. Time-scaled Nextstrain phylogenetic tree using the same dataset as in panel A. Asterisk (*) marks the C.1.1 sub-cluster (defined by the G152866A mutation, concerning MPXV-M5312_HM12_Rivers genome; NC_063383.1) enrolling all Portuguese sequences from the second mpox wave. Node colors represent different countries.

All of the viral genome sequences identified in the study period of the second mpox wave in Portugal belong to the C.1.1 sublineage (descendent of B.1.3 and C.1) (Figure 2; Additional file 1) and form a well-defined sub-cluster defined by the G152866A mutation (position refers to MPXV-M5312_HM12_Rivers genome; NC_063383.1). Upon integrating these 76 sequences with publicly available international sequences, we observed that the ancestral C.1 sublineage had circulated in several Asian countries since at least January 2023 (Figure 2B; Additional file 3). In particular, a few C.1.1 sequences collected in China between June and July 2023 clustered together with sequences from Portugal, including two sequences genetically identical to Portuguese sequences placed at the root of the second outbreak in Portugal (Figure 2A; Additional file 3).

Sequencing data also confirmed the suspected reinfection case detected in the second mpox wave. This individual was infected with MPXV from the C.1.1 sublineage (August, 2023) more than one year after the first infection (June, 2022) with MPXV from lineage B.1, discarding the scenario of long-term persistent infection (Additional file 5). No further investigation could be carried out related to this reinfection, due to the unavailability of data regarding immune response and immunological status.

### Impact of vaccination status and sexual activity on transmission dynamics

**Figure 3.**
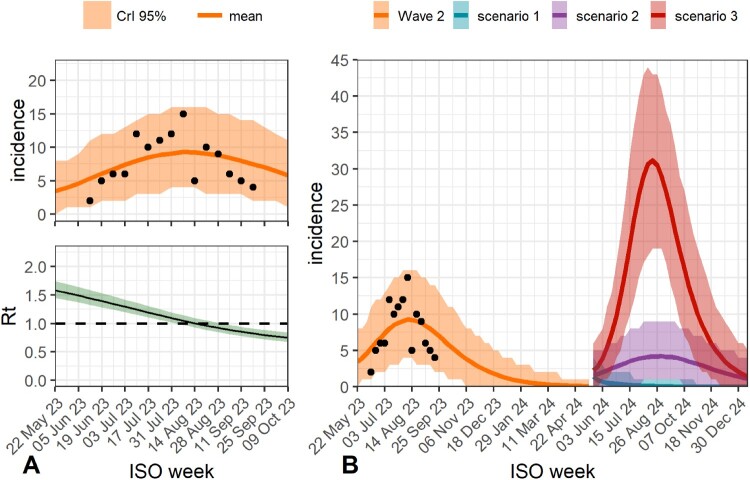
Transmission modelling of the second mpox wave in Portugal and forecasting public health scenarios through a compartmental susceptible-exposed-infectious-recovered (SEIR) model. The model was fitted using Bayesian techniques. A (top). Describes the model fit to the observed data of weekly incident cases (as represented by the black dots). The fit is presented by the mean trajectory of the number of infections along with the 2.5% and 97.5% percentiles (95% CrI). A (bottom). Presents the median evolution of the reproduction number along with 95% CrI. The reproduction number was computed via the next generation method. B. presents the weekly-modelled incidence for the second wave along with the associated trajectories for the possible epidemic scenarios for the summer of 2024 posed in the methods section.

To understand the transmission of mpox during the second wave in Portugal, we used a compartmental SEIR model focused on vaccination status and sexual activity, as detailed in Additional file 4. We further evaluated the probable impact of such variables for subsequent mpox outbreaks. Figure 3A depicts the model fit to the observed data, supporting that the applied model can explain the 2023 mpox wave. We found a reproduction number of 1.58 [95% Credible Interval (95%CrI): 1.45–1.75] at the end of May 2023, indicating that an infected individual would, on average infect approximately more than one individual at this stage. As the outbreak progressed, the reproduction number descended below one at the beginning of August 2023, coinciding with the downturn in the number of weekly infections, until reaching 0.75 (95% CrI: 0.67–0.84) by early October 2023.

By employing the innovative approach of elasticity indicators [42], we were able to estimate that the high sexual activity group’s contribution to transmission during early summer 2023 was 120.57 (95% CrI: 30.72–3553.56) times larger than the low sexual activity group, indicating extremely high heterogeneity in transmission between the two groups. Moreover, comparing the contributions of the vaccinated high sexual activity group with the unvaccinated high sexual activity group, we estimated that the former contributes approximately eight times less towards the Rt: [0.123 (95% CrI: 0.068–0.208)]. We assume a reporting rate of 0.62 for this second wave, equal to that estimated for the first wave [16]. A sensitivity analysis was performed by assuming a decline in the reporting rates of mpox (0.31 instead of 0.62) (Additional file 4). The Rt during the early phase of the outbreak is estimated to be marginally higher in the former compared to the latter. The remaining results are in agreement between both assumptions (Additional file 4).

### Forecasting public health scenarios for a future epidemic wave

Given the previous results on high sexual activity playing a major role in viral spread, we conducted model projections of epidemic potential in different scenarios for the summer of 2024. In the first scenario, we explored the possibility of an outbreak in which five infected cases with high sexual activity would be introduced in the population after week 20 of 2024 (scenario 1). We estimated that this scenario would not result in an epidemic wave. We also explored other scenarios, where on top of the five imports we also assume that 1% (scenario 2) and 2% (scenario 3) of the MSM population with low sexual activity would increase their sexual contacts matching those of the high sexual activity group [15,43]. We forecasted a new epidemic wave during the summer of 2024 in both scenarios, with an eventual phase-out by October 2024 as observed during the 2023 wave. Noteworthy, these results show that the magnitude of the wave depends on the change in sexual activity, with higher variations in the high sexual activity population resulting in a wave with a higher peak of infections. We note that the simulations refer to the spread of MPXV from clade IIb that caused the 2022–2023 worldwide outbreak. As such, they did not account for the possibility of emergence and co-circulation of other variants, namely from clade I.

## Discussion

Portugal was one of the most affected countries during the 2022 mpox outbreak, likely playing an important role in the early and widespread dissemination of MPXV worldwide [16]. After a few months with no reported cases, a second epidemic wave was observed in summer 2023, contrasting with the most European countries where only steady basal levels of infection were observed [3]. In the present study, similarly to the 2022 outbreak, we took advantage of a large sequence sampling (63%) and the C.1.1 sublineage caused the 2023 outbreak in Portugal. The extremely low number of MPXV sequences released by most European countries hampers the accurate assessment of the relative proportion of cases caused by C.1 / C.1.1 sublineage in Europe. Indeed, only Germany also reported tenths of sequences from samples collected in 2023 (based on available sequence data in GenBank and GISAID, as of July 5, 2024), with B.1.22 being the most reported lineage, despite the co-circulation of C.1/C.1.1 and B.1.20. Of note, B.1.20 was the most reported lineage by the USA in summer 2023. In contrast, several countries from the Western Pacific Region, such as China, South Korea and Japan, experienced MPXV outbreaks in 2023 [3] marked by a clear dominance of C.1/C.1.1 sublineage, which represented almost all released sequences in 2023 from these countries (as of 5 July 2024, in GISAID and GenBank). Our phylogenetic analysis of C.1.1 sequences reported globally in 2023 suggests that the second mpox wave in Portugal resulted from limited introduction(s) of C.1.1 lineage in Portugal, likely from Asia, followed by sustained transmission. The active circulation of C.1 in this region supports the hypothesis of viral introduction from Asia at least since January 2023, as well as by the detection of two C.1.1 sequences from China genetically identical to Portuguese sequences placed at the root of the second outbreak in Portugal. The exportation of the C.1.1 lineage from Portugal to other countries, namely Germany and Ireland, might have also occurred, as the “Portuguese” sub-cluster also integrates sequences from mpox cases detected in these countries later on (August-October, 2023). However, these hypotheses could not be confirmed due to the scarce availability of contact tracing data and few data on travel history. These public health challenges were also observed during the 2022 MPXV outbreak investigation [16] and could not be overcome in the 2023 wave. This is most likely due to the complex nature of the global MPXV outbreak, which is marked by international sexual networks and a considerable proportion of cases reporting sexual contact with multiple or anonymous individuals, as previously discussed [16].

Although the 2023 mpox wave was smaller than the 2022 outbreak, both epidemics occurred in the summer months (likely due to increased tourism and festivals) [15] and had similar clinical end epidemiological properties, namely regarding demographic variables and age, sexual self-identification, HIV infection cases status and route of transmission by sexual contact. Different possible reasons may explain the smaller number of cases, such as increased immunity provided from vaccination against mpox or previous infections and the impact of control intervention by public health authorities leading to behaviour change in MSM communities [25]. For instance, a notable change was observed in the exposure context, for which reports of household exposure almost duplicated from the first to the second wave (26.4% and 44.4%, respectively).

To understand better the transmission dynamics and the main drivers of the second epidemic wave, we estimated the role of sexual activity towards transmission by employing a SEIR model that accounts for the vaccination status while forecasting public health scenarios for future epidemic waves (Additional file 4). We estimated that the contribution to transmission of the high sexual activity group was approximately 120-fold higher than that of the low sexual activity group. Moreover, the scenario analysis projected that mpox transmission is highly sensitive to sexual behaviour, indicating that a slight increase in the population of MSM individuals with high sexual activity may result in a new mpox epidemic wave in 2024 or epidemics with very different magnitudes (Figure 3B). This data corroborates the need to raise awareness by reinforcing public health messages targeting this MSM high-risk group, including the involvement of stakeholders associated with the MSM community. Still, our results also strongly suggest that vaccination efforts should be maintained due to their critical role in controlling transmission by smoothing the impact of high-risk sexual activity. Indeed, we estimated that the vaccinated high sexual activity group contributes approximately eight times less towards the Rt than the unvaccinated high sexual activity group [44].

Contrary to the first wave, in which the effect of vaccination could only act when cases were already declining (most likely due to changes in sexual behaviour [16,20,21], as also observed in other countries [19,25]), the reduction in Rt observed during the second wave in Portugal should be mostly attributable to vaccination [23] and infection-induced immunity [22]. Another relevant observation was that vaccination was found to be associated with reduced severity. The vaccinated group presented an Mpox-SS of 6.0 [IQR 4.5–6.0], whereas the score estimated for unvaccinated patients was 7.0 [IQR 5.0–9.0] (Figure 1). These estimates are in agreement with a previous study showing that past infection or vaccination nearly equally reduced severity, with Mpox-SS of 7.0 [IQR 7–10], 5.5 [IQR 4–7], and 5.0 [IQR 3–7] for first infection, re-infection, and vaccinated groups, respectively [39]. Despite these indicators, further research is needed to better understand vaccine effectiveness in preventing infection, as previously noticed [39]. Also, our studied population enrolled just one case with post-exposure vaccination, hampering any inference about the role of this pharmaceutical intervention in preventing disease.

Overall, although the present study could gather vast genomic and epidemiological data to better understand the second mpox outbreak in Portugal, a few lessons could be learned that are relevant for future prevention and control of this disease. Besides some limitations discussed above (e.g. scarce availability of international MPXV sequence data, contact tracing data and travel history information), we highlight that a major source for data on sexual health indicators in MSM, the EMIS report [41], refers to 2017, which might not fully reflect the current situation. EMIS was an essential tool for mpox transmission modelling and forecast, thus more frequent updates would be advisable. Moreover, a better convergence between the variables captured by this large European survey and the medical inquiries in place across different countries would be beneficial for a harmonised epidemiological data collection and outbreak investigation. This could ensure a more robust identification of variables found to be key to explaining mpox transmission in the MSM population. For instance, EMIS reports statistics on a 12-month period, which brings challenges when translating the estimates (e.g. number of non-steady sexual partners) to the <1 month MPXV incubation period (i.e. the focus of the clinical questionnaire) and the exposure context. As the outcomes of MPXV infection become more predictable and likely less severe due to acquired immunity, we anticipate that the difficulty in detecting the infected individuals may increase, creating more obstacles for transmission monitoring and control while challenging the identification of new MPXV introductions. The latter might be of particular concern considering the potential global expansion of the more severe MPXV from clade I, which is in active circulation in the Democratic Republic of Congo [9,10,45], with reports of transmission through sexual contact [46]. This potential global threat further emphasises the need for the countries to increase the MPXV sequencing efforts, while keeping high levels of awareness among health professionals, MSM and the general population about an “old” virus with a “new” ecology, evolutionary dynamics and global epidemiology.

## Supplementary Material

Additional file 5.pdf

Additional file 1.xlsx

Additional file 4.pdf

Additional file 2.pdf

Additional file 3.zip

## Data Availability

The data supporting the findings of this study are available within the paper and its Supplementary materials. MPXV reads and consensus sequences are available for downloading through the accession number provided in Additional file 1. Software code used in the mathematical modelling is available upon reasonable request.
