# Supplementary material for: Viral genetics and transmission dynamics in the second wave of mpox outbreak in Portugal and forecasting public health scenarios
Source: Emerg Microbes Infect. 2024 Oct 3;13(1):2412635. doi: 10.1080/22221751.2024.2412635 (PMC11486115; doi:10.1080/22221751.2024.2412635)
Supplement: Additional file 5.pdf [file TEMI_A_2412635_SM0280.pdf]

Additional file 5

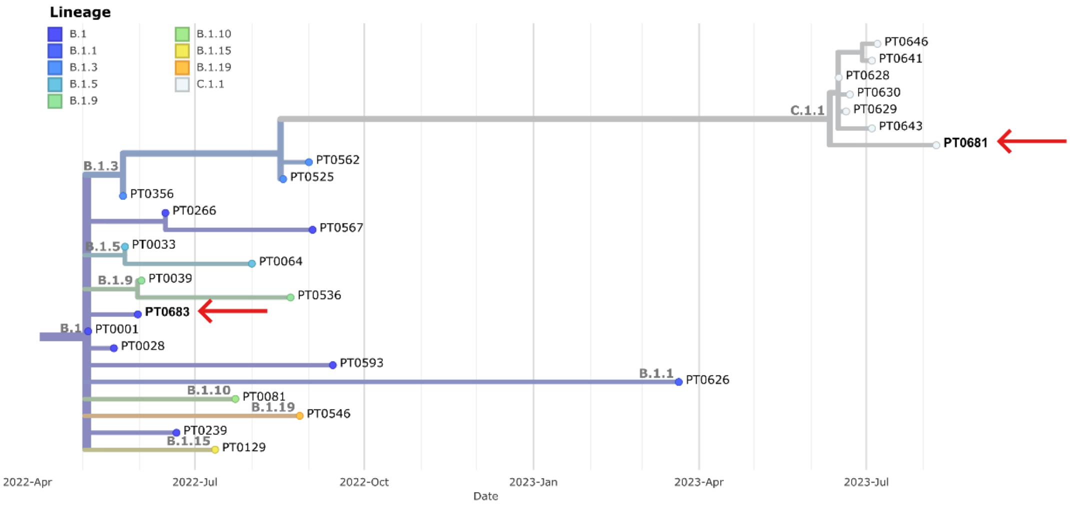

MPXV genome sequencing provides strong evidence for a case of reinfection. Time-scaled Nextstrain phylogenetic tree showing the two samples (marked with red arrows) of the same individual separated by more than a year. To better frame the genetic distance between the two samples of interest, we included a subset of all the Portuguese samples that are broadly representative of the observed genetic diversity. Node colors represent different lineages.
