# Supplementary material for: Viral genetics and transmission dynamics in the second wave of mpox outbreak in Portugal and forecasting public health scenarios
Source: Emerg Microbes Infect. 2024 Oct 3;13(1):2412635. doi: 10.1080/22221751.2024.2412635 (PMC11486115; doi:10.1080/22221751.2024.2412635)
Supplement: Additional file 4.pdf [file TEMI_A_2412635_SM0278.pdf]

Viral genetics and transmission dynamics in the second wave of mpox outbreak in Portugal and forecasting public health scenarios - Additional file 4

1 Introduction

This document contains the description of the compartmental model and data used in the analysis of the second wave of mpox in Portugal during 2023.

2 Transmission model

We developed a compartmental susceptible-exposed-infectious-recovered (SEIR) model for the transmission of mpox in the men who have sex with men (MSM) population. The model architecture was based in previous mpox modelling works [19]. A diagram of the model is presented below.

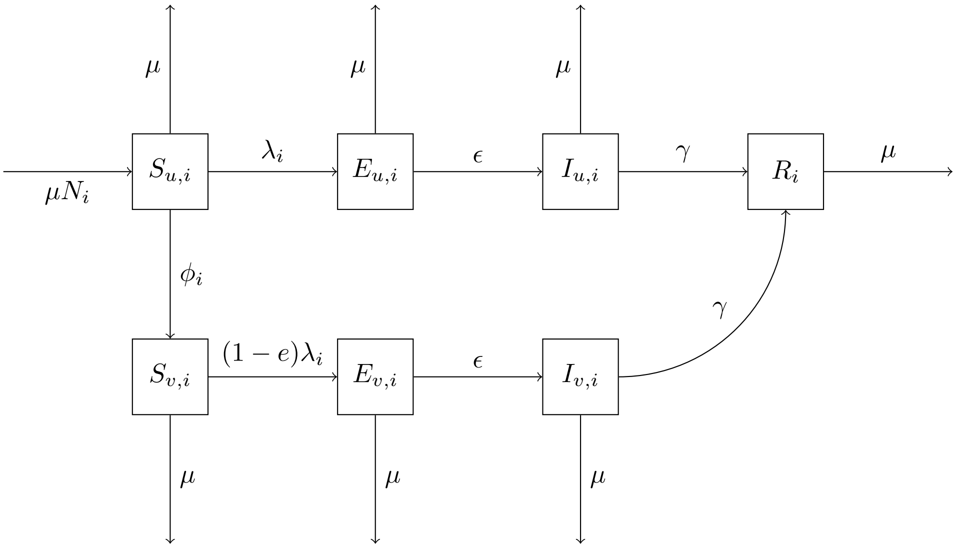

Figure 1: Diagram of the mpox SEIR model.

Vaccination for mpox is ongoing since July 2022. Moreover, individuals born prior to 1976 were eligible to receive the smallpox vaccine, which still confers a degree of protection against mpox [11, 8, 14]. We also took into account

the heterogeneity in sexual contacts within the MSM population, given that mpox transmission can occur via sexual/intimate contacts [17]. To this end we classified MSM individuals concerning two discrete heterogeneous states: vaccination and sexual activity. Each of these states has two groups: vaccinated (subscript v) or unvaccinated (subscript u) and low sexual activity (subscript l) or high sexual activity (subscript h). We also accounted for sexual contacts between steady (subscript s) and non-steady (subscript n) partners.

For each heterogeneous state pair, individuals are categorised according to their disease state as either susceptible, exposed, infectious or recovered. Following the SEIR model structure, adapted from modelling works for mpox [19]. The inflows and outflows of the system are given by the ordinary differential equation system presented below:

$$\begin{cases} S'_{v,i} = -(1-e)\lambda_i S_{v,i} + \phi_i S_{u,i} - \mu S_{v,i} \\ E'_{v,i} = (1-e)\lambda_i S_{v,i} - (\epsilon + \mu)E_{v,i} \\ I'_{v,i} = \epsilon E_{v,i} - (\gamma + \mu)I_{v,i} \\ S'_{u,i} = -\lambda_i S_{u,i} - (\phi_i + \mu)S_{u,i} + \mu N_i \\ E'_{u,i} = \lambda_i S_{u,i} - (\epsilon + \mu)E_{u,i} \\ I'_{u,i} = \epsilon E_{u,i} - (\gamma + \mu)I_{u,i} \\ R'_i = \gamma(I_{v,i} + I_{u,i}) - \mu R_i. \end{cases} \tag{1}$$

Population is assumed to be constant

$$\sum_{i=h,l} S'_{v,i} + E'_{v,i} + I'_{v,i} + S'_{u,i} + E'_{u,i} + I'_{u,i} + R'_i = 0, \tag{2}$$

with

$$S_{v,i} + E_{v,i} + I_{v,i} + S_{u,i} + E_{u,i} + I_{u,i} + R_i = N_i, \tag{3}$$

where N_i is the number of MSM individuals in sexual activity group i, $i = h, l$.

Parameters e, ϵ, γ and μ, denote the vaccine effectiveness, rate out of the exposed compartment, recovery rate and population renewal rate. ϕ_i denotes the vaccinate rate in sexual activity group i, $(i = h, l)$. The force of infection is written as

$$\lambda_i = \lambda_{s,i} + \lambda_{n,i}, \ i = h, l \tag{4}$$

It is given as the sum of force of infection coming from sexual contacts with steady partners

$$\lambda_{s,i} = q_i \sum_{k=v,u} \sum_{j=h,l} \pi_{s,i,j} \big(1 - (1-\beta)^{c_{ij}}\big) \frac{I_{k,j}}{N_j}, \tag{5}$$

and with non-steady partners [19]

$$\lambda_{n,i} = \alpha_{n,i} \sum_{k=v,u} \sum_{j=h,l} \pi_{n,i,j} \beta \frac{I_{k,j}}{N_j}. \tag{6}$$

Where β denotes the probability of transmission given a sexual contact and c_{ij} denotes the rate of sexual contacts, among steady partners, an individual of sexual activity group i has with a partner of sexual activity group j, $(i, j = h, l)$.

We assume one sexual encounter per non-steady partner. $\alpha_{n,i}$ denotes the rate of acquisition of non-steady partners of an individual in sexual activity group i, $(i = h, l)$. $\pi_{s,i,j}$ and $\pi_{n,i,j}$ define the mixing between each sexual activity group [19]:

$$\pi_{k,i,j} = \theta_k \delta_{i,j} + (1 - \theta_k) \frac{\alpha_{k,j} N_j}{\sum_{m=h,l} \alpha_{k,m} N_m}, \; k = s, n, \; i, j = h, l, \tag{7}$$

where δ_{ij} represents the Kronecker delta function (1 if $i = j$, 0 otherwise) and θ_k represents the assortativeness of the mixing between high sexual activity individuals and low sexual activity individuals in the setting of $k = s$ (steady partners) or $k = n$ (non-steady partners). If $\theta_k = 1$ then individuals only engage in sexual activity with individuals in the same activity group, if $\theta_k = 0$ then the mix is proportionate [19].

2.1 Reproduction number

The next generation matrix [5, 6] is obtained via the linearization of the subsystem of infected. It is given by

$$\mathbf{K} = -\mathbf{T}\mathbf{\Sigma}^{-1},$$

where $\mathbf{T}$ corresponds to the matrix with rates of new infections and $\mathbf{\Sigma}$ the rates out of and between infected states [5]. Its form for model (1) is as follows

$$\mathbf{K} = \xi \begin{bmatrix} (1-e)S^*_{v,h}X_{h,h} & (1-e)S^*_{v,h}X_{h,h} & (1-e)S^*_{v,h}X_{h,l} & (1-e)S^*_{v,h}X_{h,l} \\ S^*_{u,h}X_{h,h} & S^*_{u,h}X_{h,h} & S^*_{u,h}X_{h,l} & S^*_{u,h}X_{h,l} \\ (1-e)S^*_{v,l}X_{l,h} & (1-e)S^*_{v,l}X_{l,h} & (1-e)S^*_{v,l}X_{l,l} & (1-e)S^*_{v,l}X_{l,l} \\ S^*_{u,l}X_{l,h} & S^*_{u,l}X_{l,h} & S^*_{u,l}X_{l,l} & S^*_{u,l}X_{l,l} \end{bmatrix} \tag{8}$$

where $\xi = \frac{\epsilon}{(\epsilon+\mu)(\gamma+\mu)}$ and

$$X_{i,j} = q_i\big(\pi_{s,i,j}\big(1 - (1-\beta)^{c_{ij}}\big)\frac{1}{N_j}\big) + \alpha_{n,i}\pi_{n,i,j}\beta\frac{1}{N_j}, \; i, j = h, l. \tag{9}$$

The reproduction number R_t is the spectral radius of $\mathbf{K}$ around a specific vector of susceptibility $\mathbf{S}^* = (S^*_{v,h}, S^*_{u,h}, S^*_{v,l}, S^*_{u,l})$ [2]. Cumulative elasticities are given by the following formula [2]:

$$e_j = \sum_{i=1}^{4} \frac{k_{ij}}{Rt} \frac{\partial Rt}{\partial k_{ij}}, \tag{10}$$

where k_{ij} are entries of $\mathbf{K}$ and $\frac{\partial Rt}{\partial k_{ij}} = v_i w_j$. With v_i and w_j corresponding to the entries of the dominant left eigenvector ($\mathbf{v}$) and dominant right eigenvector ($\mathbf{w}$) of $\mathbf{K}$, respectively. Vectors $\mathbf{v}$ and $\mathbf{w}$ must satisfy $\langle \mathbf{v}, \mathbf{w} \rangle = 1$ [2].

3 Data

3.1 mpox case data

Case data for mpox was obtained for 118 MSM individuals. Dates of sample collection span from 2023-06-14 to 2023-09-19. These are categorised in high

and low sexual activity according to the number of partners reported in the last 30 days and also if they had anonymous sex or sex with multiple partners in the past 30 days. Individuals with more than one sexual partner and/or anonymous sex with multiple partners are considered to belong to the high sexual activity group. Cases with only one sexual partner and without anonymous sex with multiple partners are considered to belong to the low sexual activity group. Approximately 89% of cases belong to the high activity group. Moreover, 15% of the cases had received the mpox vaccine, with only 1 individual having receiving it as a child in 1976. Weekly number of mpox cases, using the date of sample collection, along with the proportion of high sexual activity cases and vaccinated cases was used in the calibration of the model.

3.2 MSM population data

Population data on the number of MSM individuals was obtained from various sources. This information is used to inform the initial number of vaccinated and unvaccinated susceptible individuals in each activity group at the start of the second wave of mpox in Portugal in June 2023. The number of MSM individuals in the population was obtained as 3% [15, 1] of the population of men in Portugal between the ages of 15 and 65 years old. MSM individuals were then categorised in high sexual activity and low sexual activity according to the number of non-steady partners (with whom they have intercourse) in the last 12 months according to the European Men Who Have Sex With Men Internet Survey (EMIS) [16] conducted in 2017. We assumed that the high sexual activity group corresponds to individuals with more than 30 non-steady partners in the last 12 months, which would result in approximately 2.5 non-steady partners per month. This results in 32.3% of the MSM population having no non-steady partners in the last 12 months (these are removed from the population), 62.1% having low sexual activity and 5.6% having high sexual activity.

The mpox vaccination program started in July 2022, with 4574 individuals being vaccinated up until June 29th 2023 [12]. Vaccination eligibility criteria included individuals with behaviours associated with high sexual activity [13]. Furthermore, following the demographic statistics of men in Portugal, we assumed that 32% of low sexual activity MSM individuals are older than 50 years, and thus were vaccinated for smallpox prior to 1977. For the high sexual activity group we assumed all individuals to be below 50 years old, given that sexual activity tends to decrease with age [18] and there are few cases above 50 years old along with only one case having received it as a child in 1976, with no known sexual activity behaviour.

Table 1 presents the values used to inform the initial conditions of the model as of the start of the second wave in July 2023.

3.3 Parameter data

The description, values and prior distributions used to inform the calibration procedure are depicted in table 2, along with the sources used to inform the parameters.

The vaccination rate in the low activity group, after June 2023 was assumed to be 0 according to vaccination eligibility criteria [13]. The vaccination rate in the high activity group was assumed to be equal to 1.5 vaccines per infected

Table 1: Initial conditions for the number of MSM individuals in each compartment at the start of the second mpox wave in July 2023. Normal distributions are truncated at 0.

initial condition	definition	value
$S_{v,h}(0)$	number of vaccinated susceptible individuals in the high activity group	4574
$E_{v,h}(0)$	number of vaccinated exposed individuals in the high activity group	0
$I_{v,h}(0)$	number of vaccinated infectious individuals in the high activity group	0
$S_{v,l}(0)$	number of vaccinated susceptible individuals in the low activity group	18427
$E_{v,l}(0)$	number of vaccinated exposed individuals in the low activity group	0
$I_{v,l}(0)$	number of vaccinated infectious individuals in the low activity group	0
$S_{u,h}(0)$	number of unvaccinated susceptible individuals in the high activity group	500
$E_{u,h}(0)$	number of unvaccinated exposed individuals in the high activity group	Normal(5,1)
$I_{u,h}(0)$	number of unvaccinated infectious individuals in the high activity group	Normal(5,1)
$S_{u,l}(0)$	number of unvaccinated susceptible individuals in the low activity group	37841
$E_{u,l}(0)$	number of unvaccinated exposed individuals in the low activity group	0
$I_{u,l}(0)$	number of unvaccinated infectious individuals in the low activity group	0
$R_h(0)$	number of recovered individuals in the high activity group	0
$R_l(0)$	number of recovered individuals in the low activity group	0

Table 2: Model parameters definition, values and prior information used in the calibration procedure. Rates are given in a weekly time unit. Normal distributions are truncated at 0.

Parameter	definition	value/prior	source
θ_s	mixing between sexual activity groups (steady partners)	Uniform(0,1)	[19]
$\alpha_{s,h}$	rate of steady partners' acquisition in the high activity group	0.04	[16]
$\alpha_{s,l}$	rate of steady partners' acquisition in the low activity group	0.04	[16]
θ_n	mixing between sexual activity groups (non-steady partners)	Uniform(0,1)	[19]
$\alpha_{n,h}$	rate of non-steady partners' acquisition in the high activity group	Normal(7,7)	[19, 16]
$\alpha_{n,l}$	rate of non-steady partners' acquisition in the low activity group	0.13	[16]
e	vaccine effectiveness	Beta(0.85,0.15)	[4, 19]
q_h	proportion of high activity group with steady partner	0.49	[16]
q_l	proportion of low activity group with steady partner	0.49	[16]
β	probability of transmission of mpox per sexual act	Uniform(0,1)	[19]
$c_{h,h}$	rate of sexual acts (steady, high with high)	3.8	[18]
$c_{h,l}$	rate of sexual acts (steady, high with low)	1.2	[18]
$c_{l,h}$	rate of sexual acts (steady, low with high)	1.2	[18]
$c_{l,l}$	rate of sexual acts (steady, low with low)	1	[18]
ϕ_h	vaccination rate in the high activity group	0.00358	assumed
μ	rate of entry and exit out of the population	0.00038	assumed
ϵ	latent removal rate (1/epsilon is the avg latent period)	Normal(1,0.5)	[7]
γ	recovery rate (1/gamma is the avg infectious period)	Normal(1,0.5)	[7]
ϕ_l	vaccination rate in the low activity group	0	assumed
a	reporting probability	0.62 and 0.31	[3]

case [19]. Population renewal rate μ was calculated assuming 50 years of sexual activity. We consider a reporting rate equal to that estimated for the first wave [3]. We also explore a different assumption on this value by considering also a reporting probability of 0.31, i.e. half of the original reporting rate. This sensitivity analysis is depicted in section 6. Lower reporting rates can result from changes in public awareness about the disease, which may have influenced the smaller outbreak observed during the second mpox wave.

4 Bayesian Model

Model (1) was calibrated using Bayesian techniques. The model was implemented in the software environment for statistical computing and graphics `R` using the package `cmdstanr` [9]. Inference is performed via the No-U-Turn sampler, which is an extension of Hamiltonian Monte Carlo (HMC) algorithm [10].

The HMC algorithm was run with 4 chains, with 3000 iterations each. The first 1500 were discarded as warm up. Convergence to the posterior distribution is assessed via the *R-hat* and *effective sample size* statistics along with diagnostic plots.

4.1 Likelihood

We assumed that the number of weekly reported mpox cases follows a Poisson model with mean given by

$$\lambda_t = a * \epsilon * \sum_{i=u,v} \sum_{j=h,l} E_{i,j,t},$$

for $t = 1, ..., 15$, which corresponds to a fraction of the weekly influx of infectious cases in the population(as given by the multiplier a). Moreover, we assumed that the number of infections in individuals in the high sexual activity group follows a binomial distribution with parameters $N = 118$ (total number of infections) and

$$p_h = \frac{\sum_{t=1}^{15} \sum_{i=u,v} E_{i,h,t}}{\sum_{t=1}^{15} \sum_{i=u,v} \sum_{j=h,l} E_{i,j,t}},$$

denoting the proportion of newly infectious that belong to the high activity group.

Likewise for the total number of infections in vaccinated individuals we assume $N = 118$ and

$$p_v = \frac{\sum_{t=1}^{15} \sum_{i=h,l} E_{v,i,t}}{\sum_{t=1}^{15} \sum_{i=u,v} \sum_{j=h,l} E_{i,j,t}},$$

corresponding to the proportion of newly infectious with vaccination.

4.2 Prior probability distribution

Prior probability distribution information is detailed in table 2.

5 Results

5.1 Posterior probability distribution

Table 3 presents the 2.5%, 50% and 97.5% percentiles obtained from the posterior distribution for each parameter along with diagnostic statistics *R-hat* and *effective sample size* (bulk and tail) [10]. The *R-hat* statistic is below 1.01 for all parameters which suggests that convergence to the posterior distribution was attained. Moreover, *effective sample size* statistics are large and the chains mixed well and are also stationary, as depicted in figure 2 (right). Posterior distributions are compared with prior distributions in figure 2 (left).

Table 3: Posterior distribution statistics

parameter	q025	q50	q975	rhat	ess_bulk	ess_tail
θ_s	0.92	0.97	0.99	1.00	1765.00	3266.00
θ_n	0.03	0.51	0.98	1.00	5076.00	3447.00
$\alpha_{n,h}$	7.91	13.08	24.05	1.00	2017.00	3616.00
e	0.98	0.99	0.99	1.00	5483.00	4534.00
β	0.30	0.57	0.95	1.00	1772.00	2379.00
ϵ	0.91	1.01	1.11	1.00	5298.00	3793.00
γ	0.43	0.52	0.61	1.00	5120.00	3470.00
$E_{u,h}(0)$	3.34	5.16	7.08	1.00	5724.00	3438.00
$I_{u,h}(0)$	3.38	5.21	7.07	1.00	6212.00	3801.00

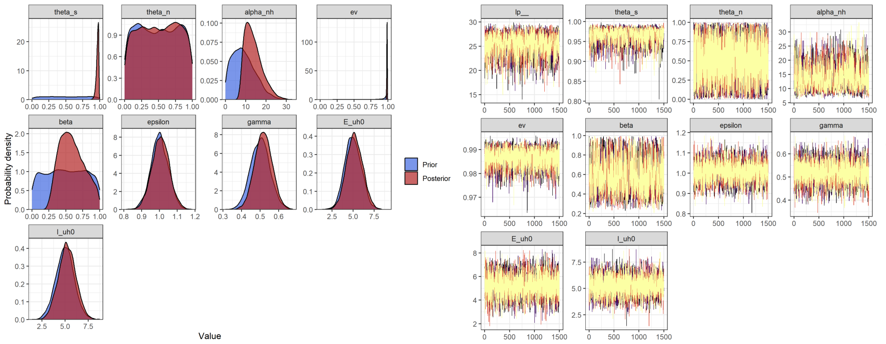

Figure 2: Prior and posterior distributions (left). Trace plots (right).

5.2 Posterior predictive check

Figure 3 displays the weekly number of mpox cases (black dots) compared to the mean of the posterior predictive distribution along with 2.5% and 97.5% percentiles (95% credible interval) (left) and the posterior distribution (violin plots) for the proportion of mpox cases that belong to the high sexual activity group and the proportion of vaccinated cases (right). We see that the model explains the observed data.

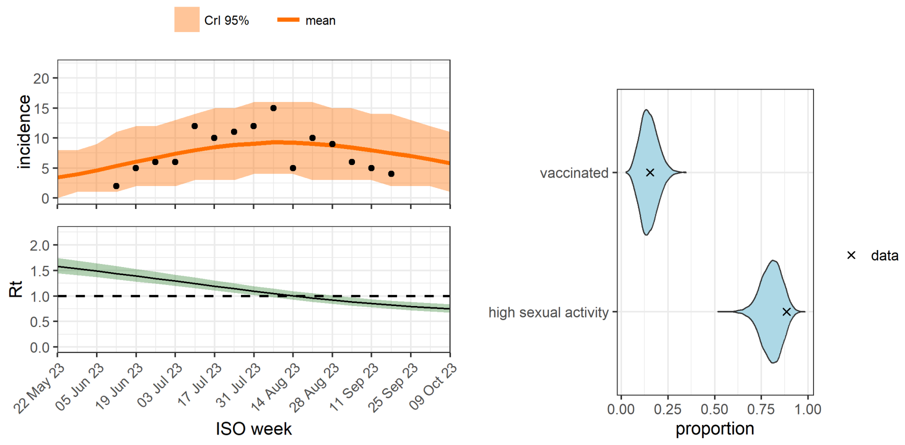

Figure 3: Posterior predictive check for the number of mpox cases, with 95% CrI and reproduction number (left). Posterior predictive check for the proportion of cases in the high activity group and vaccinated cases (right).

5.3 Elasticity values

Table 4 depicts the cumulative elasticities for each MSM subgroup along with 95% credible intervals. These represent the relative contribution of each group towards the Rt. These computations were performed using equation (10). The cumulative elasticity for the high sexual activity group and low sexual activity group were obtained as follows:

$$e_{\text{h}} = e_{\text{vc-h}} + e_{\text{uv-h}},$$

and

$$e_{\text{l}} = e_{\text{vc-l}} + e_{\text{uv-l}},$$

representing the sum of the elasticities of the respective subgroups of vaccinated and unvaccinated individuals.

The relative contributions presented in the main text are obtained by computing the ratio between the elasticities of the given groups. For example, the relative contribution of the high sexual activity group compared to the low sexual activity group is given by: $\frac{e_{\text{h}}}{e_{\text{l}}} = 120.57$ (95% CrI: $30.72 - 3553.56$) and vaccinated group compared to the unvaccinated group (within those belonging to the high sexual activity group) is given by $\frac{e_{\text{vc-h}}}{e_{\text{uv-h}}} = 0.123$ (95% CrI: $0.068 - 0.208$). These elasticities were computed for the first week of the study period.

Table 4: Cumulative elasticity values, along with 95% credible intervals, for each group: high sexual activity (h), low sexual activity (l), unvaccinated high sexual activity (uv-h), vaccinated high sexual activity (vc-h), unvaccinated low sexual activity (uv-l) and vaccinated low sexual activity (vc-l).

groups	elasticity	CrI 95%
h	0.99177	0.96848-0.99972
l	0.00823	0.00028-0.03152
uv-h	0.88165	0.81511-0.9295
vc-h	0.10863	0.06313-0.17015
uv-l	0.00818	0.00028-0.03133
vc-l	0.00005	0-0.00024

6 Sensitivity analysis

In this section we replicate the previous analysis by assuming a mpox reporting probability of 0.31. Table 5 and 6 refers to the posterior distributions statistics and elasticity values, respectively. Figure 4 refers to the diagnostic plots, i.e. prior and posterior distributions along with trace plots. Figure 5 presents the model fit and evolution of the reproduction number. Figure 6 displays the scenario analysis performed in the main text, assuming a reporting probability of 0.31. We can observe that, within this assumption, the model is still able to properly fit to the data. Moreover we can observe that the major change occurs in the reproduction number, which is estimated to take higher values during the early weeks of the outbreak. This comparison can be observed in figures 5 and 3.

Table 5: Posterior distribution statistics

	variable	q025	q50	q975	rhat	ess_bulk	ess_tail
1	θ_s	0.94	0.98	0.99	1.00	1593.00	1347.00
2	θ_n	0.03	0.51	0.98	1.00	5349.00	3448.00
3	α_{nh}	10.16	14.75	24.84	1.00	1361.00	1473.00
4	e	0.98	0.99	0.99	1.00	3834.00	3829.00
5	β	0.40	0.70	0.98	1.00	1292.00	1455.00
6	ϵ	0.91	1.00	1.10	1.00	5054.00	4080.00
7	γ	0.41	0.50	0.60	1.00	4403.00	3806.00
8	$E_{uh}(0)$	3.06	4.97	6.80	1.00	4619.00	2685.00
9	$I_{uh}(0)$	3.03	4.91	6.89	1.00	4557.00	3400.00

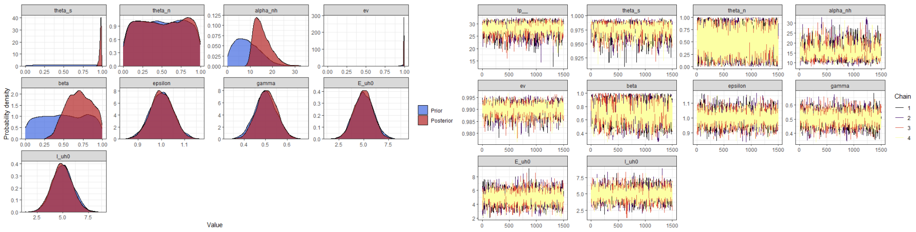

Figure 4: Prior and posterior distributions (left). Trace plots (right).

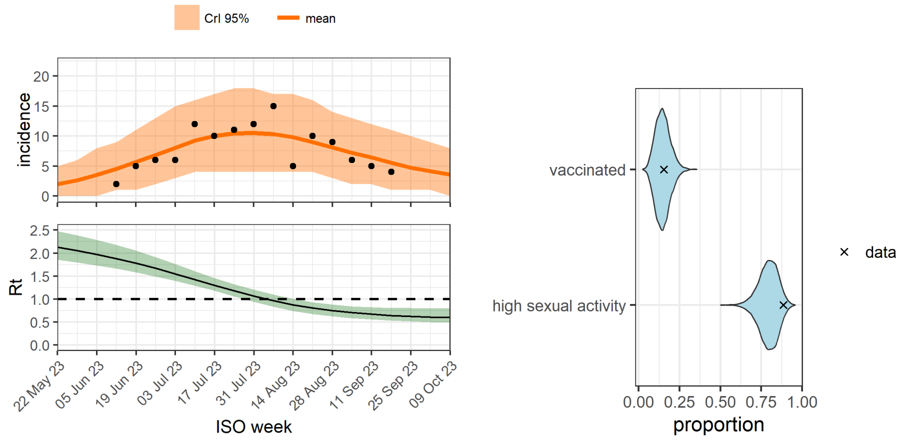

Figure 5: Posterior predictive check for the number of mpox cases, with 95% CrI and reproduction number (left). Posterior predictive check for the proportion of cases in the high activity group and vaccinated cases (right).

References

[1] S. Aboim. "Homossexualidade e bissexualidade: práticas, atracção e orientação sexual". In: Editorial Bizâncio, 2016, pp. 266–290.

[2] Leonardo Angeli et al. "Who acquires infection from whom? A sensitivity analysis of transmission dynamics during the early phase of the COVID-19 pandemic in Belgium". In: *Journal of Theoretical Biology* 581 (2024), p. 111721. DOI: `10.1016/j.jtbi.2024.111721`.

[3] Vítor Borges et al. "Viral genetic clustering and transmission dynamics of the 2022 MPOX outbreak in Portugal". In: *Nature Medicine* 29.10 (Sept. 2023), pp. 2509–2517. DOI: `10.1038/s41591-023-02542-x`.

[4] Alexandra F. Dalton et al. "Estimated effectiveness of JYNNEOS vaccine in preventing Mpox: A multijurisdictional case-control study — United States, August 19, 2022–March 31, 2023". In: *MMWR. Morbidity and Mortality Weekly Report* 72.20 (May 2023), pp. 553–558. DOI: `10.15585/mmwr.mm7220a3`.

[5] O. Diekmann, J. A. Heesterbeek, and M. G. Roberts. "The construction of next-generation matrices for compartmental epidemic models". In:

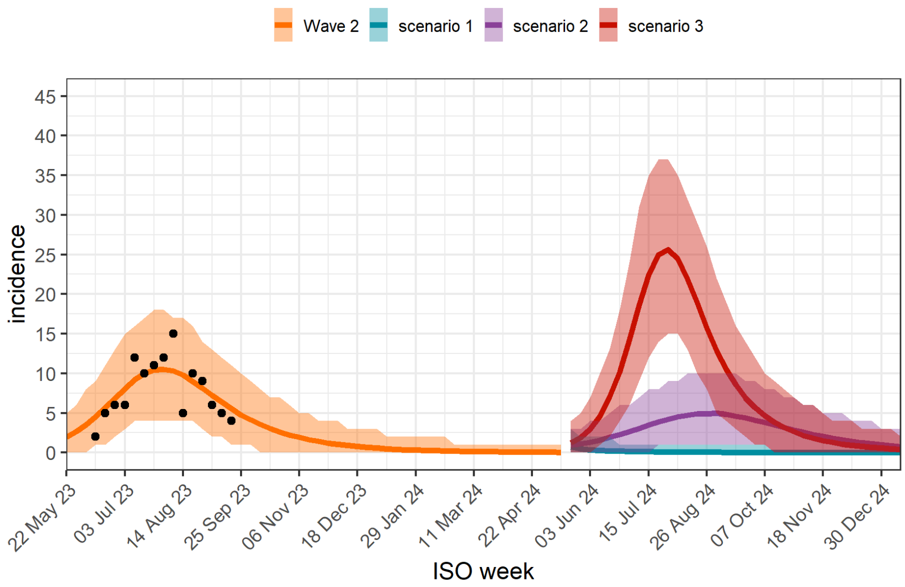

Figure 6: Epidemiological scenarios for the 2024 mpox season.

Table 6: Cumulative elasticity values, along with 95% credible intervals, for each group: high sexual activity (h), low sexual activity (l), unvaccinated high sexual activity (uv-h), vaccinated high sexual activity (vc-h), unvaccinated low sexual activity (uv-l) and vaccinated low sexual activity (vc-l).

groups	elasticity	CrI95
h	0.99447	0.98085-0.99983
l	0.00553	0.00017-0.01915
uv-h	0.91048	0.86314-0.94404
vc-h	0.08307	0.05078-0.12808
uv-l	0.00551	0.00017-0.01905
vc-l	0.00002	0-0.00010

Journal of The Royal Society Interface 7.47 (2009), pp. 873–885. DOI: 10.1098/rsif.2009.0386.

[6] Pauline van den Driessche. "Reproduction numbers of infectious disease models". In: *Infectious Disease Modelling* 2.3 (2017), pp. 288–303. DOI: 10.1016/j.idm.2017.06.002.

[7] ECDC. *Considerations for contact tracing during the monkeypox outbreak in Europe*. Tech. rep. Stockholm:ECDC, 2022.

[8] P. E. Fine et al. "The transmission potential of monkeypox virus in human populations". In: *International Journal of Epidemiology* 17.3 (1988), pp. 643–650. DOI: 10.1093/ije/17.3.643.

[9] Jonah Gabry, Rok Češnovar, and Andrew Johnson. *cmdstanr: R Interface to 'CmdStan'*. https://mc-stan.org/cmdstanr/, https://discourse.mc-stan.org. 2023.

[10] Andrew Gelman et al. *Bayesian Data Analysis*. 2nd ed. Chapman and Hall/CRC, 2004.

[11] Ramy Mohamed Ghazy et al. "Systematic Review on the Efficacy, Effectiveness, Safety, and Immunogenicity of Monkeypox Vaccine". In: *Vaccines* 11.11 (2023). ISSN: 2076-393X. DOI: 10.3390/vaccines11111708. URL: https://www.mdpi.com/2076-393X/11/11/1708.

[12] The Directorate-General of Health. *Mpox em Portugal e no Mundo - Informação mensal a 30 de junho de 2023*. Tech. rep. DGS, 2023.

[13] The Directorate-General of Health. *Mpox em Portugal e no Mundo - Informação mensal a 31 de dezembro de 2023*. Tech. rep. DGS, 2023.

[14] Z. Jezek et al. "Human Monkeypox: A study of 2,510 contacts of 214 patients". In: *Journal of Infectious Diseases* 154.4 (Oct. 1986), pp. 551–555. DOI: 10.1093/infdis/154.4.551.

[15] Daniel E. Mauck et al. "Population-based methods for estimating the number of men who have sex with men: A systematic review". In: *Sexual Health* 16.6 (2019), p. 527. DOI: 10.1071/sh18172.

[16] The EMIS Network. *the European Men-Who-Have-Sex-With-Men Internet Survey. Key findings from 50 countries.* Tech. rep. Stockholm: European Centre for Disease Prevention and Control, 2019.

[17] Rodrigo Núñez-Cortés et al. "Risk profile and mode of transmission of mpox: A rapid review and individual patient data meta-analysis of case studies". In: *Reviews in Medical Virology* 33.2 (Nov. 2022). DOI: 10.1002/rmv.2410.

[18] Kristin M. Wall, Robert Stephenson, and Patrick S. Sullivan. "Frequency of sexual activity with most recent male partner among young, internet-using men who have sex with men in the United States". In: *Journal of Homosexuality* 60.10 (Sept. 2013), pp. 1520–1538. DOI: 10.1080/00918369.2013.819256.

[19] Maria Xiridou et al. "The fading of the MPOX outbreak among men who have sex with men: A mathematical modelling study". In: *The Journal of Infectious Diseases* (2023). DOI: 10.1093/infdis/jiad414.
