# Supplementary material for: Viral genetics and transmission dynamics in the second wave of mpox outbreak in Portugal and forecasting public health scenarios
Source: Emerg Microbes Infect. 2024 Oct 3;13(1):2412635. doi: 10.1080/22221751.2024.2412635 (PMC11486115; doi:10.1080/22221751.2024.2412635)
Supplement: Additional file 2.pdf [file TEMI_A_2412635_SM0277.pdf]

We gratefully acknowledge the following Authors from the Originating laboratories responsible for obtaining the specimens, as well as the Submitting laboratories where the genome data were generated and shared via GISAID, on which this research is based.

All Submitters of data may be contacted directly via www.gisaid.org

Authors are sorted alphabetically.

Accession ID	Originating Laboratory	Submitting Laboratory	Authors
EPI_ISL_16679238, EPI_ISL_16679239, EPI_ISL_16679240	Los Angeles County Public Health Laboratories	Los Angeles County Public Health Laboratories	P. Hemarajata et al.
EPI_ISL_16930160, EPI_ISL_16930168, EPI_ISL_16930171, EPI_ISL_16930177, EPI_ISL_16930180, EPI_ISL_16930183	California Department of Public Health	California Department of Public Health	Probert,W., Espinosa,A., Kath,C., Haw,M., O'Neil,R., Bell,J. and Hacker,J.
EPI_ISL_16955216, EPI_ISL_16955217, EPI_ISL_16955218, EPI_ISL_16955219, EPI_ISL_16955220, EPI_ISL_16955221	Public Health Laboratory, NYC Department of Health and Mental Hygiene	Public Health Laboratory, NYC Department of Health and Mental Hygiene	Wang,J.C., Amin,H.S., Clabby,T.T., Taki,F., Su,M., Rahat,A., De La Cruz,N., Olsen,A., Thi,C., Silver,S., Akther,S., Chowdhury,M., Omoregie,E. and Hughes,S.
EPI_ISL_16997397, EPI_ISL_16997402	California Department of Public Health	California Department of Public Health	Probert,W., Espinosa,A., Kath,C., Haw,M., O'Neil,R., Bell,J. and Hacker,J.
EPI_ISL_16997404, EPI_ISL_16997405, EPI_ISL_16997406	Quest Diagnostics Nichols Institute	Los Angeles County Public Health Laboratories	P. Hemarajata et al.
EPI_ISL_16997407, EPI_ISL_16997408, EPI_ISL_16997409, EPI_ISL_16997410, EPI_ISL_16997411, EPI_ISL_16997412	Los Angeles County Public Health Laboratories	Los Angeles County Public Health Laboratories	P. Hemarajata et al.
EPI_ISL_16997413, EPI_ISL_16997441, EPI_ISL_16997442, EPI_ISL_16997443	Kaiser Permanente Chino Hills Regional Reference Laboratories	Los Angeles County Public Health Laboratories	P. Hemarajata et al.
EPI_ISL_16997454	Laboratory Corporation of America	Los Angeles County Public Health Laboratories	P. Hemarajata et al.
EPI_ISL_16997458	Los Angeles County Public Health Laboratories	Los Angeles County Public Health Laboratories	P. Hemarajata et al.
EPI_ISL_16997460, EPI_ISL_16997461, EPI_ISL_16997462, EPI_ISL_16997466, EPI_ISL_16997467, EPI_ISL_16997468, EPI_ISL_16997469	Quest Diagnostics Nichols Institute	Los Angeles County Public Health Laboratories	P. Hemarajata et al.
EPI_ISL_17008293, EPI_ISL_17008294, EPI_ISL_17008295, EPI_ISL_17008296	Tokyo Metropolitan Institute of Public Health, Department of Microbiology	Tokyo Metropolitan Institute of Public Health, Department of Microbiology	Kasuya,F., Negishi,A., Kumagai,R., Hasegawa,M., Fujiwara,T., Miyake,H., Nagashima,M. and Sadamasu,K.
EPI_ISL_17012019, EPI_ISL_17012020, EPI_ISL_17012021, EPI_ISL_17012022	Laboratorio de Virus Exantematicos, Gastroentéricos y Otros Transmitidos por Vectores	Centro de Referencia Nacional de Genomica, Secuenciacion y Bioinformatica GENSBIO, INSPI-CZ9	Andrés Carrazco, Silvia Salgado, Diana Gutiérrez, Damaris Alarcón, Andrés Herrera, Andrés Tinizaray, Martha Sánchez, Johanna Parrales, Diego Morales, Jorge Bejarano, Leandro Patiño.
EPI_ISL_17152764	Centers for Disease Control, R.O.C. (Taiwan)	Centers for Disease Control, R.O.C. (Taiwan)	Lin JH, Chiu SC, Huang HI, Huang WL, Li TY, Fann WB, Hsieh PY, Yang JY
EPI_ISL_17152842	Centers for Disease Control, R.O.C. (Taiwan)	Centers for Disease Control, R.O.C. (Taiwan)	Lin JH, Chiu SC, Huang HI, Huang LW, Li TY, Fann WB, Hsieh PY, Yang JY
EPI_ISL_17152843	Centers for Disease Control, R.O.C. (Taiwan)	Centers for Disease Control, R.O.C. (Taiwan)	Lin JH, Chiu SC, Huang HI, Huang WL, Li TY, Fann WB, Hsieh PY, Yang JY
EPI_ISL_17170669, EPI_ISL_17170670, EPI_ISL_17170671	California Department of Public Health	California Department of Public Health	Haw, M. , Kath, C., Espinosa, A., O'Neil, R., and Hacker, J.
EPI_ISL_17206615, EPI_ISL_17206616, EPI_ISL_17206617	California Department of Public Health	California Department of Public Health	Haw,M., Kath,C., Espinosa,A., O'Neil,R., and Hacker,J.
EPI_ISL_17211324, EPI_ISL_17211325, EPI_ISL_17211330	Kaiser Permanente Chino Hills Regional Reference Laboratories	Los Angeles County Public Health Laboratories	P. Hemarajata et al.
EPI_ISL_17211331	Los Angeles County Public Health Laboratories	Los Angeles County Public Health Laboratories	P. Hemarajata et al.
EPI_ISL_17211332	Quest Diagnostics Nichols Institute	Los Angeles County Public Health Laboratories	P. Hemarajata et al.
EPI_ISL_17211335	Laboratory Corporation of America	Los Angeles County Public Health Laboratories	P. Hemarajata et al.
EPI_ISL_17222827, EPI_ISL_17222828, EPI_ISL_17222829	Viral and Rickettsial Disease Laboratory (VRDL) California Department of Public Health (CDPH)	Viral and Rickettsial Disease Laboratory (VRDL) California Department of Public Health (CDPH)	Haw,M., Kath,C., Espinosa,A., O'Neil,R. and Hacker,J.
EPI_ISL_17246657, EPI_ISL_17246659	Fumi Kasuya Tokyo Metropolitan Institute of Public Health, Department of Microbiology	Fumi Kasuya Tokyo Metropolitan Institute of Public Health, Department of Microbiology	Kasuya,F., Negishi,A., Kumagai,R., Hasegawa,M., Fujiwara,T., Miyake,H., Nagashima,M. and Sadamasu,K.
EPI_ISL_17271956, EPI_ISL_17271957	Rhode Island State Health Laboratory	Rhode Island State Health Laboratory	Kristin Carpenter-Azevedo, Sean Sierra-Patev, Richard C. Huard
EPI_ISL_17319547, EPI_ISL_17319548, EPI_ISL_17319549, EPI_ISL_17319550, EPI_ISL_17319551, EPI_ISL_17319552, EPI_ISL_17319553, EPI_ISL_17319554, EPI_ISL_17319555	Department of Clinical Sciences, Institute of Tropical Medicine	Department of Clinical Sciences, Institute of Tropical Medicine	Mertes,H., Rezende,A.M., Naesens,R., de Block,T., Michiels,J., Coppens,J., Van Dijck,C., Bomans,P., Arien,K., Bottieau,E., Van Esbroeck,M., Liesenborghs,L. and Vercauteren,K.
EPI_ISL_17319556, EPI_ISL_17428282, EPI_ISL_17428283, EPI_ISL_17428284, EPI_ISL_17428286	Center of Diagnostics and Vaccine Development, Centers for Disease Control	Center of Diagnostics and Vaccine Development, Centers for Disease Control	Lin,J.-H., Chiu,S.-C., Huang,H.-I., Huang,W.-L., Li,T.-Y., Fann,W.-B., Hsieh,P.-Y. and Yang,J.-Y.
EPI_ISL_17445514, EPI_ISL_17445515, EPI_ISL_17445516, EPI_ISL_17445517, EPI_ISL_17445518, EPI_ISL_17445519	Tokyo Metropolitan Institute of Public Health	Tokyo Metropolitan Institute of Public Health	Fumi Kasuya, Wakaba Okada, Ryota Kumagai, Sachiko Harada, Arisa Amano, Michiya Hasegawa, Mami Nagashima, Kenji Sadamasu
EPI_ISL_17472048	Department of Infectious Diseases, National Institute of Health Doutor Ricardo Jorge, Portugal (INSA)	Department of Infectious Diseases, National Institute of Health Doutor Ricardo Jorge, Portugal (INSA)	Isidro,J., Borges,V., Pinto,M., Sobral,D., Santos,J., Nunes,A., Mixao,V., Ferreira,R., Santos,D., Duarte,S., Vieira,L., Borrego,M.J., Nuncio,S., Lopes de Carvalho,I., Pelerito,A., Cordeiro,R. and Gomes,J.P.
EPI_ISL_17485343	Laboratorio de Enterovirus, Instituto Oswaldo Cruz, Fiocruz	Instituto Oswaldo Cruz FIOCRUZ - Laboratory of Respiratory Viruses and Measles (LVRS)	Paola Resende, Elisa Cavalcante Pereira, Bruna Mendonça da Silva, Jéssica Graça Macedo de Carvalho, Larissa Macedo Pinto, Victor Guimaraes, Marilda Siqueira, Renan da Silva Faustino, Marilia Santini, Beatriz Grinsztejn, Mayara Secco Torres da Silva, Edson Elias da Silva on behalf of the Fiocruz Genomic Surveillance Network
EPI_ISL_17502583	Public Health Laboratory, Public Health Service Amsterdam, The Netherlands	Department of Medical Microbiology & Infection prevention, Amsterdam University Medical Centers location AMC	Matthijs Welkers, Jelle Koopsen, Robin van Houdt, Marcel Jonges, Sebastien Matamoros, Sjoerd Rebers, Fokla Zorgdrager, Sylvia Bruisten, Akke Cornelissen, Janke Schinkel, Ewout Fanoy, Roisin Bavalia, Menno de Jong and Mariken van der Lubben on behalf of the Amsterdam Regional Genomic epidemiology and Outbreak Surveillance (ARGOS) consortium
EPI_ISL_17518107	Virology Section, Division of Microbiology,Osaka Institute of Public Health	Virology Section, Division of Microbiology,Osaka Institute of Public Health	Daiki Kanbayashi, Takako Kurata, Takuya Kawahata, Fumiya Bannno, Minami Hama, Kazushi Motomuta
EPI_ISL_17529367, EPI_ISL_17529368	Laboratorio de Virus Exantematicos, Gastroentéricos y Otros Transmitidos por Vectores	Centro de Referencia Nacional de Genomica, Secuenciacion y Bioinformatica GENSBIO, INSPI-CZ9	Andrés Carrazco*, SIlvia Salgado, Diana Gutiérrez, Damaris Alarcón, Andrés Tinizaray, Ruth Gómez, Martha Sánchez, Johanna Parrales, Eva Nicola, Jorge Bejarano, Leandro Patiño.
EPI_ISL_17536780	Department of Virology, National Institute of Health, Islamabad, Pakistan	Department of Virology, National Institute of Health, Islamabad, Pakistan	Massab Umair, Muhammad Ammar, Syed Adnan Haider, Rabia Hakim, Qasim Malik, Muhammad Salman, Ghazala Parveen, and Naseem Akhtar
EPI_ISL_17536782, EPI_ISL_17536783, EPI_ISL_17536784, EPI_ISL_17536785	Laboratorio de Enterovirus, Instituto Oswaldo Cruz, Fiocruz	Instituto Oswaldo Cruz FIOCRUZ - Laboratory of Respiratory Viruses and Measles (LVRS)	Paola Resende, Elisa Cavalcante Pereira, Bruna Mendonça da Silva, Jéssica Graça Macedo de Carvalho, Larissa Macedo Pinto, Victor Guimaraes, Marilda Siqueira, Renan da Silva Faustino, Marilia Santini, Edson Elias da Silva on behalf of the Fiocruz Genomic Surveillance Network
EPI_ISL_17582853	Quest Diagnostics Nichols Institute	Los Angeles County Public Health Laboratories	P. Hemarajata et al.
EPI_ISL_17592665, EPI_ISL_17592666, EPI_ISL_17592667, EPI_ISL_17592668, EPI_ISL_17592669, EPI_ISL_17592670	Tokyo Metropolitan Institute of Public Health	Tokyo Metropolitan Institute of Public Health	Fumi Kasuya, Wakaba Okada, Ryota Kumagai, Sachiko Harada, Arisa Amano, Michiya Hasegawa, Mami Nagashima, Kenji Sadamasu
EPI_ISL_17595302, EPI_ISL_17595303	Quest Diagnostics Nichols Institute	Los Angeles County Public Health Laboratories	P. Hemarajata et al.
EPI_ISL_17595304, EPI_ISL_17595305, EPI_ISL_17595306, EPI_ISL_17595307, EPI_ISL_17595308	Kaiser Permanente Chino Hills Regional Reference Laboratories	Los Angeles County Public Health Laboratories	P. Hemarajata et al.
EPI_ISL_17614017, EPI_ISL_17614021, EPI_ISL_17614030, EPI_ISL_17614033, EPI_ISL_17614038, EPI_ISL_17614039, EPI_ISL_17614040, EPI_ISL_17614041, EPI_ISL_17614042	Laboratorio de Enterovirus, Instituto Oswaldo Cruz, Fiocruz	Instituto Oswaldo Cruz FIOCRUZ - Laboratory of Respiratory Viruses and Measles (LVRS)	Paola Resende, Elisa Cavalcante Pereira, Bruna Mendonça da Silva, Jéssica Graça Macedo de Carvalho, Larissa Macedo Pinto, Victor Guimaraes, Marilda Siqueira, Renan da Silva Faustino, Marilia Santini, Edson Elias da Silva on behalf of the Fiocruz Genomic Surveillance Network
EPI_ISL_17665624, EPI_ISL_17665625, EPI_ISL_17665626, EPI_ISL_17665627	Tokyo Metropolitan Institute of Public Health	Tokyo Metropolitan Institute of Public Health	Fumi Kasuya, Wakaba Okada, Ryota Kumagai, Sachiko Harada, Arisa Amano, Michiya Hasegawa, Mami Nagashima, Kenji Sadamasu
EPI_ISL_17672236	Laboratorio Estatal de Salud Pública Puebla	Instituto de Diagnostico y Referencia Epidemiologicos (INDRE)	Abril Rodríguez-Maldonado; Claudia Wong-Arámbula; Silvia Rivero-Arredondo; Ruth Madera-Sandoval; Joaquín Quiroz-Mercado; Fernando González-Domínguez; Lucia Hernández-Rivas, Irma López-Martínez; Ernesto Ramírez-González; Maribel González-Villa
EPI_ISL_17672237	LESP Campeche	Instituto de Diagnostico y Referencia Epidemiologicos (INDRE)	Abril Rodríguez-Maldonado; Claudia Wong-Arámbula; Silvia Rivero-Arredondo; Ruth Madera-Sandoval; Joaquín Quiroz-Mercado; Fernando González-Domínguez; Lucia Hernández-Rivas, Irma López-Martínez; Ernesto Ramírez-González; Maribel González-Villa
EPI_ISL_17672238	LESP State of Mexico	Instituto de Diagnostico y Referencia Epidemiologicos (INDRE)	Abril Rodríguez-Maldonado; Claudia Wong-Arámbula; Silvia Rivero-Arredondo; Ruth Madera-Sandoval; Joaquín Quiroz-Mercado; Fernando González-Domínguez; Lucia Hernández-Rivas, Irma López-Martínez; Ernesto Ramírez-González; Maribel González-Villa
EPI_ISL_17672239	LESP Chiapas	Instituto de Diagnostico y Referencia Epidemiologicos (INDRE)	Abril Rodríguez-Maldonado; Claudia Wong-Arámbula; Silvia Rivero-Arredondo; Ruth Madera-Sandoval; Joaquín Quiroz-Mercado; Fernando González-Domínguez; Lucia Hernández-Rivas, Irma López-Martínez; Ernesto Ramírez-González; Maribel González-Villa
EPI_ISL_17672240	LESP Coahuila	Instituto de Diagnostico y Referencia Epidemiologicos	Abril Rodríguez-Maldonado; Claudia Wong-Arámbula; Silvia Rivero-Arredondo; Ruth Madera-Sandoval; Joaquín Quiroz-Mercado; Fernando González-Domínguez; Lucia Hernández-Rivas, Irma López-Martínez; Ernesto

		(INDRE)	Ramírez-González; Maribel González-Villa
EPI_ISL_17672241	LESP Jalisco	Instituto de Diagnostico y Referencia Epidemiologicos (INDRE)	Abril Rodríguez-Maldonado; Claudia Wong-Arámbula; Silvia Rivero-Arredondo; Ruth Madera-Sandoval; Joaquín Quiroz-Mercado; Fernando González-Domínguez; Lucia Hernández-Rivas, Irma López-Martínez; Ernesto Ramírez-González; Maribel González-Villa
EPI_ISL_17672242	LESP Nuevo Leon	Instituto de Diagnostico y Referencia Epidemiologicos (INDRE)	Abril Rodríguez-Maldonado; Claudia Wong-Arámbula; Silvia Rivero-Arredondo; Ruth Madera-Sandoval; Joaquín Quiroz-Mercado; Fernando González-Domínguez; Lucia Hernández-Rivas, Irma López-Martínez; Ernesto Ramírez-González; Maribel González-Villa
EPI_ISL_17672243	LESP Queretaro	Instituto de Diagnostico y Referencia Epidemiologicos (INDRE)	Abril Rodríguez-Maldonado; Claudia Wong-Arámbula; Silvia Rivero-Arredondo; Ruth Madera-Sandoval; Joaquín Quiroz-Mercado; Fernando González-Domínguez; Lucia Hernández-Rivas, Irma López-Martínez; Ernesto Ramírez-González; Maribel González-Villa
EPI_ISL_17672244	LESP Hidalgo	Instituto de Diagnostico y Referencia Epidemiologicos (INDRE)	Abril Rodríguez-Maldonado; Claudia Wong-Arámbula; Silvia Rivero-Arredondo; Ruth Madera-Sandoval; Joaquín Quiroz-Mercado; Fernando González-Domínguez; Lucia Hernández-Rivas, Irma López-Martínez; Ernesto Ramírez-González; Maribel González-Villa
EPI_ISL_17672245	LESP Tamaulipas	Instituto de Diagnostico y Referencia Epidemiologicos (INDRE)	Abril Rodríguez-Maldonado; Claudia Wong-Arámbula; Silvia Rivero-Arredondo; Ruth Madera-Sandoval; Joaquín Quiroz-Mercado; Fernando González-Domínguez; Lucia Hernández-Rivas, Irma López-Martínez; Ernesto Ramírez-González; Maribel González-Villa
EPI_ISL_17672246	LESP Veracruz	Instituto de Diagnostico y Referencia Epidemiologicos (INDRE)	Abril Rodríguez-Maldonado; Claudia Wong-Arámbula; Silvia Rivero-Arredondo; Ruth Madera-Sandoval; Joaquín Quiroz-Mercado; Fernando González-Domínguez; Lucia Hernández-Rivas, Irma López-Martínez; Ernesto Ramírez-González; Maribel González-Villa
EPI_ISL_17672247	LESP Quintana Roo	Instituto de Diagnostico y Referencia Epidemiologicos (INDRE)	Abril Rodríguez-Maldonado; Claudia Wong-Arámbula; Silvia Rivero-Arredondo; Ruth Madera-Sandoval; Joaquín Quiroz-Mercado; Fernando González-Domínguez; Lucia Hernández-Rivas, Irma López-Martínez; Ernesto Ramírez-González; Maribel González-Villa
EPI_ISL_17672249	LESP Oaxaca	Instituto de Diagnostico y Referencia Epidemiologicos (INDRE)	Abril Rodríguez-Maldonado; Claudia Wong-Arámbula; Silvia Rivero-Arredondo; Ruth Madera-Sandoval; Joaquín Quiroz-Mercado; Fernando González-Domínguez; Lucia Hernández-Rivas, Irma López-Martínez; Ernesto Ramírez-González; Maribel González-Villa
EPI_ISL_17672250	LESP Yucatan	Instituto de Diagnostico y Referencia Epidemiologicos (INDRE)	Abril Rodríguez-Maldonado; Claudia Wong-Arámbula; Silvia Rivero-Arredondo; Ruth Madera-Sandoval; Joaquín Quiroz-Mercado; Fernando González-Domínguez; Lucia Hernández-Rivas, Irma López-Martínez; Ernesto Ramírez-González; Maribel González-Villa
EPI_ISL_17672251	LESP Guanajuato	Instituto de Diagnostico y Referencia Epidemiologicos (INDRE)	Abril Rodríguez-Maldonado; Claudia Wong-Arámbula; Silvia Rivero-Arredondo; Ruth Madera-Sandoval; Joaquín Quiroz-Mercado; Fernando González-Domínguez; Lucia Hernández-Rivas, Irma López-Martínez; Ernesto Ramírez-González; Maribel González-Villa
EPI_ISL_17672252	LESP Veracruz	Instituto de Diagnostico y Referencia Epidemiologicos (INDRE)	Abril Rodríguez-Maldonado; Claudia Wong-Arámbula; Silvia Rivero-Arredondo; Ruth Madera-Sandoval; Joaquín Quiroz-Mercado; Fernando González-Domínguez; Lucia Hernández-Rivas, Irma López-Martínez; Ernesto Ramírez-González; Maribel González-Villa
EPI_ISL_17672253	LESP Mexico City	Instituto de Diagnostico y Referencia Epidemiologicos (INDRE)	Abril Rodríguez-Maldonado; Claudia Wong-Arámbula; Silvia Rivero-Arredondo; Ruth Madera-Sandoval; Joaquín Quiroz-Mercado; Fernando González-Domínguez; Lucia Hernández-Rivas, Irma López-Martínez; Ernesto Ramírez-González; Maribel González-Villa
EPI_ISL_17672254	LESP Durango	Instituto de Diagnostico y Referencia Epidemiologicos (INDRE)	Abril Rodríguez-Maldonado; Claudia Wong-Arámbula; Silvia Rivero-Arredondo; Ruth Madera-Sandoval; Joaquín Quiroz-Mercado; Fernando González-Domínguez; Lucia Hernández-Rivas, Irma López-Martínez; Ernesto Ramírez-González; Maribel González-Villa
EPI_ISL_17672255	LESP San Luis Potosi	Instituto de Diagnostico y Referencia Epidemiologicos (INDRE)	Abril Rodríguez-Maldonado; Claudia Wong-Arámbula; Silvia Rivero-Arredondo; Ruth Madera-Sandoval; Joaquín Quiroz-Mercado; Fernando González-Domínguez; Lucia Hernández-Rivas, Irma López-Martínez; Ernesto Ramírez-González; Maribel González-Villa
EPI_ISL_17672256	LESP Nuevo Leon	Instituto de Diagnostico y Referencia Epidemiologicos (INDRE)	Abril Rodríguez-Maldonado; Claudia Wong-Arámbula; Silvia Rivero-Arredondo; Ruth Madera-Sandoval; Joaquín Quiroz-Mercado; Fernando González-Domínguez; Lucia Hernández-Rivas, Irma López-Martínez; Ernesto Ramírez-González; Maribel González-Villa
EPI_ISL_17672257, EPI_ISL_17672258	LESP Quintana Roo	Instituto de Diagnostico y Referencia Epidemiologicos (INDRE)	Abril Rodríguez-Maldonado; Claudia Wong-Arámbula; Silvia Rivero-Arredondo; Ruth Madera-Sandoval; Joaquín Quiroz-Mercado; Fernando González-Domínguez; Lucia Hernández-Rivas, Irma López-Martínez; Ernesto Ramírez-González; Maribel González-Villa
EPI_ISL_17672259	LESP Mexico City	Instituto de Diagnostico y Referencia Epidemiologicos (INDRE)	Abril Rodríguez-Maldonado; Claudia Wong-Arámbula; Silvia Rivero-Arredondo; Ruth Madera-Sandoval; Joaquín Quiroz-Mercado; Fernando González-Domínguez; Lucia Hernández-Rivas, Irma López-Martínez; Ernesto Ramírez-González; Maribel González-Villa
EPI_ISL_17672260	LESP Veracruz	Instituto de Diagnostico y Referencia Epidemiologicos (INDRE)	Abril Rodríguez-Maldonado; Claudia Wong-Arámbula; Silvia Rivero-Arredondo; Ruth Madera-Sandoval; Joaquín Quiroz-Mercado; Fernando González-Domínguez; Lucia Hernández-Rivas, Irma López-Martínez; Ernesto Ramírez-González; Maribel González-Villa
EPI_ISL_17692268, EPI_ISL_17692269	Tokyo Metropolitan Institute of Public Health	Tokyo Metropolitan Institute of Public Health	Fumi Kasuya, Wakaba Okada, Ryota Kumagai, Sachiko Harada, Arisa Amano, Michiya Hasegawa, Mami Nagashima, Kenji Sadamasu
EPI_ISL_17703584	LESP Chihuahua	Instituto de Diagnostico y Referencia Epidemiologicos (INDRE)	Abril Rodríguez-Maldonado; Claudia Wong-Arámbula; Felipe Arguijo-Perez; Helios Cárdenas-Hernández; Carmen Castro-Méndez; Lidia García-Torres; Ruth Madera-Sandoval; América Mandujano-Martínez; Nancy Martínez-Velázquez; Mireya Mederos-Michel; Angélica Pedraza-Meléndez; Joaquín Quiroz-Mercado; Daniel Regalado-Santiago; Silvia Rivero-Arredondo; Erika Sierra-Atanacio; Fernando González-Domínguez; Lucia Hernández-Rivas, Irma López-Martínez; Ernesto Ramírez-González; Maribel González-Villa
EPI_ISL_17703586, EPI_ISL_17703587	LESP Nuevo Leon	Instituto de Diagnostico y Referencia Epidemiologicos (INDRE)	Abril Rodríguez-Maldonado; Claudia Wong-Arámbula; Felipe Arguijo-Perez; Helios Cárdenas-Hernández; Carmen Castro-Méndez; Lidia García-Torres; Ruth Madera-Sandoval; América Mandujano-Martínez; Nancy Martínez-Velázquez; Mireya Mederos-Michel; Angélica Pedraza-Meléndez; Joaquín Quiroz-Mercado; Daniel Regalado-Santiago; Silvia Rivero-Arredondo; Erika Sierra-Atanacio; Fernando González-Domínguez; Lucia Hernández-Rivas, Irma López-Martínez; Ernesto Ramírez-González; Maribel González-Villa
EPI_ISL_17703588, EPI_ISL_17703589	LESP Quintana Roo	Instituto de Diagnostico y Referencia Epidemiologicos (INDRE)	Abril Rodríguez-Maldonado; Claudia Wong-Arámbula; Felipe Arguijo-Perez; Helios Cárdenas-Hernández; Carmen Castro-Méndez; Lidia García-Torres; Ruth Madera-Sandoval; América Mandujano-Martínez; Nancy Martínez-Velázquez; Mireya Mederos-Michel; Angélica Pedraza-Meléndez; Joaquín Quiroz-Mercado; Daniel Regalado-Santiago; Silvia Rivero-Arredondo; Erika Sierra-Atanacio; Fernando González-Domínguez; Lucia Hernández-Rivas, Irma López-Martínez; Ernesto Ramírez-González; Maribel González-Villa
EPI_ISL_17703590	LESP State of Mexico	Instituto de Diagnostico y Referencia Epidemiologicos (INDRE)	Abril Rodríguez-Maldonado; Claudia Wong-Arámbula; Felipe Arguijo-Perez; Helios Cárdenas-Hernández; Carmen Castro-Méndez; Lidia García-Torres; Ruth Madera-Sandoval; América Mandujano-Martínez; Nancy Martínez-Velázquez; Mireya Mederos-Michel; Angélica Pedraza-Meléndez; Joaquín Quiroz-Mercado; Daniel Regalado-Santiago; Silvia Rivero-Arredondo; Erika Sierra-Atanacio; Fernando González-Domínguez; Lucia Hernández-Rivas, Irma López-Martínez; Ernesto Ramírez-González; Maribel González-Villa
EPI_ISL_17703591	LESP Campeche	Instituto de Diagnostico y Referencia Epidemiologicos (INDRE)	Abril Rodríguez-Maldonado; Claudia Wong-Arámbula; Felipe Arguijo-Perez; Helios Cárdenas-Hernández; Carmen Castro-Méndez; Lidia García-Torres; Ruth Madera-Sandoval; América Mandujano-Martínez; Nancy Martínez-Velázquez; Mireya Mederos-Michel; Angélica Pedraza-Meléndez; Joaquín Quiroz-Mercado; Daniel Regalado-Santiago; Silvia Rivero-Arredondo; Erika Sierra-Atanacio; Fernando González-Domínguez; Lucia Hernández-Rivas, Irma López-Martínez; Ernesto Ramírez-González; Maribel González-Villa
EPI_ISL_17703592	LESP Nuevo Leon	Instituto de Diagnostico y Referencia Epidemiologicos (INDRE)	Abril Rodríguez-Maldonado; Claudia Wong-Arámbula; Felipe Arguijo-Perez; Helios Cárdenas-Hernández; Carmen Castro-Méndez; Lidia García-Torres; Ruth Madera-Sandoval; América Mandujano-Martínez; Nancy Martínez-Velázquez; Mireya Mederos-Michel; Angélica Pedraza-Meléndez; Joaquín Quiroz-Mercado; Daniel Regalado-Santiago; Silvia Rivero-Arredondo; Erika Sierra-Atanacio; Fernando González-Domínguez; Lucia Hernández-Rivas, Irma López-Martínez; Ernesto Ramírez-González; Maribel González-Villa
EPI_ISL_17703593, EPI_ISL_17703594	LESP Jalisco	Instituto de Diagnostico y Referencia Epidemiologicos (INDRE)	Abril Rodríguez-Maldonado; Claudia Wong-Arámbula; Felipe Arguijo-Perez; Helios Cárdenas-Hernández; Carmen Castro-Méndez; Lidia García-Torres; Ruth Madera-Sandoval; América Mandujano-Martínez; Nancy Martínez-Velázquez; Mireya Mederos-Michel; Angélica Pedraza-Meléndez; Joaquín Quiroz-Mercado; Daniel Regalado-Santiago; Silvia Rivero-Arredondo; Erika Sierra-Atanacio; Fernando González-Domínguez; Lucia Hernández-Rivas, Irma López-Martínez; Ernesto Ramírez-González; Maribel González-Villa
EPI_ISL_17703595, EPI_ISL_17703596	LESP Nuevo Leon	Instituto de Diagnostico y Referencia Epidemiologicos (INDRE)	Abril Rodríguez-Maldonado; Claudia Wong-Arámbula; Felipe Arguijo-Perez; Helios Cárdenas-Hernández; Carmen Castro-Méndez; Lidia García-Torres; Ruth Madera-Sandoval; América Mandujano-Martínez; Nancy Martínez-Velázquez; Mireya Mederos-Michel; Angélica Pedraza-Meléndez; Joaquín Quiroz-Mercado; Daniel Regalado-Santiago; Silvia Rivero-Arredondo; Erika Sierra-Atanacio; Fernando González-Domínguez; Lucia Hernández-Rivas, Irma López-Martínez; Ernesto Ramírez-González; Maribel González-Villa
EPI_ISL_17703597	Laboratorio Estatal de Salud Pública Nuevo Leon	Instituto de Diagnostico y Referencia Epidemiologicos (INDRE)	Abril Rodríguez-Maldonado; Claudia Wong-Arámbula; Felipe Arguijo-Perez; Helios Cárdenas-Hernández; Carmen Castro-Méndez; Lidia García-Torres; Ruth Madera-Sandoval; América Mandujano-Martínez; Nancy Martínez-Velázquez; Mireya Mederos-Michel; Angélica Pedraza-Meléndez; Joaquín Quiroz-Mercado; Daniel Regalado-Santiago; Silvia Rivero-Arredondo; Erika Sierra-Atanacio; Fernando González-Domínguez; Lucia Hernández-Rivas, Irma López-Martínez; Ernesto Ramírez-González; Maribel González-Villa
EPI_ISL_17703598	LESP Quintana Roo	Instituto de Diagnostico y Referencia Epidemiologicos (INDRE)	Abril Rodríguez-Maldonado; Claudia Wong-Arámbula; Felipe Arguijo-Perez; Helios Cárdenas-Hernández; Carmen Castro-Méndez; Lidia García-Torres; Ruth Madera-Sandoval; América Mandujano-Martínez; Nancy Martínez-Velázquez; Mireya Mederos-Michel; Angélica Pedraza-Meléndez; Joaquín Quiroz-Mercado; Daniel Regalado-Santiago; Silvia Rivero-Arredondo; Erika Sierra-Atanacio; Fernando González-Domínguez; Lucia Hernández-Rivas, Irma López-Martínez; Ernesto Ramírez-González; Maribel González-Villa
EPI_ISL_17703599, EPI_ISL_17703600, EPI_ISL_17703601, EPI_ISL_17703602, EPI_ISL_17703603, EPI_ISL_17703604	LESP Nuevo Leon	Instituto de Diagnostico y Referencia Epidemiologicos (INDRE)	Abril Rodríguez-Maldonado; Claudia Wong-Arámbula; Felipe Arguijo-Perez; Helios Cárdenas-Hernández; Carmen Castro-Méndez; Lidia García-Torres; Ruth Madera-Sandoval; América Mandujano-Martínez; Nancy Martínez-Velázquez; Mireya Mederos-Michel; Angélica Pedraza-Meléndez; Joaquín Quiroz-Mercado; Daniel Regalado-Santiago; Silvia Rivero-Arredondo; Erika Sierra-Atanacio; Fernando González-Domínguez; Lucia Hernández-Rivas, Irma López-Martínez; Ernesto Ramírez-González; Maribel González-Villa
EPI_ISL_17703605	LESP Chihuahua	Instituto de Diagnostico y Referencia Epidemiologicos (INDRE)	Abril Rodríguez-Maldonado; Claudia Wong-Arámbula; Felipe Arguijo-Perez; Helios Cárdenas-Hernández; Carmen Castro-Méndez; Lidia García-Torres; Ruth Madera-Sandoval; América Mandujano-Martínez; Nancy Martínez-Velázquez; Mireya Mederos-Michel; Angélica Pedraza-Meléndez; Joaquín Quiroz-Mercado; Daniel Regalado-Santiago; Silvia Rivero-Arredondo; Erika Sierra-Atanacio; Fernando González-Domínguez; Lucia Hernández-Rivas, Irma López-Martínez; Ernesto Ramírez-González; Maribel González-Villa
EPI_ISL_17703606	LESP Nuevo Leon	Instituto de Diagnostico y Referencia Epidemiologicos (INDRE)	Abril Rodríguez-Maldonado; Claudia Wong-Arámbula; Felipe Arguijo-Perez; Helios Cárdenas-Hernández; Carmen Castro-Méndez; Lidia García-Torres; Ruth Madera-Sandoval; América Mandujano-Martínez; Nancy Martínez-Velázquez; Mireya Mederos-Michel; Angélica Pedraza-Meléndez; Joaquín Quiroz-Mercado; Daniel Regalado-Santiago; Silvia Rivero-Arredondo; Erika Sierra-Atanacio; Fernando González-Domínguez; Lucia Hernández-Rivas, Irma López-Martínez; Ernesto Ramírez-González; Maribel González-Villa
EPI_ISL_17703607, EPI_ISL_17703608, EPI_ISL_17703609, EPI_ISL_17703610	LESP Mexico City	Instituto de Diagnostico y Referencia Epidemiologicos (INDRE)	Abril Rodríguez-Maldonado; Claudia Wong-Arámbula; Felipe Arguijo-Perez; Helios Cárdenas-Hernández; Carmen Castro-Méndez; Lidia García-Torres; Ruth Madera-Sandoval; América Mandujano-Martínez; Nancy Martínez-Velázquez; Mireya Mederos-Michel; Angélica Pedraza-Meléndez; Joaquín Quiroz-Mercado; Daniel Regalado-Santiago; Silvia Rivero-Arredondo; Erika Sierra-Atanacio; Fernando González-Domínguez; Lucia Hernández-Rivas, Irma López-Martínez; Ernesto Ramírez-González; Maribel González-Villa
EPI_ISL_17703611	LESP Tlaxcala	Instituto de Diagnostico y Referencia Epidemiologicos (INDRE)	Abril Rodríguez-Maldonado; Claudia Wong-Arámbula; Felipe Arguijo-Perez; Helios Cárdenas-Hernández; Carmen Castro-Méndez; Lidia García-Torres; Ruth Madera-Sandoval; América Mandujano-Martínez; Nancy Martínez-Velázquez; Mireya Mederos-Michel; Angélica Pedraza-Meléndez; Joaquín Quiroz-Mercado; Daniel Regalado-Santiago; Silvia Rivero-Arredondo; Erika Sierra-Atanacio; Fernando González-Domínguez; Lucia Hernández-Rivas, Irma López-Martínez; Ernesto Ramírez-González; Maribel González-Villa
EPI_ISL_17703612	LESP Nuevo Leon	Instituto de Diagnostico y Referencia Epidemiologicos (INDRE)	Abril Rodríguez-Maldonado; Claudia Wong-Arámbula; Felipe Arguijo-Perez; Helios Cárdenas-Hernández; Carmen Castro-Méndez; Lidia García-Torres; Ruth Madera-Sandoval; América Mandujano-Martínez; Nancy Martínez-Velázquez; Mireya Mederos-Michel; Angélica Pedraza-Meléndez; Joaquín Quiroz-Mercado; Daniel Regalado-Santiago; Silvia Rivero-Arredondo; Erika Sierra-Atanacio; Fernando González-Domínguez; Lucia Hernández-Rivas, Irma López-Martínez; Ernesto Ramírez-González; Maribel González-Villa
EPI_ISL_17718458	Department of Virology, National Institute of Health, Islamabad, Pakistan	Department of Virology, National Institute of Health, Islamabad, Pakistan	Massab Umair, Muhammad Ammar, Syed Adnan Haider, Rabia Hakim, Qasim Malik, Muhammad Salman, Ghazala Parveen, and Naseem Akhtar
EPI_ISL_17722468, EPI_ISL_17762484, EPI_ISL_17762485	Tokyo Metropolitan Institute of Public Health	Tokyo Metropolitan Institute of Public Health	Fumi Kasuya, Wakaba Okada, Ryota Kumagai, Sachiko Harada, Arisa Amano, Michiya Hasegawa, Mami Nagashima, Kenji Sadamasu
EPI_ISL_17779993	Servicio de Microbiologia Hospital Ramon y Cajal	Servicio de Microbiologia Hospital Ramon y Cajal	Ponce-Alonso M, Martinez-García L, Olavarrieta L, Galan JC
EPI_ISL_17789831	Delaware Public Health Lab	Delaware Public Health Lab	Moneeb Bajwa & Holly Miller
EPI_ISL_17804477	Center for Virology, Medical University of Vienna	Center for Virology, Medical University of Vienna	Camp,J.V., Redlberger-Fritz,M. and Aberle,S.W.

EPI_ISL_17809521	Hangzhou Center for Disease Control and Prevention	Hangzhou Center for Disease Control and Prevention	Lijiao Ao , Jun Li , Yue Yu
EPI_ISL_17817239, EPI_ISL_17817240, EPI_ISL_17817241	Tokyo Metropolitan Institute of Public Health	Tokyo Metropolitan Institute of Public Health	Fumi Kasuya, Wakaba Okada, Ryota Kumagai, Sachiko Harada, Arisa Amano, Michiya Hasegawa, Mami Nagashima, Kenji Sadamasu
EPI_ISL_17821083, EPI_ISL_17821084, EPI_ISL_17821085, EPI_ISL_17821086, EPI_ISL_17821087, EPI_ISL_17821088	National Institute for Infectious Diseases "Matei Bals"	National Institute for Infectious Diseases "Matei Bals"	Robert Hohan, Ovidiu Vlaicu, Marius Surleac, Leontina Banica, Andreea Tudor, Simona Paraschiv
EPI_ISL_17821096, EPI_ISL_17821097, EPI_ISL_17821098	ACL Laboratories	RIPHL at Rush University Medical Center	Stefan Green, Kevin Kunstman, Hannah Barbian, Felix Araujo Perez, Edith Perez, Sofiya Bobrovska, Alyse Kittner, Cecilia Chau, Giancarlo Balangue, Lok Yiu Ashley Wu
EPI_ISL_17821099, EPI_ISL_17821100, EPI_ISL_17821101	Quest Diagnostics	RIPHL at Rush University Medical Center	Stefan Green, Kevin Kunstman, Hannah Barbian, Felix Araujo Perez, Edith Perez, Sofiya Bobrovska, Alyse Kittner, Cecilia Chau, Giancarlo Balangue, Lok Yiu Ashley Wu
EPI_ISL_17831608	Delaware Public Health Lab	Delaware Public Health Lab	Miller,H. and Bajwa,M.
EPI_ISL_17834476	California Department of Public Health	California Department of Public Health	Kath, C., Haw, M., Espinosa, A., and Hacker, J.
EPI_ISL_17837266, EPI_ISL_17837267, EPI_ISL_17837268, EPI_ISL_17959214, EPI_ISL_17959215, EPI_ISL_17959216	Tokyo Metropolitan Institute of Public Health	Tokyo Metropolitan Institute of Public Health	Fumi Kasuya, Wakaba Okada, Ryota Kumagai, Sachiko Harada, Arisa Amano, Michiya Hasegawa, Mami Nagashima, Kenji Sadamasu
EPI_ISL_17960863, EPI_ISL_17960864, EPI_ISL_17960866	Laboratorio Nacional de Salud Pública Dr. Defilló	Laboratorio Nacional de Salud Pública Dr. Defilló	Isaac Miguel Sánchez, Carlos Vergara Castillo, Edwin Féliz, Anny Peña, Pedro Martinez, Yeny E. Lara Perez, Robinson Agramonte
EPI_ISL_17972012, EPI_ISL_17972014, EPI_ISL_17972015	California Department of Public Health (CDPH)	California Department of Public Health (CDPH)	Kath,C., Haw,M., Espinosa,A. and Hacker,J.
EPI_ISL_17979000, EPI_ISL_17979001, EPI_ISL_17979002	Quest Diagnostics	Regional Innovative Public Health Laboratory at Rush University Medical Center	Stefan Green, Kevin Kunstman, Hannah Barbian, Sofiya Bobrovska, Felix Araujo Perez, Edith Perez, Cecilia Chau, Giancarlo Balangue, Lok Yiu Ashley Wu, Trisha Jeon, Marisol Dominguez, Latifah Boyd
EPI_ISL_17980807	Hospital Ramon y Cajal	Hospital Ramon y Cajal	Ponce-Alonso,M., Martinez-Garcia,L., Olavarrieta,L. and Galan,J.C.
EPI_ISL_17988349, EPI_ISL_17988350, EPI_ISL_17988351, EPI_ISL_17988352, EPI_ISL_17988353, EPI_ISL_17988354, EPI_ISL_17988355, EPI_ISL_17988356, EPI_ISL_17988357, EPI_ISL_17988358, EPI_ISL_17988359, EPI_ISL_17988360, EPI_ISL_17988361, EPI_ISL_17988374, EPI_ISL_17988375, EPI_ISL_17988376, EPI_ISL_17988377, EPI_ISL_17988378			
see above	Laboratorio Central de Salud Publica	Laboratorio Central de Salud Publica	Cynthia Vazquez, Vagner Fonseca, Andrea Gomez de la Fuente, Sandra Gonzalez, Fatima Fleitas, Mauricio Lima, Natalia R. Guimaraes, Felipe C. M. Iani, Analia Rojas, Tania Alfonso, Cesar Cantero, Julio Barrios, Shirley Villalba, Maria Jose Ortega, Juan Torales, Maria Liz Gamarra, Carolina Aquino, Jairo Mendez Rico, Luiz Carlos Junior Alcantara, Marta Giovanetti
EPI_ISL_18044981, EPI_ISL_18044982, EPI_ISL_18044983, EPI_ISL_18044984, EPI_ISL_18044985, EPI_ISL_18044986, EPI_ISL_18044987	Center for Vectors and Infectious Diseases Research (CEVDI), National Health Institute Doutor Ricardo Jorge, IP (INSA),	Center for Vectors and Infectious Diseases Research (CEVDI), National Health Institute Doutor Ricardo Jorge, IP (INSA),	Isidro,J., Borges,V., Pinto,M., Sobral,D., Santos,J., Nunes,A., Mixao,V., Ferreira,R., Santos,D., Duarte,S., Vieira,L., Borrego,M.J., Nuncio,S., Lopes de Carvalho,I., Pelerito,A., Cordeiro,R. and Gomes,J.P.
EPI_ISL_18055899, EPI_ISL_18055900	Tokyo Metropolitan Institute of Public Health	Tokyo Metropolitan Institute of Public Health	Fumi Kasuya, Wakaba Okada, Ryota Kumagai, Sachiko Harada, Arisa Amano, Michiya Hasegawa, Mami Nagashima, Kenji Sadamasu
EPI_ISL_18059182, EPI_ISL_18059183, EPI_ISL_18059184	Department of Acute Infectious Diseases Control and Prevention, Yunnan Center for Disease Control and Prevention	Department of Acute Infectious Diseases Control and Prevention, Yunnan Center for Disease Control and Prevention	Meiling Zhang, Ruize Ni, Xiaoqing Fu
EPI_ISL_18075506, EPI_ISL_18075507, EPI_ISL_18075508	Tokyo Metropolitan Institute of Public Health	Tokyo Metropolitan Institute of Public Health	Fumi Kasuya, Wakaba Okada, Ryota Kumagai, Sachiko Harada, Arisa Amano, Michiya Hasegawa, Mami Nagashima, Kenji Sadamasu
EPI_ISL_18076378, EPI_ISL_18076379, EPI_ISL_18076380, EPI_ISL_18076381, EPI_ISL_18076382, EPI_ISL_18076383, EPI_ISL_18076384, EPI_ISL_18076385, EPI_ISL_18076386, EPI_ISL_18076387, EPI_ISL_18076388, EPI_ISL_18076389			
see above	Center for Vectors and Infectious Diseases Research (CEVDI), National Health Institute Doutor Ricardo Jorge, IP (INSA)	Center for Vectors and Infectious Diseases Research (CEVDI), National Health Institute Doutor Ricardo Jorge, IP (INSA)	Isidro,J., Borges,V., Pinto,M., Sobral,D., Santos,J., Nunes,A., Mixao,V., Ferreira,R., Santos,D., Duarte,S., Vieira,L., Borrego,M.J., Nuncio,S., Lopes de Carvalho,I., Pelerito,A., Cordeiro,R. and Gomes,J.P.
EPI_ISL_18097375	Tokyo Metropolitan Institute of Public Health	Tokyo Metropolitan Institute of Public Health	Fumi Kasuya, Wakaba Okada, Ryota Kumagai, Sachiko Harada, Arisa Amano, Michiya Hasegawa, Mami Nagashima, Kenji Sadamasu
EPI_ISL_18125028, EPI_ISL_18125035, EPI_ISL_18125036, EPI_ISL_18125037, EPI_ISL_18125038, EPI_ISL_18125039, EPI_ISL_18125040, EPI_ISL_18125044	University of Washington, Department of Laboratory Medicine	University of Washington, Department of Laboratory Medicine	Sereewit,J., Xie,H., Roychoudhury,P. and Greninger,A.L.
EPI_ISL_18131392, EPI_ISL_18131393	Robert Koch Institute	Robert Koch Institute	Brinkmann,A., Pape,K., Kohl,C., Schrick,L., Michel,J., Schaade,L. and Nitsche,A.
EPI_ISL_18137801, EPI_ISL_18137802, EPI_ISL_18137803	Northwestern Medicine	RIPHL at Rush University Medical Center	Stefan Green, Kevin Kunstman, Hannah Barbian, Sofiya Bobrovska, Felix Araujo Perez, Edith Perez, Cecilia Chau, Giancarlo Balangue, Lok Yiu Ashley Wu, Trisha Jeon, Marisol Dominguez, Latifah Boyd, Lacy Simons
EPI_ISL_18137804	Quest Diagnostics	RIPHL at Rush University Medical Center	Stefan Green, Kevin Kunstman, Hannah Barbian, Sofiya Bobrovska, Felix Araujo Perez, Edith Perez, Cecilia Chau, Giancarlo Balangue, Lok Yiu Ashley Wu, Trisha Jeon, Marisol Dominguez, Latifah Boyd
EPI_ISL_18137805	ACL Laboratories	RIPHL at Rush University Medical Center	Stefan Green, Kevin Kunstman, Hannah Barbian, Sofiya Bobrovska, Felix Araujo Perez, Edith Perez, Cecilia Chau, Giancarlo Balangue, Lok Yiu Ashley Wu, Trisha Jeon, Marisol Dominguez, Latifah Boyd
EPI_ISL_18137806	Quest Diagnostics	RIPHL at Rush University Medical Center	Stefan Green, Kevin Kunstman, Hannah Barbian, Sofiya Bobrovska, Felix Araujo Perez, Edith Perez, Cecilia Chau, Giancarlo Balangue, Lok Yiu Ashley Wu, Trisha Jeon, Marisol Dominguez, Latifah Boyd
EPI_ISL_18137807, EPI_ISL_18137808	Northwestern Medicine	RIPHL at Rush University Medical Center	Stefan Green, Kevin Kunstman, Hannah Barbian, Sofiya Bobrovska, Felix Araujo Perez, Edith Perez, Cecilia Chau, Giancarlo Balangue, Lok Yiu Ashley Wu, Trisha Jeon, Marisol Dominguez, Latifah Boyd, Lacy Simons
EPI_ISL_18137809, EPI_ISL_18137810, EPI_ISL_18137811, EPI_ISL_18137812, EPI_ISL_18137813	Quest Diagnostics	RIPHL at Rush University Medical Center	Stefan Green, Kevin Kunstman, Hannah Barbian, Sofiya Bobrovska, Felix Araujo Perez, Edith Perez, Cecilia Chau, Giancarlo Balangue, Lok Yiu Ashley Wu, Trisha Jeon, Marisol Dominguez, Latifah Boyd
EPI_ISL_18137814, EPI_ISL_18137815, EPI_ISL_18137816, EPI_ISL_18137817, EPI_ISL_18137818, EPI_ISL_18137819, EPI_ISL_18137820	Northwestern Medicine	RIPHL at Rush University Medical Center	Stefan Green, Kevin Kunstman, Hannah Barbian, Sofiya Bobrovska, Felix Araujo Perez, Edith Perez, Cecilia Chau, Giancarlo Balangue, Lok Yiu Ashley Wu, Trisha Jeon, Marisol Dominguez, Latifah Boyd, Lacy Simons
EPI_ISL_18137830	University of Washington, Department of Laboratory Medicine	University of Washington, Department of Laboratory Medicine	Sereewit,J., Xie,H., Roychoudhury,P. and Greninger,A.L.
EPI_ISL_18147334, EPI_ISL_18147336, EPI_ISL_18147337, EPI_ISL_18147338, EPI_ISL_18147339, EPI_ISL_18147340, EPI_ISL_18147341, EPI_ISL_18147342, EPI_ISL_18147343, EPI_ISL_18147344, EPI_ISL_18147345, EPI_ISL_18147346, EPI_ISL_18147347, EPI_ISL_18147348, EPI_ISL_18147349, EPI_ISL_18147350, EPI_ISL_18147351, EPI_ISL_18147352, EPI_ISL_18147353, EPI_ISL_18147355, EPI_ISL_18147356, EPI_ISL_18147358, EPI_ISL_18147359, EPI_ISL_18147360, EPI_ISL_18147361, EPI_ISL_18147362, EPI_ISL_18147363			
see above	Korea Disease Control and Prevention Agency	Korea Disease Control and Prevention Agency	Chung,Y.-S., Yi,H., Choi,M.-M., Kim,J.-W., Lee,M., Lee,S., Sim,G., Lee,J.H., Shin,H. and Choi,C.
EPI_ISL_18161268, EPI_ISL_18161269, EPI_ISL_18161270, EPI_ISL_18161271, EPI_ISL_18161272, EPI_ISL_18161273, EPI_ISL_18161274, EPI_ISL_18161275, EPI_ISL_18161276, EPI_ISL_18161277, EPI_ISL_18161278, EPI_ISL_18161279			
see above	Quest Diagnostics Nichols Institute	Los Angeles County Public Health Laboratories	J. Garrigues et al.
EPI_ISL_18161280	UCLA Clinical Micro Lab	Los Angeles County Public Health Laboratories	J. Garrigues et al.
EPI_ISL_18161281, EPI_ISL_18161282, EPI_ISL_18161283, EPI_ISL_18161284, EPI_ISL_18161285, EPI_ISL_18161286	Quest Diagnostics Nichols Institute	Los Angeles County Public Health Laboratories	J. Garrigues et al.
EPI_ISL_18161287, EPI_ISL_18161288, EPI_ISL_18161289, EPI_ISL_18161290, EPI_ISL_18161291	Los Angeles County Public Health Laboratories	Los Angeles County Public Health Laboratories	J. Garrigues et al.
EPI_ISL_18161292	Quest Diagnostics Nichols Institute	Los Angeles County Public Health Laboratories	J. Garrigues et al.
EPI_ISL_18161293, EPI_ISL_18161294, EPI_ISL_18161295, EPI_ISL_18161296	Laboratory Corporation of America	Los Angeles County Public Health Laboratories	J. Garrigues et al.
EPI_ISL_18161297, EPI_ISL_18161298, EPI_ISL_18161299, EPI_ISL_18161300, EPI_ISL_18161301, EPI_ISL_18161302, EPI_ISL_18161303, EPI_ISL_18161304, EPI_ISL_18161305	Quest Diagnostics Nichols Institute	Los Angeles County Public Health Laboratories	J. Garrigues et al.
EPI_ISL_18161306	Los Angeles County Public Health Laboratories	Los Angeles County Public Health Laboratories	J. Garrigues et al.
EPI_ISL_18161307, EPI_ISL_18161308, EPI_ISL_18161309, EPI_ISL_18161310, EPI_ISL_18161311, EPI_ISL_18161312, EPI_ISL_18161313, EPI_ISL_18161314	Quest Diagnostics Nichols Institute	Los Angeles County Public Health Laboratories	J. Garrigues et al.
EPI_ISL_18161315	ARUP Laboratories	Los Angeles County Public Health Laboratories	J. Garrigues et al.
EPI_ISL_18161316, EPI_ISL_18161317, EPI_ISL_18161318, EPI_ISL_18161319, EPI_ISL_18161320, EPI_ISL_18161321, EPI_ISL_18161322, EPI_ISL_18161323, EPI_ISL_18161324	Laboratory Corporation of America	Los Angeles County Public Health Laboratories	J. Garrigues et al.
EPI_ISL_18161325	Los Angeles County Public Health Laboratories	Los Angeles County Public Health Laboratories	J. Garrigues et al.
EPI_ISL_18161326	Quest Diagnostics Nichols Institute	Los Angeles County Public Health Laboratories	J. Garrigues et al.
EPI_ISL_18168621, EPI_ISL_18168622, EPI_ISL_18168623	Quest Diagnostics	RIPHL at Rush University Medical Center	Stefan Green, Kevin Kunstman, Hannah Barbian, Sofiya Bobrovska, Felix Araujo Perez, Edith Perez, Cecilia Chau, Giancarlo Balangue, Lok Yiu Ashley Wu, Trisha Jeon, Marisol Dominguez, Latifah Boyd
EPI_ISL_18168624	ACL Laboratories	RIPHL at Rush University Medical Center	Stefan Green, Kevin Kunstman, Hannah Barbian, Sofiya Bobrovska, Felix Araujo Perez, Edith Perez, Cecilia Chau, Giancarlo Balangue, Lok Yiu Ashley Wu, Trisha Jeon, Marisol Dominguez, Latifah Boyd
EPI_ISL_18213374, EPI_ISL_18213375	Institute for Hepatology,Shenzhen Third People's Hospital	Institute for Hepatology,Shenzhen Third People's Hospital	Lin Cheng,Zheng Zhang
EPI_ISL_18228619, EPI_ISL_18228620, EPI_ISL_18228621, EPI_ISL_18228622, EPI_ISL_18228623, EPI_ISL_18228624, EPI_ISL_18228626, EPI_ISL_18228627, EPI_ISL_18228628, EPI_ISL_18228629, EPI_ISL_18228630, EPI_ISL_18228631, EPI_ISL_18228633, EPI_ISL_18228634, EPI_ISL_18228635, EPI_ISL_18228636, EPI_ISL_18228637, EPI_ISL_18228638, EPI_ISL_18228639, EPI_ISL_18228640, EPI_ISL_18228641, EPI_ISL_18228642, EPI_ISL_18228643, EPI_ISL_18228644, EPI_ISL_18228645			
see above	Center for Vectors and Infectious Diseases Research (CEVDI), National Health Institute Doutor Ricardo Jorge, IP (INSA)	Center for Vectors and Infectious Diseases Research (CEVDI), National Health Institute Doutor Ricardo Jorge, IP (INSA)	Isidro,J., Borges,V., Pinto,M., Sobral,D., Santos,J., Nunes,A., Mixao,V., Ferreira,R., Santos,D., Duarte,S., Vieira,L., Borrego,M.J., Nuncio,S., Lopes de Carvalho,I., Pelerito,A., Cordeiro,R. and Gomes,J.P.
EPI_ISL_18257122	Haidian CDC	Haidian District Center for Disease Control and Prevention Microbiological Laboratory	Fangyao Liu, Lifei Shi,Feng Liu, Heng Zhang
EPI_ISL_18285958	Laboratory Corporation of America	Los Angeles County Public Health Laboratories	J. Garrigues et al.

EPI_ISL_18285959	UCLA Clinical Micro Lab	Los Angeles County Public Health Laboratories	J. Garrigues et al.
EPI_ISL_18285960, EPI_ISL_18285961, EPI_ISL_18285962, EPI_ISL_18285963, EPI_ISL_18285964, EPI_ISL_18285965, EPI_ISL_18285966, EPI_ISL_18285967, EPI_ISL_18285968	Quest Diagnostics Nichols Institute	Los Angeles County Public Health Laboratories	J. Garrigues et al.
EPI_ISL_18285969	Laboratory Corporation of America	Los Angeles County Public Health Laboratories	J. Garrigues et al.
EPI_ISL_18285970	Quest Diagnostics Nichols Institute	Los Angeles County Public Health Laboratories	J. Garrigues et al.
EPI_ISL_18285971, EPI_ISL_18285972	Cedars-Sinai Medical Center	Los Angeles County Public Health Laboratories	J. Garrigues et al.
EPI_ISL_18285973, EPI_ISL_18285974, EPI_ISL_18285975, EPI_ISL_18285976, EPI_ISL_18285977, EPI_ISL_18285978, EPI_ISL_18285979, EPI_ISL_18285980, EPI_ISL_18285981, EPI_ISL_18285982, EPI_ISL_18285983, EPI_ISL_18285984, EPI_ISL_18285985, EPI_ISL_18285986, EPI_ISL_18285987			
see above	Quest Diagnostics	RIPHL at Rush University Medical Center	Stefan Green, Kevin Kunstman, Hannah Barbian, Sofiya Bobrovska, Felix Araujo Perez, Edith Perez, Cecilia Chau, Giancarlo Balangue, Lok Yiu Ashley Wu, Trisha Jeon, Marisol Dominguez, Latifah Boyd
EPI_ISL_18299473, EPI_ISL_18299474	Centre for Biological Threats, Highly Pathogenic Viruses, Robert Koch Institute	Centre for Biological Threats, Highly Pathogenic Viruses, Robert Koch Institute	Brinkmann,A., Kohl,C., Schrick,L., Michel,J., Schaade,L. and Nitsche,A.
EPI_ISL_18308396, EPI_ISL_18308397, EPI_ISL_18308398, EPI_ISL_18308399	National Virus Reference Laboratory	National Virus Reference Laboratory	Gabriel Gonzalez, Michael Carr, Emer O'Byrne, Weronika Banka, Brian Keogan, Jonathan Dean, Daniel Hare, Cillian F De Gascun
EPI_ISL_18323785, EPI_ISL_18323786, EPI_ISL_18323787, EPI_ISL_18323788, EPI_ISL_18323789, EPI_ISL_18323790, EPI_ISL_18323791, EPI_ISL_18323792, EPI_ISL_18323793, EPI_ISL_18323794, EPI_ISL_18324980, EPI_ISL_18324981, EPI_ISL_18324994, EPI_ISL_18324995, EPI_ISL_18325000, EPI_ISL_18325001, EPI_ISL_18325002, EPI_ISL_18325003, EPI_ISL_18325004, EPI_ISL_18325005, EPI_ISL_18325006, EPI_ISL_18325007, EPI_ISL_18325008, EPI_ISL_18325009, EPI_ISL_18325010, EPI_ISL_18325011			
see above	California Department of Public Health	California Department of Public Health	Kath, C., Haw, M., Espinosa, A., and Hacker, J.
EPI_ISL_18352302, EPI_ISL_18352303, EPI_ISL_18352304, EPI_ISL_18352305, EPI_ISL_18352306	Tokyo Metropolitan Institute of Public Health	Tokyo Metropolitan Institute of Public Health	Fumi Kasuya, Wakaba Okada, Ryota Kumagai, Sachiko Harada, Arisa Amano, Michiya Hasegawa, Mami Nagashima, Kenji Sadamasu
EPI_ISL_18354483	Shenzhen Key Laboratory of Pathogen and Immunity	Shenzhen Key Laboratory of Pathogen and Immunity	Yang Yang, Shengjie Zhang, Yun Peng, Fuxiang Wang, Yingxia Liu, Hongzhou Lu
EPI_ISL_18360394	Haidian District Center for Disease Control and Prevention Microbiological Laboratory	Haidian District Center for Disease Control and Prevention Microbiological Laboratory	Fangyao Liu, Lifei Shi,Feng Liu, Heng Zhang
EPI_ISL_18386999, EPI_ISL_18387000, EPI_ISL_18387001, EPI_ISL_18387002, EPI_ISL_18387003, EPI_ISL_18387004, EPI_ISL_18387005, EPI_ISL_18387006, EPI_ISL_18387007, EPI_ISL_18387008, EPI_ISL_18387009, EPI_ISL_18387010, EPI_ISL_18387011, EPI_ISL_18387012, EPI_ISL_18387013			
see above	NC - Los Angeles County Public Health Laboratories	NC - Los Angeles County Public Health Laboratories	Garrigues,J.M. and Green,N.M.
EPI_ISL_18399140, EPI_ISL_18399141, EPI_ISL_18399142, EPI_ISL_18399143, EPI_ISL_18399144, EPI_ISL_18399145, EPI_ISL_18399146, EPI_ISL_18399147	California Department of Public Health	California Department of Public Health	Kath, C., Haw, M., Espinosa, A., and Hacker, J.
EPI_ISL_18414668, EPI_ISL_18414669, EPI_ISL_18414670, EPI_ISL_18414671, EPI_ISL_18414672	Laboratory Medicine, UW Virology	Laboratory Medicine, UW Virology	Sereewit,J., Xie,H., Roychoudhury,P. and Greninger,A.L.
EPI_ISL_18427686, EPI_ISL_18427687, EPI_ISL_18427688, EPI_ISL_18427689, EPI_ISL_18427690, EPI_ISL_18427691	Quest Diagnostics	RIPHL at Rush University Medical Center	Stefan Green, Kevin Kunstman, Hannah Barbian, Sofiya Bobrovska, Felix Araujo Perez, Edith Perez, Cecilia Chau, Giancarlo Balangue, Lok Yiu Ashley Wu, Trisha Jeon, Marisol Dominguez, Latifah Boyd
EPI_ISL_18427692	ACL Laboratories	RIPHL at Rush University Medical Center	Stefan Green, Kevin Kunstman, Hannah Barbian, Sofiya Bobrovska, Felix Araujo Perez, Edith Perez, Cecilia Chau, Giancarlo Balangue, Lok Yiu Ashley Wu, Trisha Jeon, Marisol Dominguez, Latifah Boyd
EPI_ISL_18436040	PKC Mampang Prapatan	National Institute of Health Research and Development	Fajar Nur Sulistyahadi, Arie Ardiansyah Nugraha, Hana Apsari Pawestri, Kartika Dewi Puspa, Herna, Subangkit, IGM Wirabrata
EPI_ISL_18436041	PKC Jatinegara	National Institute of Health Research and Development	Fajar Nur Sulistyahadi, Arie Ardiansyah Nugraha, Hana Apsari Pawestri, Kartika Dewi Puspa, Herna, Subangkit, IGM Wirabrata
EPI_ISL_18443042, EPI_ISL_18443043, EPI_ISL_18443044, EPI_ISL_18452332, EPI_ISL_18452333, EPI_ISL_18452334, EPI_ISL_18452335, EPI_ISL_18452336, EPI_ISL_18452337, EPI_ISL_18452338, EPI_ISL_18452339, EPI_ISL_18452340, EPI_ISL_18452341, EPI_ISL_18452342, EPI_ISL_18452343, EPI_ISL_18452344, EPI_ISL_18452345, EPI_ISL_18452346, EPI_ISL_18452347, EPI_ISL_18458948, EPI_ISL_18458949, EPI_ISL_18458962, EPI_ISL_18458963, EPI_ISL_18458964, EPI_ISL_18460503, EPI_ISL_18460504, EPI_ISL_18460505			
see above	California Department of Public Health	California Department of Public Health	Kath, C., Haw, M., Espinosa, A., and Hacker, J.
EPI_ISL_18463158	PKM Kembangan	National Institute of Health Research and Development	Hana Apsari Pawestri, Arie Ardiansyah Nugraha, Fajar Nur Sulistyahadi, Hartanti Dian Ikawati, Kartika Dewi Puspa, Markus Evan Anggia, Subangkit, Nelis Imaningsih, IGM Wirabrata
EPI_ISL_18463159	PKC Setiabudi	National Institute of Health Research and Development	Hana Apsari Pawestri, Arie Ardiansyah Nugraha, Fajar Nur Sulistyahadi, Hartanti Dian Ikawati, Kartika Dewi Puspa, Markus Evan Anggia, Subangkit, Nelis Imaningsih, IGM Wirabrata
EPI_ISL_18463160	RSUPN Dr Cipto Mangunkusumo	National Institute of Health Research and Development	Hana Apsari Pawestri, Arie Ardiansyah Nugraha, Fajar Nur Sulistyahadi, Hartanti Dian Ikawati, Kartika Dewi Puspa, Markus Evan Anggia, Subangkit, Nelis Imaningsih, IGM Wirabrata
EPI_ISL_18463161	RSUD Kembangan	National Institute of Health Research and Development	Hana Apsari Pawestri, Arie Ardiansyah Nugraha, Fajar Nur Sulistyahadi, Hartanti Dian Ikawati, Kartika Dewi Puspa, Markus Evan Anggia, Subangkit, Nelis Imaningsih, IGM Wirabrata
EPI_ISL_18467794	Eka Hospital BSD	National Institute of Health Research and Development	Fajar Nur Sulistyahadi, Hana Apsari Pawestri, Arie Ardiansyah Nugraha, Hartanti Dian Ikawati, Kartika Dewi Puspa, Subangkit, IGM Wirabrata
EPI_ISL_18467795, EPI_ISL_18467796	PKM Kembangan	National Institute of Health Research and Development	Hana Apsari Pawestri, Arie Ardiansyah Nugraha, Fajar Nur Sulistyahadi, Hartanti Dian Ikawati, Kartika Dewi Puspa, Subangkit, IGM Wirabrata
EPI_ISL_18467797	PKC Cengkareng	National Institute of Health Research and Development	Arie Ardiansyah Nugraha, Fajar Nur Sulistyahadi, Hartanti Dian Ikawati, Kartika Dewi Puspa, Hana Apsari Pawestri, Subangkit, IGM Wirabrata
EPI_ISL_18467798	PKC Grogol Petamburan	National Institute of Health Research and Development	Fajar Nur Sulistyahadi, Hana Apsari Pawestri, Arie Ardiansyah Nugraha, Hartanti Dian Ikawati, Kartika Dewi Puspa, Subangkit, IGM Wirabrata
EPI_ISL_18467799	PKC Setiabudi	National Institute of Health Research and Development	Hana Apsari Pawestri, Arie Ardiansyah Nugraha, Fajar Nur Sulistyahadi, Hartanti Dian Ikawati, Kartika Dewi Puspa, Subangkit, IGM Wirabrata
EPI_ISL_18467800	PKC Pancoran	National Institute of Health Research and Development	Arie Ardiansyah Nugraha, Fajar Nur Sulistyahadi, Hartanti Dian Ikawati, Kartika Dewi Puspa, Hana Apsari Pawestri, Subangkit, IGM Wirabrata
EPI_ISL_18467801	PKM Mampang Prapatan	National Institute of Health Research and Development	Arie Ardiansyah Nugraha, Fajar Nur Sulistyahadi, Hartanti Dian Ikawati, Kartika Dewi Puspa, Hana Apsari Pawestri, Subangkit, IGM Wirabrata
EPI_ISL_18467802	PKC Cilandak	National Institute of Health Research and Development	Fajar Nur Sulistyahadi, Hana Apsari Pawestri, Arie Ardiansyah Nugraha, Hartanti Dian Ikawati, Kartika Dewi Puspa, Subangkit, IGM Wirabrata
EPI_ISL_18467803	RS Brawijaya Saharjo	National Institute of Health Research and Development	Fajar Nur Sulistyahadi, Hana Apsari Pawestri, Arie Ardiansyah Nugraha, Hartanti Dian Ikawati, Kartika Dewi Puspa, Subangkit, IGM Wirabrata
EPI_ISL_18467804	PKC Pulogadung	National Institute of Health Research and Development	Hana Apsari Pawestri, Arie Ardiansyah Nugraha, Fajar Nur Sulistyahadi, Hartanti Dian Ikawati, Kartika Dewi Puspa, Subangkit, IGM Wirabrata
EPI_ISL_18467805	PKC Kramat Jati	National Institute of Health Research and Development	Arie Ardiansyah Nugraha, Fajar Nur Sulistyahadi, Hartanti Dian Ikawati, Kartika Dewi Puspa, Hana Apsari Pawestri, Subangkit, IGM Wirabrata
EPI_ISL_18467806	PKM Tanjung Priuk	National Institute of Health Research and Development	Fajar Nur Sulistyahadi, Hana Apsari Pawestri, Arie Ardiansyah Nugraha, Hartanti Dian Ikawati, Kartika Dewi Puspa, Subangkit, IGM Wirabrata
EPI_ISL_18467807	PKC Kelapa Gading	National Institute of Health Research and Development	Fajar Nur Sulistyahadi, Hana Apsari Pawestri, Arie Ardiansyah Nugraha, Hartanti Dian Ikawati, Kartika Dewi Puspa, Subangkit, IGM Wirabrata
EPI_ISL_18467808	RSUP Dr Hasan Sadikin	National Institute of Health Research and Development	Arie Ardiansyah Nugraha, Fajar Nur Sulistyahadi, Hartanti Dian Ikawati, Kartika Dewi Puspa, Hana Apsari Pawestri, Subangkit, IGM Wirabrata
EPI_ISL_18486433, EPI_ISL_18486434, EPI_ISL_18486435, EPI_ISL_18486436, EPI_ISL_18486437, EPI_ISL_18486438, EPI_ISL_18486439, EPI_ISL_18486440, EPI_ISL_18538995, EPI_ISL_18538996, EPI_ISL_18538997, EPI_ISL_18538998, EPI_ISL_18538999, EPI_ISL_18539000, EPI_ISL_18539001, EPI_ISL_18539002, EPI_ISL_18539003, EPI_ISL_18539004, EPI_ISL_18539005, EPI_ISL_18539006			
see above	Centre for Biological Threats, Highly Pathogenic Viruses, Robert Koch Institute	Centre for Biological Threats, Highly Pathogenic Viruses, Robert Koch Institute	Brinkmann,A., Kohl,C., Schrick,L., Michel,J., Schaade,L. and Nitsche,A.
EPI_ISL_18539007, EPI_ISL_18539008, EPI_ISL_18539009, EPI_ISL_18539010, EPI_ISL_18539011, EPI_ISL_18539012, EPI_ISL_18539013, EPI_ISL_18539014, EPI_ISL_18539015, EPI_ISL_18539016, EPI_ISL_18539017			
see above	University of Washington, Department of Laboratory Medicine	University of Washington, Department of Laboratory Medicine	Sereewit,J., Nunley,E.B., Xie,H., Roychoudhury,P. and Greninger,A.L.
EPI_ISL_18553811, EPI_ISL_18553812, EPI_ISL_18553813, EPI_ISL_18553814, EPI_ISL_18553815, EPI_ISL_18553816, EPI_ISL_18553817, EPI_ISL_18553818, EPI_ISL_18553819, EPI_ISL_18553820, EPI_ISL_18553821, EPI_ISL_18553822, EPI_ISL_18553823, EPI_ISL_18553824, EPI_ISL_18553825, EPI_ISL_18553826, EPI_ISL_18553827, EPI_ISL_18553828, EPI_ISL_18553829, EPI_ISL_18553830, EPI_ISL_18553831, EPI_ISL_18553833, EPI_ISL_18553834, EPI_ISL_18553835, EPI_ISL_18553836, EPI_ISL_18553837, EPI_ISL_18553838, EPI_ISL_18553839, EPI_ISL_18553840, EPI_ISL_18553841, EPI_ISL_18553842, EPI_ISL_18553843, EPI_ISL_18553844, EPI_ISL_18553845, EPI_ISL_18553846, EPI_ISL_18553847, EPI_ISL_18553848, EPI_ISL_18553849, EPI_ISL_18553850			
see above	Center for Vectors and Infectious Diseases Research (CEVDI), National Health Institute Doutor Ricardo Jorge, IP (INSA)	Center for Vectors and Infectious Diseases Research (CEVDI), National Health Institute Doutor Ricardo Jorge, IP (INSA)	Isidro,J., Borges,V., Pinto,M., Sobral,D., Santos,J., Nunes,A., Mixao,V., Ferreira,R., Santos,D., Duarte,S., Vieira,L., Borrego,M.J., Nuncio,S., Lopes de Carvalho,I., Pelerito,A., Cordeiro,R. and Gomes,J.P.
EPI_ISL_18557816, EPI_ISL_18557817, EPI_ISL_18557818	Tokyo Metropolitan Institute of Public Health	Tokyo Metropolitan Institute of Public Health	Fumi Kasuya, Wakaba Okada, Ryota Kumagai, Sachiko Harada, Arisa Amano, Michiya Hasegawa, Mami Nagashima, Kenji Sadamasu
EPI_ISL_18560904, EPI_ISL_18560905	Department of Pediatrics, Faculty of Medicine, Chulalongkorn University	Department of Pediatrics, Faculty of Medicine, Chulalongkorn University	Puenpa,J., Vongpunsawad,S., Intharasongkroh,D., Chaiwanichsiri,D. and Poovorawan,Y.
EPI_ISL_18567805, EPI_ISL_18567806, EPI_ISL_18567807	Southern Nevada Public Health Laboratory	Southern Nevada Public Health Laboratory	Horng-Yuan Kan
EPI_ISL_18627266	Division of Infectious Diseases and Tropical Medicine, University Hospital, Ludwig-Maximilians-Universitaet (LMU) Munich, Munich, Germany	Bundeswehr Institute of Microbiology	MH Antwerpen, D Lang, S Zange, R Woelfel
EPI_ISL_18634755, EPI_ISL_18634756	National Virus Reference Laboratory	National Virus Reference Laboratory	Gabriel Gonzalez, Michael Carr, Emer O'Byrne, Weronika Banka, Brian Keogan, Jose Maria Urtasun Elizari, Jonathan Dean, Daniel Hare, Cillian F De Gascun
EPI_ISL_18642356	PKC Senen	National Institute of Health Research and Development	Hana Apsari Pawestri, Arie Ardiansyah Nugraha, Fajar Nur Sulistyahadi, Markus Evan Anggia, Subangkit, Herna, IGM Wirabrata
EPI_ISL_18642357	PKC Cakung	National Institute of Health Research and Development	Hana Apsari Pawestri, Arie Ardiansyah Nugraha, Fajar Nur Sulistyahadi, Markus Evan Anggia, Subangkit, Herna, IGM Wirabrata
EPI_ISL_18642358	RS Mitra Keluarga Gading	National Institute of Health Research and Development	Hana Apsari Pawestri, Arie Ardiansyah Nugraha, Fajar Nur Sulistyahadi, Markus Evan Anggia, Subangkit, Herna, IGM Wirabrata
EPI_ISL_18642359	RSUP Persahabatan	National Institute of Health Research and Development	Hana Apsari Pawestri, Arie Ardiansyah Nugraha, Fajar Nur Sulistyahadi, Markus Evan Anggia, Subangkit, Herna, IGM Wirabrata
EPI_ISL_18642360	PKC Cilandak	National Institute of Health Research and Development	Hana Apsari Pawestri, Arie Ardiansyah Nugraha, Fajar Nur Sulistyahadi, Markus Evan Anggia, Subangkit, Herna, IGM Wirabrata
EPI_ISL_18642361	PKC Mampang Prapatan	National Institute of Health Research and Development	Hana Apsari Pawestri, Arie Ardiansyah Nugraha, Fajar Nur Sulistyahadi, Markus Evan Anggia, Subangkit, Herna, IGM Wirabrata
EPI_ISL_18642362	Dinkes Kabupaten Cirebon	National Institute of Health Research and Development	Hana Apsari Pawestri, Arie Ardiansyah Nugraha, Fajar Nur Sulistyahadi, Markus Evan Anggia, Subangkit, Herna, IGM Wirabrata
EPI_ISL_18642363	PKC Setiabudi	National Institute of Health Research and Development	Hana Apsari Pawestri, Arie Ardiansyah Nugraha, Fajar Nur Sulistyahadi, Markus Evan Anggia, Subangkit, Herna, IGM Wirabrata

EPI_ISL_18642364	PKM Pancoran	National Institute of Health Research and Development	Hana Apsari Pawestri, Arie Ardiansyah Nugraha, Fajar Nur Sulistyahadi, Markus Evan Anggia, Subangkit, Herna, IGM Wirabrata
EPI_ISL_18642365	Puskesmas Bambu Apus	National Institute of Health Research and Development	Hana Apsari Pawestri, Arie Ardiansyah Nugraha, Fajar Nur Sulistyahadi, Markus Evan Anggia, Subangkit, Herna, IGM Wirabrata
EPI_ISL_18659828	Erasmus Medical Center Department of Virology	Erasmus Medical Center Department of Virology	Leonard Schuele, Bas Oude Munnink, Marjan Boter, Babette Weller, Babs Verstrepen, Richard Molenkamp, Reina Sikkema, Marion Koopmans
EPI_ISL_18659829, EPI_ISL_18659846	Erasmus Medical Center Department of Virology	Erasmus Medical Center Department of Virology	Leonard Schuele, Marjan Boter, Hayley Cassidy, Babette Weller, Babs Verstrepen, Richard Molenkamp, Marion Koopmans, Bas Oude Munnink
EPI_ISL_18668236, EPI_ISL_18668237, EPI_ISL_18668238, EPI_ISL_18668239, EPI_ISL_18668240, EPI_ISL_18668241, EPI_ISL_18689510	University of Washington, Department of Laboratory Medicine	University of Washington, Department of Laboratory Medicine	Sereewit,J., Nunley,E.B., Xie,H., Roychoudhury,P. and Greninger,A.L.
EPI_ISL_18689511	Bundeswehr Institute of Microbiology	Bundeswehr Institute of Microbiology	Antwerpen,M.H., Lang,D., Sabine,S. and Woelfel,R.
EPI_ISL_18697752	National Institute of Public Health	Institut Pasteur du Cambodge, Virology Unit	Janin Nouhin, Leakhena Pum, Jurre Y Siegers, Chin Savuth, Chau Darapheak, Veasna Duong, Erik A Karlsson
EPI_ISL_18702208, EPI_ISL_18702209, EPI_ISL_18702210, EPI_ISL_18702211, EPI_ISL_18702212, EPI_ISL_18702213, EPI_ISL_18702214, EPI_ISL_18702215, EPI_ISL_18702216, EPI_ISL_18702217, EPI_ISL_18702218, EPI_ISL_18702219, EPI_ISL_18702220, EPI_ISL_18702221, EPI_ISL_18702222, EPI_ISL_18702223, EPI_ISL_18702224, EPI_ISL_18702225, EPI_ISL_18702226, EPI_ISL_18702227, EPI_ISL_18702228, EPI_ISL_18702229, EPI_ISL_18702230, EPI_ISL_18702231, EPI_ISL_18702232, EPI_ISL_18702233, EPI_ISL_18702234, EPI_ISL_18702235, EPI_ISL_18702236, EPI_ISL_18702237, EPI_ISL_18702238, EPI_ISL_18702239, EPI_ISL_18702240, EPI_ISL_18702241, EPI_ISL_18702242, EPI_ISL_18702243, EPI_ISL_18702244, EPI_ISL_18702245, EPI_ISL_18702246, EPI_ISL_18702247, EPI_ISL_18702248, EPI_ISL_18702249, EPI_ISL_18702250, EPI_ISL_18702251, EPI_ISL_18702252, EPI_ISL_18702253, EPI_ISL_18702254, EPI_ISL_18702255, EPI_ISL_18702256, EPI_ISL_18702257			
see above	Center for Vectors and Infectious Diseases Research (CEVDI), National Health Institute Doutor Ricardo Jorge, IP (INSA)	Center for Vectors and Infectious Diseases Research (CEVDI), National Health Institute Doutor Ricardo Jorge, IP (INSA)	Isidro,J., Borges,V., Pinto,M., Sobral,D., Santos,J., Nunes,A., Mixao,V., Ferreira,R., Santos,D., Duarte,S., Vieira,L., Borrego,M., Nuncio,S., Lopes de Carvalho,I., Pelerito,A., Cordeiro,R. and Gomes,J.P.
EPI_ISL_18719998, EPI_ISL_18719999	CT Department of Public Health	CT Department of Public Health	Claire Pearson, Tu N. Nguyen, Kutluhan Incekara, Neranjan V. Perera
EPI_ISL_18739594, EPI_ISL_18739595	Tokyo Metropolitan Institute of Public Health	Tokyo Metropolitan Institute of Public Health	Fumi Kasuya, Wakaba Okada, Ryota Kumagai, Sachiko Harada, Arisa Amano, Michiya Hasegawa, Mami Nagashima, Kenji Sadamasu
EPI_ISL_18744050	National Medical Center	National Medical Center	Jun-sun Park, Hongsoon Yim, Jihye Um, Hyang Su Kim, BumSik Chin, Jaehyun Jeon, Yeonjae Kim, Min-Kyung Kim
EPI_ISL_18746882, EPI_ISL_18746884, EPI_ISL_18746885	Erasmus Medical Center Department of Virology	Erasmus Medical Center Department of Virology	Leonard Schuele, Marjan Boter, Babs Verstrepen, Richard Molenkamp, Marion Koopmans, Bas Oude Munnink
EPI_ISL_18773078, EPI_ISL_18773079, EPI_ISL_18773080, EPI_ISL_18773081, EPI_ISL_18773082, EPI_ISL_18773083, EPI_ISL_18773084, EPI_ISL_18773085, EPI_ISL_18773086, EPI_ISL_18773087, EPI_ISL_18773088, EPI_ISL_18773089, EPI_ISL_18773090, EPI_ISL_18773091, EPI_ISL_18773092, EPI_ISL_18773093, EPI_ISL_18773094, EPI_ISL_18773095, EPI_ISL_18773096, EPI_ISL_18773097, EPI_ISL_18773098, EPI_ISL_18773099, EPI_ISL_18773100, EPI_ISL_18773101, EPI_ISL_18773102, EPI_ISL_18773103, EPI_ISL_18773104, EPI_ISL_18773105, EPI_ISL_18773106, EPI_ISL_18773107, EPI_ISL_18773108			
see above	Centre for Biological Threats - Highly Pathogenic Viruses, Robert Koch Institute	Centre for Biological Threats - Highly Pathogenic Viruses, Robert Koch Institute	Brinkmann,A., Kohl,C., Schrick,L., Michel,J., Schaade,L. and Nitsche,A.
EPI_ISL_18773109, EPI_ISL_18773110, EPI_ISL_18773111, EPI_ISL_18773112, EPI_ISL_18773113, EPI_ISL_18773114, EPI_ISL_18773115, EPI_ISL_18773116, EPI_ISL_18773117, EPI_ISL_18773118, EPI_ISL_18773119, EPI_ISL_18773120, EPI_ISL_18773121, EPI_ISL_18773122, EPI_ISL_18773123, EPI_ISL_18773124, EPI_ISL_18773125, EPI_ISL_18773126, EPI_ISL_18773127, EPI_ISL_18773128, EPI_ISL_18773129, EPI_ISL_18773130, EPI_ISL_18773131, EPI_ISL_18773132, EPI_ISL_18773133, EPI_ISL_18773134, EPI_ISL_18773135, EPI_ISL_18773136, EPI_ISL_18773137, EPI_ISL_18773138, EPI_ISL_18773139, EPI_ISL_18773140, EPI_ISL_18773141, EPI_ISL_18773142, EPI_ISL_18773143, EPI_ISL_18773144, EPI_ISL_18773145, EPI_ISL_18773146, EPI_ISL_18773147, EPI_ISL_18773148, EPI_ISL_18773149, EPI_ISL_18773150			
see above	Animal Health - Istituto Zooprofilattico Sperimentale del Mezzogiorno	Animal Health - Istituto Zooprofilattico Sperimentale del Mezzogiorno	Viscardi,M., Cozzolino,L., Rinaldi,A., De Martinis,C., Cardillo,L., Tiberio,C., Falco,R., Guarino,V., D'Auria,G., Nappo,F., Atripaldi,L., Coppola,M.G. and Fusco,G.
EPI_ISL_18781624, EPI_ISL_18781625, EPI_ISL_18781626, EPI_ISL_18781627, EPI_ISL_18781628, EPI_ISL_18781629, EPI_ISL_18781630, EPI_ISL_18781631, EPI_ISL_18781632, EPI_ISL_18781633, EPI_ISL_18781634, EPI_ISL_18781635, EPI_ISL_18781636, EPI_ISL_18781637, EPI_ISL_18781785, EPI_ISL_18781786, EPI_ISL_18781787, EPI_ISL_18781788, EPI_ISL_18781790, EPI_ISL_18781791, EPI_ISL_18781792, EPI_ISL_18781793, EPI_ISL_18781794, EPI_ISL_18781795, EPI_ISL_18781796, EPI_ISL_18781797, EPI_ISL_18781798, EPI_ISL_18781799, EPI_ISL_18781806, EPI_ISL_18781807, EPI_ISL_18781808, EPI_ISL_18781809, EPI_ISL_18781810, EPI_ISL_18781811, EPI_ISL_18781812, EPI_ISL_18781813, EPI_ISL_18781815, EPI_ISL_18781816, EPI_ISL_18781817, EPI_ISL_18781818, EPI_ISL_18781819, EPI_ISL_18781820, EPI_ISL_18781833, EPI_ISL_18781834, EPI_ISL_18781835, EPI_ISL_18781836, EPI_ISL_18781837, EPI_ISL_18781839, EPI_ISL_18781840, EPI_ISL_18781841, EPI_ISL_18781842, EPI_ISL_18781843, EPI_ISL_18781844, EPI_ISL_18781845, EPI_ISL_18781846, EPI_ISL_18781847, EPI_ISL_18781848, EPI_ISL_18781849, EPI_ISL_18781912, EPI_ISL_18781913, EPI_ISL_18781915, EPI_ISL_18781916, EPI_ISL_18781917, EPI_ISL_18781918, EPI_ISL_18781919, EPI_ISL_18781920, EPI_ISL_18781921, EPI_ISL_18781922, EPI_ISL_18781923, EPI_ISL_18781924, EPI_ISL_18781925, EPI_ISL_18781926, EPI_ISL_18781928, EPI_ISL_18781929, EPI_ISL_18786340, EPI_ISL_18786341, EPI_ISL_18786342, EPI_ISL_18786343, EPI_ISL_18786346, EPI_ISL_18786347, EPI_ISL_18786348, EPI_ISL_18786349, EPI_ISL_18786350, EPI_ISL_18786351, EPI_ISL_18786352, EPI_ISL_18786354, EPI_ISL_18786356, EPI_ISL_18786357, EPI_ISL_18786358, EPI_ISL_18786359			
see above	California Department of Public Health	California Department of Public Health	Kath, C., Haw, M., Espinosa, A., and Hacker, J.
EPI_ISL_18798834	PKM Mampang Prapatan	National Institute of Health Research and Development	Hana Apsari Pawestri, Arie Ardiansyah Nugraha, Fajar Nur Sulistyahadi, Markus Evan Anggia, Subangkit
EPI_ISL_18798835	PKC Kebayoran Lama	National Institute of Health Research and Development	Hana Apsari Pawestri, Arie Ardiansyah Nugraha, Fajar Nur Sulistyahadi, Markus Evan Anggia, Subangkit
EPI_ISL_18798836	RS Grha Kedoya Jakarta	National Institute of Health Research and Development	Hana Apsari Pawestri, Arie Ardiansyah Nugraha, Fajar Nur Sulistyahadi, Markus Evan Anggia, Subangkit
EPI_ISL_18798837	PKC Kebayoran Baru	National Institute of Health Research and Development	Hana Apsari Pawestri, Arie Ardiansyah Nugraha, Fajar Nur Sulistyahadi, Markus Evan Anggia, Subangkit
EPI_ISL_18798838	PKC Tanah Abang	National Institute of Health Research and Development	Hana Apsari Pawestri, Arie Ardiansyah Nugraha, Fajar Nur Sulistyahadi, Markus Evan Anggia, Subangkit
EPI_ISL_18798839	PKM Bogor Timur	National Institute of Health Research and Development	Hana Apsari Pawestri, Arie Ardiansyah Nugraha, Fajar Nur Sulistyahadi, Markus Evan Anggia, Subangkit
EPI_ISL_18798840	PKM Warung Jambu	National Institute of Health Research and Development	Hana Apsari Pawestri, Arie Ardiansyah Nugraha, Fajar Nur Sulistyahadi, Markus Evan Anggia, Subangkit
EPI_ISL_18798841	PKC Pademangan	National Institute of Health Research and Development	Hana Apsari Pawestri, Arie Ardiansyah Nugraha, Fajar Nur Sulistyahadi, Markus Evan Anggia, Subangkit
EPI_ISL_18798842	PKC Kebayoran Baru	National Institute of Health Research and Development	Hana Apsari Pawestri, Arie Ardiansyah Nugraha, Fajar Nur Sulistyahadi, Markus Evan Anggia, Subangkit
EPI_ISL_18822108, EPI_ISL_18822109	Quest Diagnostics Nichols Institute	Los Angeles County Public Health Laboratories	S. McCann et al
EPI_ISL_18846272, EPI_ISL_18846273, EPI_ISL_18846274, EPI_ISL_18846275, EPI_ISL_18846276, EPI_ISL_18846277, EPI_ISL_18846278	Centre for Biological Threats, Highly Pathogenic Viruses, Robert Koch Institute	Centre for Biological Threats, Highly Pathogenic Viruses, Robert Koch Institute	Brinkmann,A., Kohl,C., Schrick,L., Michel,J., Schaade,L. and Nitsche,A.
EPI_ISL_18846279, EPI_ISL_18846280, EPI_ISL_18846281, EPI_ISL_18846282, EPI_ISL_18846283, EPI_ISL_18846284, EPI_ISL_18846285, EPI_ISL_18846286, EPI_ISL_18846287, EPI_ISL_18846288, EPI_ISL_18846289, EPI_ISL_18846290, EPI_ISL_18846291, EPI_ISL_18846292, EPI_ISL_18846293, EPI_ISL_18846294, EPI_ISL_18846295, EPI_ISL_18846296, EPI_ISL_18846297, EPI_ISL_18846298, EPI_ISL_18846299, EPI_ISL_18846300, EPI_ISL_18846301, EPI_ISL_18846302, EPI_ISL_18846303, EPI_ISL_18846304, EPI_ISL_18846305, EPI_ISL_18846306, EPI_ISL_18846307, EPI_ISL_18846308, EPI_ISL_18846309, EPI_ISL_18846310, EPI_ISL_18846311, EPI_ISL_18846312, EPI_ISL_18846313, EPI_ISL_18846314, EPI_ISL_18846315, EPI_ISL_18846316, EPI_ISL_18846317, EPI_ISL_18846318, EPI_ISL_18846319, EPI_ISL_18846320			
see above	Animal Health, Istituto Zooprofilattico Sperimentale del Mezzogiorno	Hanimal Health, Istituto Zooprofilattico Sperimentale del Mezzogiorno	Viscardi,M., Cozzolino,L., Rinaldi,A., De Martinis,C., Cardillo,L., Tiberio,C., Falco,R., Guarino,V., D'Auria,G., Nappo,F., Atripaldi,L., Coppola,M.G. and Fusco,G.
EPI_ISL_18846321, EPI_ISL_18846322, EPI_ISL_18846323	University of Washington, Department of Laboratory Medicine	University of Washington, Department of Laboratory Medicine	Sereewit,J., Nunley,E.B., Xie,H., Roychoudhury,P. and Greninger,A.L.
EPI_ISL_18890763, EPI_ISL_18890764, EPI_ISL_18890765, EPI_ISL_18890766, EPI_ISL_18890767, EPI_ISL_18890768, EPI_ISL_18890769, EPI_ISL_18890770, EPI_ISL_18890771, EPI_ISL_18890772, EPI_ISL_18890773, EPI_ISL_18890774, EPI_ISL_18890775, EPI_ISL_18890776, EPI_ISL_18890777, EPI_ISL_18890778, EPI_ISL_18890779			
see above	Animal Health, Istituto Zooprofilattico Sperimentale del Mezzogiorno	Animal Health, Istituto Zooprofilattico Sperimentale del Mezzogiorno	Viscardi,M., Cozzolino,L., Rinaldi,A., De Martinis,C., Cardillo,L., Tiberio,C., Falco,R., Guarino,V., D'Auria,G., Nappo,F., Atripaldi,L., Coppola,M.G. and Fusco,G.
EPI_ISL_18959309, EPI_ISL_18959310, EPI_ISL_18959311, EPI_ISL_18959312, EPI_ISL_18959313, EPI_ISL_18959314, EPI_ISL_18959315, EPI_ISL_18959316, EPI_ISL_18959317, EPI_ISL_18959318, EPI_ISL_18959319, EPI_ISL_18959320, EPI_ISL_18959321, EPI_ISL_18959322, EPI_ISL_18959323			
see above	California Department of Public Health	California Department of Public Health	Kath, C., Haw, M., Espinosa, A., and Hacker, J.
EPI_ISL_18971016, EPI_ISL_18971017, EPI_ISL_18971018	Central Public Health Laboratory, State Health Surveillance Center of the Rio Grande do Sul State Health Department (LACEN/CEVS/SES-RS)	Center for Scientific and Technological Development, State Center for Health Surveillance of the Secretary of Health of the State of Rio Grande do Sul (CDCT/CEVS/SES-RS)	Fernanda Godinho, Richard Steiner Salvato
EPI_ISL_18993169, EPI_ISL_18993170, EPI_ISL_18993171, EPI_ISL_18993172, EPI_ISL_18993173	HCMC Hospital of Dermato Venereology	STIs Lab, Pasteur Institute in Ho Chi Minh City	Yen Nhi Nguyen, Tam-Duong Le-Ha, Lien Le, Hanh Lan Nguyen Thi, Thang Minh Cao, Thinh Viet Nguyen, Quang Duy Pham, Quang Luong Chan, Thuong Vu Nguyen, Trung Vu Nguyen
EPI_ISL_18993174	Lam Dong 2 Hospital	STIs Lab, Pasteur Institute in Ho Chi Minh City	Yen Nhi Nguyen, Tam-Duong Le-Ha, Lien Le, Hanh Lan Nguyen Thi, Thang Minh Cao, Thinh Viet Nguyen, Quang Duy Pham, Quang Luong Chan, Thuong Vu Nguyen, Trung Vu Nguyen
EPI_ISL_18993175	Can Tho Hospital of Dermato Venereology	STIs Lab, Pasteur Institute in Ho Chi Minh City	Yen Nhi Nguyen, Tam-Duong Le-Ha, Lien Le, Hanh Lan Nguyen Thi, Thang Minh Cao, Thinh Viet Nguyen, Quang Duy Pham, Quang Luong Chan, Thuong Vu Nguyen, Trung Vu Nguyen
EPI_ISL_18993177, EPI_ISL_18993178	HCMC Hospital of Dermato Venereology	STIs Lab, Pasteur Institute in Ho Chi Minh City	Yen Nhi Nguyen, Tam-Duong Le-Ha, Lien Le, Hanh Lan Nguyen Thi, Thang Minh Cao, Thinh Viet Nguyen, Quang Duy Pham, Quang Luong Chan, Thuong Vu Nguyen, Trung Vu Nguyen
EPI_ISL_18993179	CDC Soc Trang	STIs Lab, Pasteur Institute in Ho Chi Minh City	Yen Nhi Nguyen, Tam-Duong Le-Ha, Lien Le, Hanh Lan Nguyen Thi, Thang Minh Cao, Thinh Viet Nguyen, Quang Duy Pham, Quang Luong Chan, Thuong Vu Nguyen, Trung Vu Nguyen
EPI_ISL_18993180	HCMC Hospital of Dermato Venereology	STIs Lab, Pasteur Institute in Ho Chi Minh City	Yen Nhi Nguyen, Tam-Duong Le-Ha, Lien Le, Hanh Lan Nguyen Thi, Thang Minh Cao, Thinh Viet Nguyen, Quang Duy Pham, Quang Luong Chan, Thuong Vu Nguyen, Trung Vu Nguyen
EPI_ISL_18993181	Tan Thanh Health Center	STIs Lab, Pasteur Institute in Ho Chi Minh City	Yen Nhi Nguyen, Tam-Duong Le-Ha, Lien Le, Hanh Lan Nguyen Thi, Thang Minh Cao, Thinh Viet Nguyen, Quang Duy Pham, Quang Luong Chan, Thuong Vu Nguyen, Trung Vu Nguyen
EPI_ISL_18993183	Quest Diagnostics Nichols Institute	Los Angeles County Public Health Laboratories	J. Garrigues et. al.
EPI_ISL_18993184, EPI_ISL_18993185	Ucla Healthcare Clinical Laboratory - Brentwood	Los Angeles County Public Health Laboratories	J. Garrigues et. al.
EPI_ISL_18993186, EPI_ISL_18993187, EPI_ISL_18993188, EPI_ISL_18993189, EPI_ISL_18993190, EPI_ISL_18993191, EPI_ISL_18993193	Quest Diagnostics Nichols Institute	Los Angeles County Public Health Laboratories	J. Garrigues et. al.
EPI_ISL_18993194, EPI_ISL_18993195, EPI_ISL_18993196	Laboratory Corporation Of America	Los Angeles County Public Health Laboratories	J. Garrigues et. al.
EPI_ISL_18993197, EPI_ISL_18993198	Los Angeles County Public Health Laboratory	Los Angeles County Public Health Laboratories	J. Garrigues et. al.
EPI_ISL_18993199, EPI_ISL_18993200	Quest Diagnostics Nichols Institute	Los Angeles County Public Health Laboratories	J. Garrigues et. al.
EPI_ISL_18993201	Los Angeles County Public Health Laboratory	Los Angeles County Public Health Laboratories	J. Garrigues et. al.
EPI_ISL_18993202, EPI_ISL_18993203	Quest Diagnostics Nichols Institute	Los Angeles County Public Health Laboratories	J. Garrigues et. al.
EPI_ISL_18993204, EPI_ISL_18993207	Laboratory Corporation Of America	Los Angeles County Public Health Laboratories	J. Garrigues et. al.
EPI_ISL_18993208, EPI_ISL_18993209	Quest Diagnostics Nichols Institute	Los Angeles County Public Health Laboratories	J. Garrigues et. al.
EPI_ISL_18993210	Arup Laboratories	Los Angeles County Public Health Laboratories	J. Garrigues et. al.
EPI_ISL_18993211, EPI_ISL_18993212, EPI_ISL_18993213	Laboratory Corporation Of America	Los Angeles County Public Health Laboratories	J. Garrigues et. al.

EPI_ISL_18993214	Quest Diagnostics Nichols Institute	Los Angeles County Public Health Laboratories	J. Garrigues et. al.
EPI_ISL_18993215	Laboratory Corporation Of America	Los Angeles County Public Health Laboratories	J. Garrigues et. al.
EPI_ISL_18993954, EPI_ISL_18993955, EPI_ISL_18993961, EPI_ISL_18993963, EPI_ISL_18993964, EPI_ISL_18993967, EPI_ISL_18993969	Center for Vectors and Infectious Diseases Research (CEVDI), National Health Institute Doutor Ricardo Jorge, IP (INSA)	Center for Vectors and Infectious Diseases Research (CEVDI), National Health Institute Doutor Ricardo Jorge, IP (INSA)	Isidro,J., Borges,V., Pinto,M., Sobral,D., Santos,J., Nunes,A., Mixao,V., Ferreira,R., Santos,D., Duarte,S., Vieira,L., Borrego,M.J., Nuncio,S., Lopes de Carvalho,I., Pelerito,A., Cordeiro,R. and Gomes,J.P.
EPI_ISL_19004044	Centre de Recherche en Sciences Naturelles de Lwiro	Centre de Recherche en Sciences Naturelles de Lwiro	Leandre Murhula Masirika, Jean Claude Udahemuka, Pacifique Ndishimye, Gustavo Sganzerla Martinez, Patricia Kelvin, Maliyamungu Bubala Nadine, Bilembo Kitwanda Steeven, Franklin Kumbana Mweshi, Léandre Mutimbwa Mambo, Bas B. Oude Munnink, Justin Bengehya Mbiribindi, Freddy Belesi Siangoli, Trudie Lang, Jean M. Malekani, Frank M. Aarestrup, Marion Koopmans, Leonard Schuele, Jean Pierre Musabyimana, Brigitte Umutoni, Ali Toloue, Benjamin Hewins, Mansi Dutt, Anuj Kumar, Alyson A. Kelvin, Jean-Paul Kabemba Lukusa, Christian Gortazar, David J Kelvin, Luis Flores
EPI_ISL_19004045, EPI_ISL_19004046	Centre de Recherche en Sciences Naturelles de Lwiro	Centre de Recherche en Sciences Naturelles de Lwiro	Leandre Murhula Masirika , Jean Claude Udahemuka, Pacifique Ndishimye, Gustavo Sganzerla Martinez, Patricia Kelvin, Maliyamungu Bubala Nadine, Bilembo Kitwanda Steeven, Franklin Kumbana Mweshi, Léandre Mutimbwa Mambo, Bas B. Oude Munnink, Justin Bengehya Mbiribindi, Freddy Belesi Siangoli, Trudie Lang, Jean M. Malekani, Frank M. Aarestrup, Marion Koopmans, Leonard Schuele, Jean Pierre Musabyimana, Brigitte Umutoni, Ali Toloue, Benjamin Hewins, Mansi Dutt, Anuj Kumar, Alyson A. Kelvin, Jean-Paul Kabemba Lukusa, Christian Gortazar, David J Kelvin, Luis Flores
EPI_ISL_19012435	Laboratorio de Enterovirus, Instituto Oswaldo Cruz, Fiocruz	Oswaldo Cruz Foundation Laboratory of Respiratory Virus and Measles	Paola Resende, Elisa Calvalcante Pereira, Bruna Mendonça da Silva, Jéssica Graça Macedo de Carvalho, Larissa Macedo Pinto, Victor Guimaraes, Marilda Siqueira, Renan da Silva Faustino, Marilia Santini, Edson Elias da Silva on behalf of the Fiocruz Genomic Surveillance Network
EPI_ISL_19016746, EPI_ISL_19016747, EPI_ISL_19016748, EPI_ISL_19016749, EPI_ISL_19016763, EPI_ISL_19016764, EPI_ISL_19016765, EPI_ISL_19016766, EPI_ISL_19016767, EPI_ISL_19016768, EPI_ISL_19016769, EPI_ISL_19016771, EPI_ISL_19016772, EPI_ISL_19016773			
see above	Quest Diagnostics	RIPHL at Rush University Medical Center	Stefan Green, Kevin Kunstman, Hannah Barbian, Sofiya Bobrovska, Felix Araujo Perez, Erin Newcomer
EPI_ISL_19022858, EPI_ISL_19022859, EPI_ISL_19022860, EPI_ISL_19022861, EPI_ISL_19022862	Osaka Metropolitan University, Graduate School of Medicine, Department of Virology and Parasitology	Osaka Metropolitan University, Graduate School of Medicine, Department of Virology and Parasitology	Evariste Tshibangu-Kabamba, Natsuko Kaku, Eisuke Adachi, Mayo Yasugi, Takuya Yamamoto, Takuto Nogimori, Yoshiyuki Wakabayashi, Yasutoshi Kido
EPI_ISL_19027495, EPI_ISL_19027496, EPI_ISL_19027500, EPI_ISL_19027501, EPI_ISL_19027502, EPI_ISL_19027504, EPI_ISL_19027507, EPI_ISL_19027511, EPI_ISL_19027512, EPI_ISL_19027514, EPI_ISL_19027515, EPI_ISL_19027518, EPI_ISL_19027519, EPI_ISL_19027523, EPI_ISL_19027525, EPI_ISL_19027527, EPI_ISL_19027529, EPI_ISL_19027530, EPI_ISL_19027531, EPI_ISL_19027532, EPI_ISL_19027534, EPI_ISL_19027535, EPI_ISL_19027536, EPI_ISL_19027537, EPI_ISL_19027541, EPI_ISL_19027543, EPI_ISL_19027546, EPI_ISL_19027548, EPI_ISL_19027551, EPI_ISL_19027552, EPI_ISL_19027554, EPI_ISL_19027555, EPI_ISL_19027558, EPI_ISL_19027560, EPI_ISL_19027561, EPI_ISL_19027562, EPI_ISL_19027563, EPI_ISL_19027568, EPI_ISL_19027571, EPI_ISL_19027573, EPI_ISL_19027574, EPI_ISL_19027575, EPI_ISL_19027576			
see above	NYC Department of Health and Mental Hygiene, Public Health Laboratory	NYC Department of Health and Mental Hygiene, Public Health Laboratory	Clabby,T.T., Wang,J.C., Amin,H.S., Taki,F., Su,M., De La Cruz,N., Olsen,A., Thi,C., Silver,S., Akther,S., Chowdhury,M., Omoregie,E. and Siemetzki-Kapoor,U.
EPI_ISL_19028687, EPI_ISL_19028692, EPI_ISL_19028714, EPI_ISL_19028750, EPI_ISL_19028758, EPI_ISL_19028766, EPI_ISL_19028768, EPI_ISL_19028774, EPI_ISL_19028781, EPI_ISL_19028782, EPI_ISL_19028789, EPI_ISL_19028798, EPI_ISL_19028800, EPI_ISL_19028803, EPI_ISL_19028811, EPI_ISL_19028813			
see above	British Columbia Centre For Disease Control	BCCDC Public Health Laboratory	Prystajecky,Natalie; Tyson,John; Jassem,Agatha; Lee,Tracy; Azana,Rob; Fung,Janet; Chan,Michael; Cheung,Branco; Tsang,Frankie; Newman,Tara; Yang,Kevin; Russell,Shannon; Zlosnik,James; Hoang,Linda
EPI_ISL_19058864, EPI_ISL_19058865, EPI_ISL_19058866, EPI_ISL_19058867, EPI_ISL_19058868, EPI_ISL_19058869, EPI_ISL_19058870, EPI_ISL_19058871, EPI_ISL_19058872, EPI_ISL_19058873	Guangdong Provincial Center for Disease Control and Prevention, Institute of Pathogenic Microbiology	Guangdong Provincial Center for Disease Control and Prevention, Institute of Pathogenic Microbiology	Li,B., Zhao,W. and Shen,C.
EPI_ISL_19079342	Centre de Recherche en Sciences Naturelles de Lwiro (CRSN Lwiro)	Centre de Recherche en Sciences Naturelles de Lwiro (CRSN Lwiro)	Leandre M Masirika, Anuj Kumar, Mansi Dutt, Ali Toloue Ostadgavahi, Benjamin Hewins, Maliyamungu B Nadine, Bilembo K Steeven, Franklin K Mweshi, Léandre M Mambo, Justin B Mbiribindi, Freddy B Siangoli, Alyson A Kelvin, Jean Claude Udahemuka, Patricia Kelvin, Luis Flores, David J Kelvin, Gustavo Sganzerla Martinez
EPI_ISL_19093802, EPI_ISL_19093805, EPI_ISL_19093807, EPI_ISL_19093809, EPI_ISL_19093810, EPI_ISL_19093811, EPI_ISL_19093813, EPI_ISL_19093818, EPI_ISL_19093819, EPI_ISL_19093820, EPI_ISL_19093821, EPI_ISL_19093822, EPI_ISL_19093824, EPI_ISL_19093825, EPI_ISL_19093826, EPI_ISL_19093827, EPI_ISL_19093830, EPI_ISL_19093831, EPI_ISL_19093832, EPI_ISL_19093833, EPI_ISL_19093834			
see above	Pathogen Genomic Laboratory, Institut National de Recherche Biomedicale	Pathogen Genomic Laboratory, Institut National de Recherche Biomedicale	Vakaniaki,E.H., Kaciat,C., Kinganda - Lusamaki,E., O'Toole,A., Wawina -Bokalanga,T., Mukadi - Bamuleka,D., Amuri,A.A., Parker,E., Muswamba-Kayembe,P.-C., Makangara - Cigolo,J.-C., Mulopo - Mukanya,N., Pukuta - Simbu,E., Mujula,Y., Nundu,S.S., Akil - Bandali,P., Kavunga,H., Lushima,R.S., Vercauteren,K., Sam-Agudu,N.A., Mills,E.J., Tshiani - Mbaya,O., Hoff,N., Rimoin,A.W., Hensley,L.E., Kundrachuk,J., Ayouba,A., Peeters,M., Delaporte,E., Nachega,J.B., Ahuka - Mundeke,S., Muyembe - Tamfum,J.-J., Rambaut,A., Liesenborghs,L. and Mbala - Kingebeni,P.
EPI_ISL_19108154, EPI_ISL_19108155, EPI_ISL_19108156, EPI_ISL_19108157, EPI_ISL_19108158, EPI_ISL_19108159, EPI_ISL_19108160, EPI_ISL_19108161, EPI_ISL_19108162, EPI_ISL_19108163, EPI_ISL_19108164, EPI_ISL_19108165, EPI_ISL_19108166, EPI_ISL_19108167, EPI_ISL_19108168, EPI_ISL_19108169, EPI_ISL_19108170, EPI_ISL_19108171			
see above	Oxford University Clinical Research Unt	Oxford University Clinical Research Unt	Huynh Thi Thuy Hoa, Nguyen Thanh Dung, Le Manh Hung, Nguyen Thi Thu Hong, Vo Truong Quy, Hoang Truong, Nguyen Trong Duy, Tran Minh Hoang, Nguyen Thi Thanh, Mai Hong Phuoc, Nguyen Nhut Thong, Nguyen Duc Huy, Vu Thi Kim Thoa, Nghiem My Ngoc, Vo Trong Vuong, Ngo Tan Tai, Huynh Kim Nhung, Dao Phuong Linh, Pham Thi Ngoc Thoa, Lam Minh Yen, Nguyen Thi Thao, Tran Ba Thien, Truong Hoang Chau Truc, Le Kim Thanh, Vo Tan Hoang, Nguyen Thanh Ngoc, Tran Tan Thanh, Louise Thwaites, Nguyen Van Vinh Chau, Guy Thwaites, Nguyen To Anh, Le Van Tan
EPI_ISL_19131344	Indian Council of Medical Research-National Institute of Virology, Microbial Containment Complex	Indian Council of Medical Research-National Institute of Virology, Microbial Containment Complex	Pragya D. Yadav
EPI_ISL_19143445, EPI_ISL_19143446, EPI_ISL_19143447, EPI_ISL_19143448, EPI_ISL_19143449, EPI_ISL_19143450, EPI_ISL_19143451, EPI_ISL_19143452, EPI_ISL_19143453, EPI_ISL_19143454, EPI_ISL_19143458, EPI_ISL_19143459, EPI_ISL_19143460, EPI_ISL_19143461, EPI_ISL_19143476			
see above	California Department of Public Health	California Department of Public Health	Kath, C., Haw, M., Espinosa, A., and Hacker, J.
EPI_ISL_19158911, EPI_ISL_19158912, EPI_ISL_19158913	HCMC Hospital of Dermato Venereology	STIs Lab, Pasteur Institute in Ho Chi Minh city	Yen Nhi Nguyen, Tam-Duong Le-Ha, Lien Le, Hanh Lan Nguyen Thi, Thang Minh Cao, Thinh Viet Nguyen, Quang Duy Pham, Quang Luong Chan, Thuong Vu Nguyen, Trung Vu Nguyen
EPI_ISL_19158914	Thu Duc Medical Center	STIs Lab, Pasteur Institute in Ho Chi Minh city	Yen Nhi Nguyen, Tam-Duong Le-Ha, Lien Le, Hanh Lan Nguyen Thi, Thang Minh Cao, Thinh Viet Nguyen, Quang Duy Pham, Quang Luong Chan, Thuong Vu Nguyen, Trung Vu Nguyen
EPI_ISL_19158915, EPI_ISL_19158916, EPI_ISL_19158917, EPI_ISL_19158918	HCMC Hospital of Dermato Venereology	STIs Lab, Pasteur Institute in Ho Chi Minh city	Yen Nhi Nguyen, Tam-Duong Le-Ha, Lien Le, Hanh Lan Nguyen Thi, Thang Minh Cao, Thinh Viet Nguyen, Quang Duy Pham, Quang Luong Chan, Thuong Vu Nguyen, Trung Vu Nguyen
EPI_ISL_19158927	Military Hospital 175	STIs Lab, Pasteur Institute in Ho Chi Minh city	Yen Nhi Nguyen, Tam-Duong Le-Ha, Lien Le, Hanh Lan Nguyen Thi, Thang Minh Cao, Thinh Viet Nguyen, Quang Duy Pham, Quang Luong Chan, Thuong Vu Nguyen, Trung Vu Nguyen
EPI_ISL_19158928, EPI_ISL_19158929, EPI_ISL_19158930, EPI_ISL_19158931, EPI_ISL_19158932, EPI_ISL_19158933, EPI_ISL_19158934, EPI_ISL_19158935, EPI_ISL_19158942, EPI_ISL_19158943, EPI_ISL_19158944			
see above	HCMC Hospital of Dermato Venereology	STIs Lab, Pasteur Institute in Ho Chi Minh city	Yen Nhi Nguyen, Tam-Duong Le-Ha, Lien Le, Hanh Lan Nguyen Thi, Thang Minh Cao, Thinh Viet Nguyen, Quang Duy Pham, Quang Luong Chan, Thuong Vu Nguyen, Trung Vu Nguyen
EPI_ISL_19159108	RSPI Sulianti Saroso	Balai Besar Laboratorium Biologi Kesehatan	Hana Apsari Pawestri, Arie Ardiansyah Nugraha, Fajar Nur Sulistyahadi, Markus Evan Anggia, Subangkit
EPI_ISL_19159109	RSUP Dr Hasan Sadikin	Balai Besar Laboratorium Biologi Kesehatan	Hana Apsari Pawestri, Arie Ardiansyah Nugraha, Fajar Nur Sulistyahadi, Markus Evan Anggia, Subangkit
EPI_ISL_19159110	Puskesmas Cilodong	Balai Besar Laboratorium Biologi Kesehatan	Hana Apsari Pawestri, Arie Ardiansyah Nugraha, Fajar Nur Sulistyahadi, Markus Evan Anggia, Subangkit
EPI_ISL_19159111	RSUD Cengkareng	Balai Besar Laboratorium Biologi Kesehatan	Hana Apsari Pawestri, Arie Ardiansyah Nugraha, Fajar Nur Sulistyahadi, Markus Evan Anggia, Subangkit
EPI_ISL_19159112	Eka Hospital BSD	Balai Besar Laboratorium Biologi Kesehatan	Hana Apsari Pawestri, Arie Ardiansyah Nugraha, Fajar Nur Sulistyahadi, Markus Evan Anggia, Subangkit
EPI_ISL_19159113	PKC Tebet	Balai Besar Laboratorium Biologi Kesehatan	Hana Apsari Pawestri, Arie Ardiansyah Nugraha, Fajar Nur Sulistyahadi, Markus Evan Anggia, Subangkit
EPI_ISL_19159114	PKC Menteng	Balai Besar Laboratorium Biologi Kesehatan	Hana Apsari Pawestri, Arie Ardiansyah Nugraha, Fajar Nur Sulistyahadi, Markus Evan Anggia, Subangkit
EPI_ISL_19170427, EPI_ISL_19170428, EPI_ISL_19170429, EPI_ISL_19170430, EPI_ISL_19170431, EPI_ISL_19170432, EPI_ISL_19170433, EPI_ISL_19170434, EPI_ISL_19170435, EPI_ISL_19170436, EPI_ISL_19170437, EPI_ISL_19170438, EPI_ISL_19170439, EPI_ISL_19170440, EPI_ISL_19170441, EPI_ISL_19170442, EPI_ISL_19170447, EPI_ISL_19170448, EPI_ISL_19170449, EPI_ISL_19170450, EPI_ISL_19170451, EPI_ISL_19170452, EPI_ISL_19170459, EPI_ISL_19170460, EPI_ISL_19170461, EPI_ISL_19170462, EPI_ISL_19170463, EPI_ISL_19170464, EPI_ISL_19170465, EPI_ISL_19170466, EPI_ISL_19170467, EPI_ISL_19170468, EPI_ISL_19170469, EPI_ISL_19170470, EPI_ISL_19170471, EPI_ISL_19170472, EPI_ISL_19170473, EPI_ISL_19170474, EPI_ISL_19170475, EPI_ISL_19170476, EPI_ISL_19170477, EPI_ISL_19170478, EPI_ISL_19170479, EPI_ISL_19170480, EPI_ISL_19170481, EPI_ISL_19170482, EPI_ISL_19170483, EPI_ISL_19170484, EPI_ISL_19170485, EPI_ISL_19170486, EPI_ISL_19170487			
see above	California Department of Public Health	California Department of Public Health	Kath, C., Haw, M., Espinosa, A., and Hacker, J.
EPI_ISL_19193028	California Department of Public Health, Viral and Rickettsial Disease Laboratory	California Department of Public Health, Viral and Rickettsial Disease Laboratory	Kath,C., Haw,M., Espinosa,A. and Hacker,J.
EPI_ISL_19205402, EPI_ISL_19205403, EPI_ISL_19205404, EPI_ISL_19205405, EPI_ISL_19205406, EPI_ISL_19205407	Laboratorio de Enterovirus, Instituto Oswaldo Cruz, Fiocruz	Instituto Oswaldo Cruz FIOCRUZ - Laboratory of Respiratory Viruses and Measles (LVRS)	Paola Resende, Elisa Calvalcante Pereira, Bruna Mendonça da Silva, Jéssica Graça Macedo de Carvalho, Larissa Macedo Pinto, Victor Guimaraes, Luciana Appolinario, Alice Sampaio, Marilda Siqueira, Renan da Silva Faustino, Marilia Santini, Edson Elias da Silva on behalf of the Fiocruz Genomic Surveillance Network
EPI_ISL_19230662, EPI_ISL_19230663, EPI_ISL_19230664, EPI_ISL_19230665, EPI_ISL_19230666	Charite - Universitatsmedizin Berlin	Charite - Universitatsmedizin Berlin	Obermeier,P.E., Plinke,C.F., Brinkmann,A., Lachmann,R., Melchert,J., Corman,V.M., Nitsche,A., Marcus,U., Schmidt,A.J., Jansen,K. and Buder,S.C.
EPI_ISL_19255461, EPI_ISL_19255463, EPI_ISL_19255464, EPI_ISL_19255465, EPI_ISL_19255466, EPI_ISL_19255469, EPI_ISL_19255470, EPI_ISL_19255471, EPI_ISL_19255472, EPI_ISL_19255473, EPI_ISL_19255474, EPI_ISL_19255475, EPI_ISL_19255476, EPI_ISL_19255477, EPI_ISL_19255478, EPI_ISL_19255479, EPI_ISL_19255480, EPI_ISL_19255481, EPI_ISL_19255482, EPI_ISL_19255483, EPI_ISL_19255484, EPI_ISL_19255485, EPI_ISL_19255486, EPI_ISL_19255487, EPI_ISL_19255488, EPI_ISL_19255489, EPI_ISL_19255490, EPI_ISL_19255491, EPI_ISL_19255492, EPI_ISL_19255494, EPI_ISL_19255495, EPI_ISL_19255496, EPI_ISL_19255497, EPI_ISL_19255498, EPI_ISL_19255499, EPI_ISL_19255500, EPI_ISL_19255501, EPI_ISL_19255502, EPI_ISL_19255503, EPI_ISL_19255504, EPI_ISL_19255505, EPI_ISL_19255506, EPI_ISL_19255507, EPI_ISL_19255508, EPI_ISL_19255509, EPI_ISL_19255510, EPI_ISL_19255511, EPI_ISL_19255512, EPI_ISL_19255513, EPI_ISL_19255514, EPI_ISL_19255515, EPI_ISL_19255516, EPI_ISL_19255517, EPI_ISL_19255518, EPI_ISL_19255519, EPI_ISL_19255520, EPI_ISL_19255521, EPI_ISL_19255522, EPI_ISL_19255523, EPI_ISL_19255524, EPI_ISL_19255525, EPI_ISL_19255526, EPI_ISL_19255527, EPI_ISL_19255528, EPI_ISL_19255529, EPI_ISL_19255530, EPI_ISL_19255531, EPI_ISL_19255532, EPI_ISL_19255533, EPI_ISL_19255534, EPI_ISL_19255535, EPI_ISL_19255536, EPI_ISL_19255537, EPI_ISL_19255538, EPI_ISL_19255539, EPI_ISL_19255540, EPI_ISL_19255541			
see above	Pathogen Hunter's Research Collaborative Team	Pathogen Hunter's Research Collaborative Team	Dhammika Leshan Wannigama, Mohan Amarasiri, Shuichi Abe, Parichart Hongsing
EPI_ISL_19256187, EPI_ISL_19256189, EPI_ISL_19256190, EPI_ISL_19256191, EPI_ISL_19256199, EPI_ISL_19256227, EPI_ISL_19256236, EPI_ISL_19256237, EPI_ISL_19256251, EPI_ISL_19256253, EPI_ISL_19256256, EPI_ISL_19256277, EPI_ISL_19256278, EPI_ISL_19256280, EPI_ISL_19256284, EPI_ISL_19256286, EPI_ISL_19256288, EPI_ISL_19256292, EPI_ISL_19256294			
see above	Nigeria Centre for Disease Control and Prevention	Institute of Ecology and Evolution, University of Edinburgh	Parker,E., Omah,I.F., Varilly,P., Magee,A., Ayinla,A.O., Sijuwola,A.E., Ahmed,M.I., Ope-ewe,O.O., Ogunsanya,O.A., Olono,A., Eromon,P., Tomkins-Tinch,C.H., Otieno,J.R., Akanbi,O., Egwuenu,A., Ehiakhamen,O., Chukwu,C., Suleiman,K., Akinpelu,A., Ahmad,A., Imam,K.I., Ojedele,R., Oripenaye,V., Ikeata,K., Adelakun,S., Olajumoke,B., Djuicy,D.D., Messanga Essengue,L.L., Moumbeket Yifomnjou,M.H., Zeller,M., Gangavarapu,K., O'Toole,A., Park,D.J., Mboowa,G., Tessema,S.K., Tebeje,Y.K., Folarin,O., Happi,A., Lemey,P., Suchard,M.A., Andersen,K.G., Sabeti,P., Rambaut,A., Njoum,R., Ihekweazu,C., Jide,I., Adetifa,I. and Happi,C.T.
